# Mutually Isomeric 2- and 4-(3-Nitro-1,2,4-triazol-1-yl)pyrimidines Inspired by an Antimycobacterial Screening Hit: Synthesis and Biological Activity against the ESKAPE Panel of Pathogens

**DOI:** 10.3390/antibiotics9100666

**Published:** 2020-10-01

**Authors:** Sergey Chuprun, Dmitry Dar’in, Elizaveta Rogacheva, Liudmila Kraeva, Oleg Levin, Olga Manicheva, Marine Dogonadze, Tatiana Vinogradova, Olga Bakulina, Mikhail Krasavin

**Affiliations:** 1 Institute of Chemistry, Saint Petersburg State University, 199034 Saint Petersburg, Russia; sergej.chuprun@mail.ru (S.C.); d.dariin@spbu.ru (D.D.); o.levin@spbu.ru (O.L.); o.bakulina@spbu.ru (O.B.); 2 Pasteur Institute of Epidemiology and Microbiology, 14 Mira Street, 197101 Saint Petersburg, Russia; elizvla@yandex.ru (E.R.); lykraeva@yandex.ru (L.K.); 3 Saint Petersburg Research Institute of Phthisiopulmonology, 2-4 Ligovsky Prospekt, 191036 Saint Petersburg, Russia; olgamanicheva@rambler.ru (O.M.); marine-md@mail.ru (M.D.); vinogradova@spbniif.ru (T.V.); 4 Institute of Living Systems, Immanuel Kant Baltic Federal University, 236016 Kaliningrad, Russia

**Keywords:** bioreducible prodrugs, nitroazoles, 3-nitro-1,2,4-triazole, nucleophilic aromatic substitution, antimycobacterial activity, ESKAPE pathogens, cyclic voltammetry, reversible single-electron reduction

## Abstract

Starting from the structure of antimycobacterial screening hit OTB-021 which was devoid of activity against ESKAPE pathogens, we designed, synthesized and tested two mutually isomeric series of novel simplified analogs, 2- and 4-(3-nitro-1,2,4-triazol-1-yl)pyrimidines, bearing various amino side chains. These compounds demonstrated a reverse bioactivity profile being inactive against *M. tuberculosis* while inhibiting the growth of all ESKAPE pathogens (with variable potency patterns) except for Gram-negative *P. aeruginosa*. Reduction potentials (E_1/2_, V) measured for selected compounds by cyclic voltammetry were tightly grouped in the −1.3–−1.1 V range for a reversible single-electron reduction. No apparent correlation between the E_1/2_ values and the ESKAPE minimum inhibitory concentrations was established, suggesting possible significance of other factors, besides the compounds’ reduction potential, which determine the observed antibacterial activity. Generally, more negative E_1/2_ values were displayed by 2-(3-nitro-1,2,4-triazol-1-yl)pyrimidines, which is in line with the frequently observed activity loss on moving the 3-nitro-1,2,4-triazol-1-yl moiety from position 4 to position 2 of the pyrimidine nucleus.

## 1. Introduction

**Figure 1:**
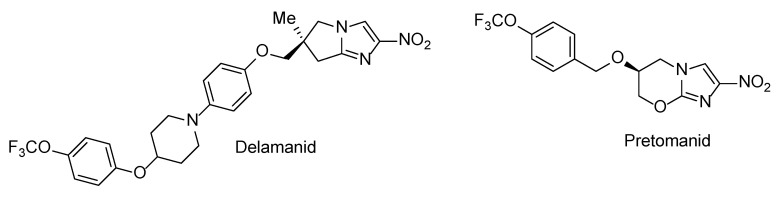
Clinically approved drugs delamanid and pretomanid.

*N*-Aryl-*C*-nitroazoles represent a general class of heterocyclic compounds which have found utility as pesticides [1], herbicides, fungicides [2] and high-energy materials [3,4,5]. At the same time, compounds belonging to this broadly defined chemical class are noticeably underrepresented in the medicinal chemistry literature [6]. Possible reasons for this include the negative stigma associated with nitro heteroaromatic moieties in general which are redox-active moieties and can therefore exert non-specific toxicity and mutagenicity [7]. Although nowadays such moieties continue being avoided, there is a steadily growing sentiment (particularly in the antibacterial field [8,9,10]) that the toxic effects of nitro heteroaromatics to the human host can be alleviated—and detrimental effects to the pathogen retained or even increased—by careful optimization of the molecular periphery around the nitro heteroaromatic warhead. The feasibility of such an approach has been demonstrated by the recent approval of antitubercular nitroimidazole drugs delamanid [11] and pretomanid [12] (Figure 1) which act as metabolically activated prodrugs. The progress in this field as well as the pros and cons of incorporating nitro (hetero)aromatic groups in drug candidate molecules have been comprehensively summarized in a recent review [13].

**Figure 2:**
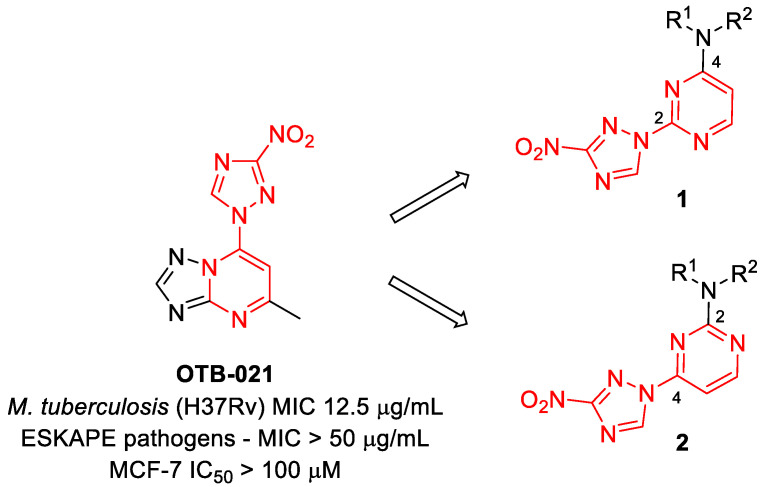
Structure of OTB-021 hit compound and the two isomeric series of simplified analogs (**1** and **2**) investigated in this work.

In our program aimed at discovering new efficacious antibacterial leads, we undertook screening of a set of diverse nitrogen heteroaromatic compounds bearing a bioreducible nitro group. From this effort, compound OTB-021 (5-methyl-7-(3-nitro-1,2,4-triazol-1-yl)-1,2,4-triazolo[1,5-*a*]pyrimidine) surfaced as a moderately potent hit with specific activity against drug-sensitive H37Rv strain of *Mycobacterium tuberculosis* [14], while other Gram-positive (*S. aureus* and *E. faecium*) or Gram-negative (*E. coli*, *P. aeruginosa*, *A. baumannii*, *K. pneumoniae*) pathogens belonging to the so-called ESKAPE panel [15] and immortalized cancer cell lines (MCF-7, FS4-LTM, KB-3-1, L929) were not affected. We hypothesized that the 4-(3-nitro-[1,2,4]-triazol-1-yl)pyrimidine portion of OTB-021 (highlighted in red) was likely responsible for its antibacterial properties, and we sought to verify this premise by simplifying the structure of this hit molecule, making it more amenable to structural variations and investigating the structure–activity relationships (SAR). To this end, we designed two isomeric series, -2- and 4-(3-nitro-1,2,4-triazol-1-yl)pyrimidines **1** and **2**, bearing diverse amino side chains in positions 4 and 2 of the pyrimidine ring, respectively. The principal idea behind such a design was to reduce the bicyclic aromatic nitrogen-rich core of OTB-021 to the more druglike pyrimidine as well as to provide sufficient room for structural diversity via the side chain variation (Figure 2). Herein, we report the synthesis and comparative evaluation of the two novel series of compounds with respect to their ability to inhibit the growth of bacterial pathogens.

## 2. Results and Discussion

### 2.1. Synthesis

#### 2.1.1. 2-(3-Nitro-1,2,4-triazol-1-yl)pyrimidines **1**

**Scheme 1:**
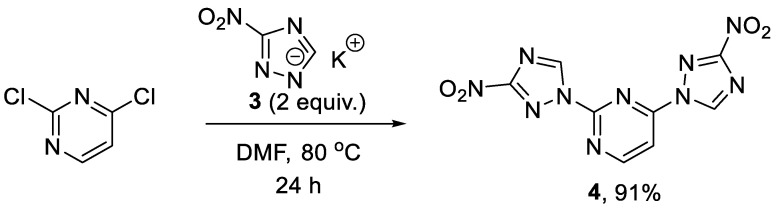
Preparation of 2,4-bis(3-nitro-1*H*-1,2,4-triazol-1-yl)pyrimidine (**4**).

Initially, our synthetic efforts focused on the installation of the 3-nitro-1,2,4-triazol-1-yl unit on the pyrimidine nucleus using commercially available 5-nitro-1*H*-1,2,4-triazole and 2,4-dichloropyrimidine. Firstly, it was promptly established that the reaction did not require the use of a metal-based catalyst (Pd^0^ or Cu^I^) and proceeded as direct nucleophilic aromatic (S*_N_*Ar) substitution. Secondly, performing the reaction in the presence of even a slight excess of the base diminished the yield as it led to the degradation of the 2,4-dichloropyrimidine starting material. Hence, 5-nitro-1*H*-1,2,4-triazole potassium salt (**3**) was obtained [16] in a separate step and used in the chloride displacement reactions. Finally, we discovered that achieving a good yield of the monosubstitution product was not straightforward as even at low conversions of the monosubstitution, the product formed reacted with the second equivalent of **3**, even in the presence of unreacted 2,4-dichloropyrimidine. Considering that the 3-nitro-1,2,4-triazol-1-yl moiety itself can served as a good leaving group in S*_N_*Ar reactions [17], we obtained disubstituted product **4** in excellent yield (Scheme 1) and subsequently employed it in S*_N_*Ar reactions with amines.

The S*_N_*Ar reactions of **4** with aliphatic amines indeed proceeded rather smoothly at room temperature in acetonitrile, while more forcing conditions (DMSO, 100 °C, 24–48 h) had to be applied with aromatic amines. The reactions displayed a pronounced selectivity toward the displacement of the 3-nitro-1*H*-1,2,4-triazol-1-yl leaving group in position 4 of the pyrimidine nucleus and allowed obtaining satisfactory yields of compounds **1a-s** (Table 1). Chromatographic isolation of sufficiently pure (>90% of purity according to ^1^H NMR) regioisomeric products **2** from these reactions was not feasible; hence, 4-(3-nitro-1,2,4-triazol-1-yl)pyrimidines were accessed via a different strategy.

**Figure 3:**
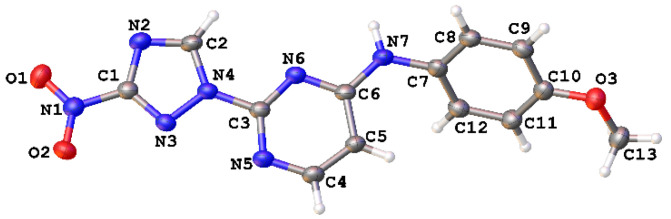
Single-crystal X-ray structure for compound **1e** (CCDC 1582454); thermal ellipsoids are shown at 50% probability.

The regiochemistry of series **1** was unequivocally confirmed by the single-crystal X-ray structure obtained for compound **1e** (Figure 3, Table S1).

#### 2.1.2. 4-(3-Nitro-1,2,4-triazol-1-yl)pyrimidines **2**

**Scheme 2:**
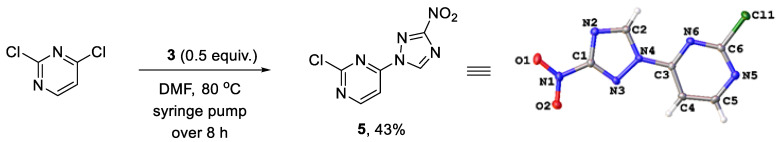
Synthesis of 2-chloro-4-(3-nitro-1*H*-1,2,4-triazol-1-yl)pyrimidine (**5**) and its single-crystal X-ray structure (CCDC 1582455, thermal ellipsoids are shown at 50% probability).

We reasoned that the seemingly unavoidable formation of disubstituted product **4** in reactions of **3** with 2,4-dichloropyrimidine could be circumvented if excess of the latter was used while the concentration of **3** was maintained low over the course of the reaction. After substantial experimentation, we established that with 0.5 equiv. of **3** added as a DMF solution to a solution of 2,4-dichloropyrimidine in DMF using a syringe pump over 8 h (at a rate of about 0.82 mmol in 2.5 mL per hour) at 80 °C, monosubstituted 2-chloro-4-(3-nitro-1*H*-1,2,4-triazol-1-yl)pyrimidine (**5**) could be obtained in 43% yield (from **3**), while unreacted 2,4-dichloropyrimidine could be isolated and utilized again. The substitution pattern of **5** was confirmed by the single-crystal X-ray analysis (Scheme 2, Table S1).

With sufficient amounts of **5** in hand, we proceeded in preparing compounds belonging to series **2** with the same set of amines as was used to prepare series **1** (to allow for direct comparison of the biological effects exerted by the two series). The substitution of the chlorine atom in position 2 with aliphatic amines generally gave moderate to high yields in acetonitrile at room temperature, while anilines, again, required heating at 100 °C in DMSO for the reaction to proceed (Table 2). Notably, in this case, no substitution of the 3-nitro-1,2,4-triazol-1-yl group in position 4 of the pyrimidine nucleus was observed.

**Figure 4:**
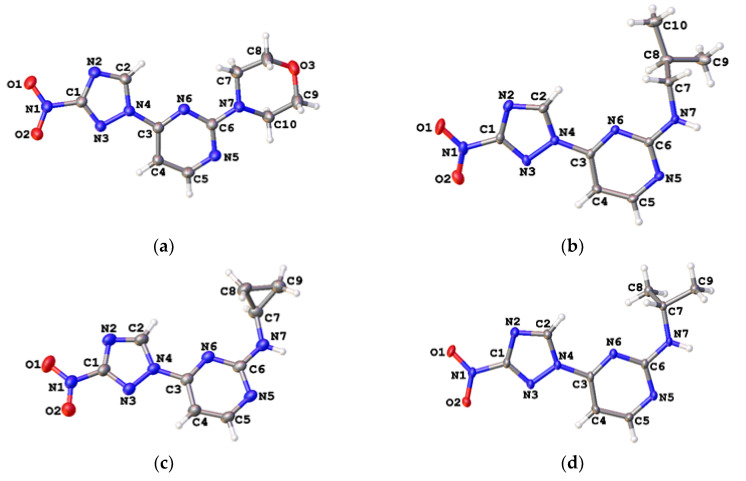
Single-crystal X-ray structures for compound **2h** (**a**, CCDC 1582450), **2k** (**b**, CCDC 1582440), **2n** (**c**, CCDC 1582442) and **2o** (**d**, CCDC 1582439); thermal ellipsoids are shown at 50% probability.

The regiochemistry of series **2** was unequivocally confirmed by the single-crystal X-ray structures obtained for compounds **2h**, **2k**, **2n** and **2o** (Figure 4, Tables S2 and S3).

### 2.2. In Vitro Biological Evaluation

Surprisingly, when tested against the drug-sensitive H37Rv strain of *Mycobacterium tuberculosis*, none of the 38 compounds, **1a-s** and **2a-s,** displayed any appreciable activity. Gratifyingly, when screened against the ESKAPE panel of six bacterial pathogens commonly found to carry antimicrobial resistance genes [15], both series displayed a promising antibacterial profile, which is summarized in Table 3 ((bis(3-nitro-1,2,4-triazol-1-yl) compound **4** is shown for comparison and ciprofloxacin was employed as a positive control for all six microorganisms).

Several important observations emerge from the data presented in Table 3. Firstly, (bis(3-nitro-1,2,4-triazol-1-yl) compound **4** was virtually inactive (except for marginal activity on *E. faecium*). This clearly demonstrates that the combination of only one 3-nitro-1,2,4-triazol-1-yl moiety with an electron-donating amino substituent is the correct definition of the pharmacophore of both series **1** and **2**. Secondly, there appears no apparent biologic activity dependence on the relative position of the two groups around the pyrimidine nucleus. Quite frequently, the high antibacterial potency of series **2** compounds was lost on switching to series **1** (cf. **2a →1a**, **2b→1b**, **2f→1f** vs. *K. pneumonia*; **2e→1e**, **2k→1k**, **2s→1s** vs. *A. baumannii*; **2e→1e** vs. *S. aureus*; **2n→1n** vs*. E. faecium*; **2b→1b** vs. *E. aerogenes*). Less frequently, the opposite was true and series **1** was highly active, while series **2** was not (cf. **1i→2i** and **1m→2m** vs. *S. aureus*; **1j→2j** and **1r→2r** vs. *A. baumannii*; **1o→2o** vs. *S. aureus* and *E. faecium*). It is an accepted view that the antibacterial activity of bioreducible nitro heteroaromatic compounds with respect to a particular bacterial species, among other factors, will depend on their ability to be metabolically activated by the membrane-bound nitro reductase enzyme of that species [18] as well as on the ability of the resulting reactive chemical entity to cross the bacterial membrane and damage the pathogen’s DNA [19]. Considering such a multifactorial nature of the observed inhibitory effects on bacterial growth, it is unsurprising that no definitive bioactivity pattern between series **1** and **2** emerged.

Other notable features of the bioactivity profile of the compounds **1a-s** and **2a-s** include the complete absence of activity against *P. aeruginosa* and the distinct susceptibility of *A. baumannii* to many compounds in the investigated set. In fact, some of the compounds (cf. **1b**, **1c**, **1j**, **1l**, **1m**, **1q**, **2b**, **2e**, **2k**, **2s**) displayed MIC values comparable or even lower than those displayed by ciprofloxacin towards this particular pathogen. Another pathogen that demonstrated susceptibility to a number of compounds tested is *E. faecium*. However, the best MIC values achieved in this case (2 µg/mL) are six times lower than the respective value for ciprofloxacin. At the same time, the activity of compounds **1i** and **1o** is only three times lower with respect to *S. aureus* than that of the comparator drug. Compound **2b** certainly leads the way in terms of the single-digit µg/mL activity displayed across the panel, strongly inhibiting the growth of *E. faecium*, *K. pneumonia*, *A. baumannii* and *E. aerogenes*. In contrast, compounds **2c**, **2k**, **1l** and **2o** appear to be distinctly selective towards *A. baumannii*, which is a characteristic tendency of the entire set. The complete absence of activity across the ESKAPE panel displayed by compound **1n** is somewhat surprising and, again, attests to the multifactorial nature of the bioactivity patterns observed.

### 2.3. Electrochemical Behavior

Since it was hypothesized that, like other nitro (hetero)aromatic drugs [20], (3-nitro-1,2,4-triazol-1-yl)pyrimidines **1** and **2** investigated in this work are activated via the nitro group reduction by the bacterial nitroreductase, we investigated the electrochemical behavior of selected compounds from both series using cyclic voltammetry. The aim of this effort was to establish if there is an apparent correlation of the antibacterial properties against any bacterial species (as was reported for some antimicrobial nitroaromatic compounds [21] as well as metal chelate complexes [22]) and the compounds’ reduction potential. Additionally, we thought it interesting to obtain a pairwise comparison of the reduction potential of series **1** and **2** compounds which might shed light on some of the bioactivity trends noted above.

**Figure 5:**
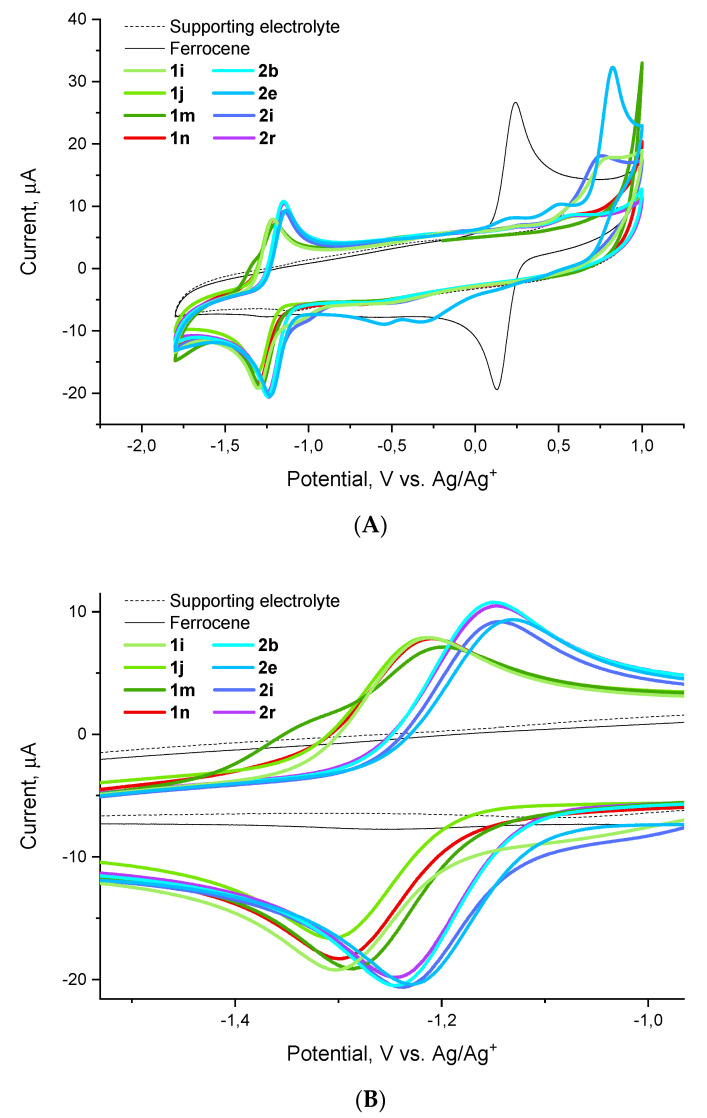
Cyclic voltammograms for compounds **1i**, **1j**, **1m**, **1n**, **2b**, **2e**, **2i** and **2r** obtained relative to the ferrocene standard: (**A**) full view, (**B**) redox wave close-up.

Considering the apparent difference in the activity trends against different pathogens of the ESKAPE panel, we focused on the activity against one specific pathogen, *E. faecium*, in nominating compounds for cyclic voltammetry experiments. To this end, we selected highly potent (**1i**, **1j**, **2i**), moderately potent (**1m**, **2b**, **2e**, **2r**) and inactive (**1n**) compounds with respect to this pathogen. Cyclic voltammograms obtained for these compounds relative to the ferrocene external standard demonstrate a one-electron reduction (HetArNO**_2_**→HetArNO**_2_^−^**) and a reversible oxidation (HetArNO**_2_^−^**→HetArNO**_2_**) wave with reduction potentials (E_1/2_) tightly grouped in the −1.3–−1.1 V range (Figure 5).

As it follows from the data collated in Table 4, there appears no apparent correlation between the activity displayed by the nine compounds investigated against *E. faecium* and their reduction potential. A brief glance at the activity data against the other five pathogens revealed that there is no correlation with the reduction potential either. This strongly suggests that, as noted previously, there are likely other factors at play, besides the compounds’ reduction potential, which determine the observed antibacterial activity. This being said, however, one can note the generally more negative E_1/2_ values observed for the series **1** compounds. All other factors being equal, this (i.e., the lower tendency of compounds **1** to undergo a one-electron reduction compared to compounds **2**) could be the likely reason for the frequently observed activity loss on switching from series **2** to series **1**.

## 3. Conclusions

Starting from the structure of antimycobacterial screening hit OTB-021 which was devoid of activity against ESKAPE pathogens, we designed, synthesized and tested two mutually isomeric series of novel simplified analogs, 2- and 4-(3-nitro-1,2,4-triazol-1-yl)pyrimidines (series **1** and **2**), bearing various amino side chains. These compounds demonstrated a reverse bioactivity profile being inactive against *M. tuberculosis* while inhibiting the growth of all ESKAPE pathogens (with variable potency patterns) except for Gram-negative *P. aeruginosa*. The observed inhibitory patterns allowed drawing some generalizations. In particular, frequent loss of activity on switching from series **2** to series **1** with the same substituents was noted (although, less frequently, the opposite trend was observed). Measurement of the reduction potentials (E_1/2_) by cyclic voltammetry for compounds selected based on their activity against *E. faecium* revealed that all compounds investigated displayed a reversible single-electron reduction with the E_1/2_ values tightly grouped in the −1.3–−1.1 V range. No apparent correlation between the E_1/2_ values and the ESKAPE minimum inhibitory concentrations was established, suggesting possible significance of other factors, besides the compounds’ reduction potential, which determine the observed antibacterial activity. However, the frequent SAR trend noted above (the absence of activity for series **1** analogs while series **2** counterparts are active) correlates with the generally more negative E_1/2_ values displayed by series **1**. Collectively, these findings fortify the position of bioreducible nitro heteroaromatic chemotypes as antibacterial leads.

## 4. Materials and Methods

### 4.1. General Experimental

All commercial reagents and solvents were used without further purification, unless otherwise noted. Analytical thin-layer chromatography was carried out on UV-254 silica gel plates using appropriate eluents. Compounds were visualized with short-wavelength UV light. NMR spectroscopic data were recorded with a Bruker Avance 400 spectrometer (400.13 MHz for ^1^H and 100.61 MHz for ^13^C) DMSO-*d*_6_ and were referenced to the residual solvent proton signal (2.51 ppm,) and solvent carbon signal (39.5 ppm). Melting points were determined with a Stuart SMP50 instrument in open capillary tubes. Mass spectra were recorded with a Bruker Maxis HRMS-ESI-qTOF spectrometer (electrospray ionization mode). Electrochemical measurements were performed with the Autolab PGSTAT30 (EcoChemie, The Netherlands) potentiostat/galvanostat.

### 4.2. Synthetic Organic Chemistry

#### 4.2.1. Preparation of 2,4-bis(3-nitro-1*H*-1,2,4-triazol-1-yl)pyrimidine (**4**)

A round-bottom flask equipped with a magnetic stir bar was charged with 2,4-dichloropyrimidine (3 g, 20.14 mmol), potassium 3-nitro-1,2,4-triazol-1-ide (**3**, 6.13 g, 40.28 mmol) and dry DMF (40 mL). The reaction mixture was stirred at 80 °C for 24 h, cooled to room temperature and concentrated under reduced pressure. The residue was taken up in distilled water (100 mL) and the resulting suspension was filtered. The solid was washed with another distilled water (100 mL) and air-dried to afford the title compound (5.61 g, 92%) as a beige solid, mp 246–248 °C. ^1^H NMR (400 MHz, DMSO-*d*_6_) δ 10.20 (s, 1H, triazole), 10.14 (s, 1H, triazole), 9.30 (d, *J* = 5.5 Hz, 1H, H_Ar_), 8.18 (d, *J* = 5.5 Hz, 1H, H_Ar_). ^13^C NMR (101 MHz, DMSO-*d*_6_) δ 164.7, 163.8, 163.7, 155.7, 153.0, 148.2, 147.2, 110.4. HRMS-ESI (*m/z*): calcd for C_8_H_4_N_10_KO_4_ [M + K]: 343.0049; found: 343.0055.

#### 4.2.2. General Procedure 2 (GP2) for the Preparation of Compounds **1a-d**, **1f-l** and **1n-s**

A glass screw-capped vial containing a magnetic stir bar was charged with 2,4-bis(3-nitro-1*H*-1,2,4-triazol-1-yl)pyrimidine (**4**) (0.98 mmol, 300 mg), amine (1.97 mmol) and acetonitrile (3 mL). The suspension was stirred at room temperature. After completion of the reaction (TLC analysis), the solvent was removed in vacuo. The resulting solid was suspended in water (5 mL) and the suspension was placed in a fridge. After 2 h, the resulting thick precipitate was filtered off, dissolved in ethyl acetate, absorbed on silica gel (ca 0.3 g) and loaded on a silica gel chromatographic column. Elution with ethyl acetate‒*n*-hexane (1:2) afforded an analytically pure product.

##### 2-((2-(3-Nitro-1*H*-1,2,4-triazol-1-yl)pyrimidin-4-yl)amino)ethan-1-ol (**1a**)

Prepared according to GP2 using 2-aminoethan-1-ol. Beige solid, yield 65%; mp 212–214 °C (decomp.). ^1^H NMR (400 MHz, DMSO-*d*_6_) δ 9.48 (s, 1H, triazole), 8.15 (d, *J* = 6.0 Hz, 1H, H_Ar_), 7.91 (s, 1H, NH), 6.67 (d, *J* = 6.0 Hz, 1H, H_Ar_), 4.54 (s, 1H, OH), 3.63 (t, *J* = 5.8 Hz, 2H, CH**_2_**), 3.58–3.38 (m, 2H, CH**_2_**); ^13^C NMR (101 MHz, DMSO-*d*_6_) δ 163.7, 163.2, 154.9, 153.6, 146.5, 107.1, 59.8, 43.2. HRMS-ESI (*m/z*): calcd for C**_8_**H**_9_**N**_7_**NaO**_3_** [M + Na]: 274.0659; found: 274.0667.

##### *N*,*N*-Dimethyl-2-(3-nitro-1*H*-1,2,4-triazol-1-yl)pyrimidin-4-amine (**1b**)

Prepared according to GP2 using dimethylamine. Yellow solid, yield 48%; mp 207–209 °C. ^1^H NMR (400 MHz, DMSO-*d*_6_) δ 9.56 (s, 1H, triazole), 8.28 (d, *J* = 6.2 Hz, 1H, H_Ar_), 6.81 (d, *J* = 6.2 Hz, 1H, H_Ar_), 3.19 (s, 6H, CH**_3_**). ^13^C NMR (126 MHz, DMSO-*d*_6_) δ 163.2, 162.8, 156.5, 153.1, 146.7, 104.0, 37.8, 37.1. HRMS-ESI (*m/z*): calcd for C_8_H_9_N_7_NaO_2_ [M + Na]: 258.0710; found: 258.0721.

##### *N*-Methyl-2-(3-nitro-1*H*-1,2,4-triazol-1-yl)pyrimidin-4-amine (**1c**)

Prepared according to GP2 using methanamine. Beige solid, yield 61%; mp 235–237 °C (decomp.). ^1^H NMR (400 MHz, DMSO-*d*_6_) δ 9.50 (s, 1H, triazole), 8.16 (d, *J* = 5.6 Hz, 1H, H_Ar_), 7.98–7.83 (s, 1H, NH), 6.61 (d, *J* = 6.0 Hz, 1H, H_Ar_), 2.95 (d, *J* = 4.8 Hz, 3H, CH**_3_**). ^13^C NMR (101 MHz, DMSO-*d*_6_) δ 168.8, 167.9, 159.5, 158.4, 151.1, 111.7, 32.2. HRMS-ESI (*m/z*): calcd for C**_7_**H**_8_**N**_7_**O**_2_** [M + H]: 222.0734; found: 222.0729.

##### *N*-Benzyl-*N*-methyl-2-(3-nitro-1*H*-1,2,4-triazol-1-yl)pyrimidin-4-amine (**1d**)

Prepared according to GP2 using *N*-methyl-1-phenylmethanamine. Green solid, yield 68%; mp 131–133 °C (decomp.). ^1^H NMR (400 MHz, DMSO-*d*_6_) δ 9.56 (s, 1H, triazole), 8.31 (d, *J* = 6.1 Hz, 1H, H_Ar_), 7.44–7.22 (m, 5H, H_Ar_), 6.87 (d, *J* = 6.2 Hz, 1H, H_Ar_), 4.92 (s, 2H, CH**_2_**), 3.19 (s, 3H, CH**_3_**). ^13^C NMR (101 MHz, DMSO) δ 163.1, 157.0, 153.3, 146.5, 137.3, 129.0, 127.8, 127.8, 104.2, 52.8, 36.1. HRMS-ESI (*m/z*): calcd for C**_14_**H**_13_**N**_7_**NaO**_2_** [M + Na]: 334.1023; found: 334.1034.

##### 3-((2-(3-Nitro-1*H*-1,2,4-triazol-1-yl)pyrimidin-4-yl)amino)propan-1-ol (**1f**)

Prepared according to GP2 using 3-aminopropan-1-ol. White solid, yield 35%; mp 201–203 °C (decomp.). ^1^H NMR (400 MHz, DMSO-*d*_6_) δ 9.48 (s, 1H,triazole), 8.15 (d, *J* = 5.9 Hz, 1H, H_Ar_), 7.91 (s, 1H, NH), 6.62 (d, *J* = 6.0 Hz, 1H, H_Ar_), 4.29 (t, *J* = 5.2 Hz, 1H, OH), 3.54 (m, 2H, CH_2_), 3.50–3.38 (m, 2H, CH**_2_**), 1.78 (q, *J* = 6.6 Hz, 2H, CH**_2_**). ^13^C NMR (101 MHz, DMSO-*d*_6_) δ 163.6, 163.2, 154.9, 153.6, 146.5, 106.9, 58.8, 37.7, 32.3. HRMS-ESI (*m/z*): calcd for C**_9_**H**_11_**N**_7_**NaO**_3_** [M + Na]: 288.0816; found: 288.0823.

##### *N*-Ethyl-2-(3-nitro-1*H*-1,2,4-triazol-1-yl)pyrimidin-4-amine (**1g**)

Prepared according to GP2 using ethanamine. White solid, yield 46%; mp 171–173 °C (decomp.). ^1^H NMR (400 MHz, DMSO-*d*_6_) δ 9.48 (s, 1H, triazole), 8.16 (d, *J* = 6.1 Hz, 1H, H_Ar_), 7.94 (s, 1H, NH), 6.60 (d, *J* = 6.0 Hz, 1H, H_Ar_), 3.44 (t, *J* = 6.9 Hz, 2H, CH**_2_**), 1.21 (t, *J* = 7.2 Hz, 3H, CH**_3_**). ^13^C NMR (101 MHz, DMSO-*d*_6_) δ 163.4, 163.2, 154.9, 153.7, 146.5, 106.9, 35.4, 14.6. HRMS-ESI (*m/z*): calcd for C**_8_**H**_9_**N**_7_**NaO**_2_** [M + Na]: 258.0710; found: 258.0704.

##### 4-(2-(3-Nitro-1*H*-1,2,4-triazol-1-yl)pyrimidin-4-yl)morpholine (**1h**)

Prepared according to GP2 using morpholine. White solid, yield 38%; mp 212–214 °C (decomp.). ^1^H NMR (400 MHz, DMSO-*d*_6_) δ 9.76 (s, 1H, triazole), 8.66 (d, *J* = 5.2 Hz, 1H, H_Ar_), 7.10 (d, *J* = 5.2 Hz, 1H, H_Ar_), 3.86 (dd, *J* = 5.6, 4.1 Hz, 4H, CH_2_), 3.72 (dd, *J* = 5.7, 4.1 Hz, 4H, CH_2_). ^13^C NMR (101 MHz, DMSO-*d*_6_) δ 163.2, 162.5, 157.3, 153.3, 146.8, 104.2, 66.2, 44.5. HRMS-ESI (*m/z*): calcd for C**_10_**H**_11_**N**_7_**NaO**_3_** [M + Na]: 300.0816; found: 300.0826.

##### 4-(4-Methylpiperazin-1-yl)-2-(3-nitro-1*H*-1,2,4-triazol-1-yl)pyrimidine (**1i**)

Prepared according to GP2 using 1-methylpiperazine. Yellow solid, yield 66%; mp 193–195 °C (decomp.). ^1^H NMR (400 MHz, DMSO-*d*_6_) δ 9.58 (s, 1H), 8.31 (d, *J* = 6.2 Hz, 1H), 6.95 (d, *J* = 6.2 Hz, 1H), 3.77 (t, *J* = 5.1 Hz, 4H), 2.48–2.41 (m, 5H), 2.27 (s, 3H). ^13^C NMR (101 MHz, DMSO-*d*_6_) δ 163.2, 162.3, 157.2, 153.3, 146.8, 104.2, 54.5, 46.0, 43.9. HRMS-ESI (*m/z*): calcd for C**_11_**H**_15_**N**_8_**O**_2_** [M + H]: 291.1312; found: 291.1321.

##### *N*-Benzyl-2-(3-nitro-1*H*-1,2,4-triazol-1-yl)pyrimidin-4-amine (**1j**)

Prepared according to GP2 using phenylmethanamine. Beige solid, yield 39%; mp 139–141 °C. ^1^H NMR (400 MHz, DMSO-*d*_6_) δ 9.49 (s, 1H, triazole), 8.46 (s, 1H, NH), 8.19 (d, *J* = 6.0 Hz, 1H, H_Ar_), 7.43–7.40 (m, 2H, H_Ar_), 7.38–7.33 (m, 2H, H_Ar_), 7.30–7.24 (m, 1H, H_Ar_), 6.68 (d, *J* = 6.0 Hz, 1H, H_Ar_), 4.66 (d, *J* = 6.0 Hz, 2H, CH**_2_**). ^13^C NMR (101 MHz, DMSO-*d*_6_) δ 163.5, 163.2, 155.4, 153.6, 146.6, 139.1, 128.9, 128.3, 127.6, 107.0, 44.0. HRMS-ESI (*m/z*): calcd for C**_13_**H**_11_**N**_7_**NaO**_2_** [M + Na]: 320.0866; found: 320.0858.

##### *N*-Isobutyl-2-(3-nitro-1*H*-1,2,4-triazol-1-yl)pyrimidin-4-amine (**1k**)

Prepared according to GP2 using 2-methylpropan-1-amine. Yellow solid, yield 32%; mp 129–131 °C. ^1^H NMR (400 MHz, DMSO-*d*_6_) δ 9.48 (s, 1H, triazole), 8.15 (d, *J* = 5.7 Hz, 1H, H_Ar_), 7.96 (s, 1H, NH), 6.65 (d, *J* = 6.0 Hz, 1H, H_Ar_), 3.26 (s, 2H, CH_2_), 1.93 (dp, *J* = 13.4, 6.7 Hz, 1H, CH), 0.97 (d, *J* = 6.7 Hz, 6H, CH_3_). ^13^C NMR (101 MHz, DMSO-*d*_6_) δ 163.8, 163.2, 155.0, 153.6, 146.5, 106.9, 47.9, 28.2, 20.6. HRMS-ESI (*m/z*): calcd for C**_10_**H**_13_**N**_7_**NaO**_2_** [M + Na]: 286.1023; found: 286.1015.

##### 2,2′-((2-(3-Nitro-1*H*-1,2,4-triazol-1-yl)pyrimidin-4-yl)azanediyl)bis(ethan-1-ol) (**1l**)

Prepared according to GP2 using 2,2′-azanediylbis(ethan-1-ol). Green solid, yield 28%; mp 163–165 °C. ^1^H NMR (400 MHz, DMSO-*d*_6_) δ 9.53 (s, 1H, triazole), 8.25 (d, *J* = 6.2 Hz, 1H, H_Ar_), 6.87 (d, *J* = 6.3 Hz, 1H, H_Ar_), 4.61 (s, 2H, OH), 3.85–3.59 (m, 8H, CH**_2_**). ^13^C NMR (101 MHz, DMSO-*d*_6_) δ 163.2, 162.7, 156.3, 153.1, 146.6, 104.7, 58.7, 51.8, 51.0. HRMS-ESI (*m/z*): calcd for C**_10_**H**_14_**N**_7_**O**_4_** [M + H]: 296.1102; found: 296.1114.

##### *N*-Cyclopropyl-2-(3-nitro-1*H*-1,2,4-triazol-1-yl)pyrimidin-4-amine (**1n**)

Prepared according to GP2 using cyclopropanamine. Yellow solid, yield 47%; mp 193–195 °C (decomp.). ^1^H NMR (400 MHz, DMSO-*d*_6_) δ 9.45 (s, 1H, triazole), 8.34–8.15 (m, 2H, NH, H_Ar_), 6.74 (s, 1H, H_Ar_), 2.80 (s, 1H, CH), 0.84 (td, *J* = 6.9, 4.7 Hz, 2H, CH**_2_**), 0.58 (dd, *J* = 4.1, 2.4 Hz, 2H, CH**_2_**). ^13^C NMR (101 MHz, DMSO-*d*_6_) δ 165.5, 164.7, 163.2, 157.4, 155.0, 153.3, 146.3, 107.0, 103.4, 23.8, 7.1, 6.5. HRMS-ESI (*m/z*): calcd for C_9_H_10_N_7_O_2_ [M + H]: 248.0890; found: 248.0901.

##### *N*-Isopropyl-2-(3-nitro-1*H*-1,2,4-triazol-1-yl)pyrimidin-4-amine (**1o**)

Prepared according to GP2 using propan-2-amine. White solid, yield 43%; mp 244–246 °C. ^1^H NMR (400 MHz, DMSO-*d*_6_) δ 9.47 (s, 1H, triazole), 8.14 (d, *J* = 6.0 Hz, 1H, H_Ar_), 7.84 (d, *J* = 7.7 Hz, 1H, NH), 6.59 (d, *J* = 6.1 Hz, 1H, H_Ar_), 4.22 (s, 1H, CH), 1.24 (d, *J* = 6.5 Hz, 6H, CH_3_). ^13^C NMR (101 MHz, DMSO-*d*_6_) δ 163.2, 162.7, 154.9, 153.7, 146.5, 106.9, 42.2, 22.6. HRMS-ESI (*m/z*): calcd for C**_9_**H**_11_**N**_7_**NaO**_2_** [M + Na]: 272.0866; found: 272.0859.

##### *N*-(3-(1*H*-Imidazol-1-yl)propyl)-2-(3-nitro-1*H*-1,2,4-triazol-1-yl)pyrimidin-4-amine (**1p**)

Prepared according to GP2 using 3-(1*H*-imidazol-1-yl)propan-1-amine. Yellow solid, yield 66%; mp 198–200 °C (decomp.). ^1^H NMR (400 MHz, DMSO-*d*_6_) δ 9.45 (s, 1H, triazole), 8.18 (d, *J* = 6.0 Hz, 1H, H_Ar_), 8.04 (s, 1H, NH), 7.61 (s, 1H, imidazole), 7.18 (d, *J* = 1.2 Hz, 1H, imidazole), 6.90 (d, *J* = 1.1 Hz, 1H, imidazole), 6.61 (d, *J* = 6.0 Hz, 1H, H_Ar_), 4.09 (t, *J* = 7.0 Hz, 2H, CH_2_), 3.40 (d, *J* = 7.1 Hz, 2H, CH_2_), 2.07 (p, *J* = 6.9 Hz, 2H, CH_2_). ^13^C NMR (101 MHz, DMSO-*d*_6_) δ 163.6, 163.2, 155.2, 153.6, 146.5, 137.8, 128.9, 119.8, 107.1, 44.1, 37.8, 30.6. HRMS-ESI (*m/z*): calcd for C**_12_**H**_14_**N**_9_**O**_2_** [M + H]: 316.1265; found: 316.1271.

##### 2-(3-Nitro-1*H*-1,2,4-triazol-1-yl)-4-(pyrrolidin-1-yl)pyrimidine (**1q**)

Prepared according to GP2 using pyrrolidine. Green solid, yield 44%; mp 219–221 °C (decomp.). ^1^H NMR (400 MHz, DMSO-*d*_6_) δ 9.64 (s, 1H, triazole), 8.61 (d, *J* = 5.2 Hz, 1H, H_Ar_), 7.03 (d, *J* = 5.2 Hz, 1H, H_Ar_), 3.69–3.54 (m, 4H, CH**_2_**), 2.06–1.91 (m, 4H, CH**_2_**). ^13^C NMR (126 MHz, DMSO-*d*_6_) δ 163.2, 160.5, 156.0, 153.2, 146.6, 105.0, 47.1, 47.0, 25.5, 24.8. HRMS-ESI (*m/z*): calcd for C_10_H_12_N_7_O_2_ [M + H]: 262.1047; found: 262.1038.

##### 2-(3-Nitro-1*H*-1,2,4-triazol-1-yl)-*N*-propylpyrimidin-4-amine (**1r**)

Prepared according to GP2 using propan-1-amine. Beige solid, yield 61%; mp 127–129 °C. ^1^H NMR (400 MHz, DMSO-*d*_6_) δ 9.47 (s, 1H, triazole), 8.15 (d, *J* = 6.0 Hz, 1H, H_Ar_), 7.95 (s, 1H, NH), 6.62 (d, *J* = 6.0 Hz, 1H, H_Ar_), 3.37 (d, *J* = 8.4 Hz, 2H, CH**_2_**), 1.63 (q, *J* = 7.2 Hz, 2H, CH_2_), 0.96 (t, *J* = 7.4 Hz, 3H, CH**_3_**). ^13^C NMR (101 MHz, DMSO-*d*_6_) δ 163.6, 163.2, 154.9, 153.6, 146.5, 106.9, 42.2, 22.3, 11.9. HRMS-ESI (*m/z*): calcd for C**_9_**H**_11_**N**_7_**NaO**_2_** [M + Na]: 272.0866; found: 272.0854.

##### *N*-(4-Methoxybenzyl)-2-(3-nitro-1*H*-1,2,4-triazol-1-yl)pyrimidin-4-amine (**1s**)

Prepared according to GP2 using (4-methoxyphenyl)methanamine. Beige solid, yield 19%; mp 147–149 °C (decomp.). ^1^H NMR (400 MHz, DMSO-*d*_6_) δ 9.51 (s, 1H, triazole), 8.18 (d, *J* = 6.0 Hz, 1H, H_Ar_), 7.39–7.30 (m, 2H, H_Ar_), 6.96–6.85 (m, 2H, H_Ar_), 6.66 (d, *J* = 6.0 Hz, 1H, H_Ar_), 4.58 (d, *J* = 5.8 Hz, 2H, CH**_2_**), 3.76 (s, 3H, CH_3_). ^13^C NMR (101 MHz, DMSO-*d*_6_) δ 163.3, 163.2, 158.9, 155.3, 153.6, 146.6, 130.9, 129.7, 114.3, 107.0, 55.5, 43.5. HRMS-ESI (*m/z*): calcd for C**_14_**H**_13_**N**_7_**NaO**_3_** [M + Na]: 350.0972; found: 350.0962.

##### *N*-(4-Methoxyphenyl)-2-(3-nitro-1*H*-1,2,4-triazol-1-yl)pyrimidin-4-amine (1**e**)

A glass screw-capped vial containing a magnetic stir bar was charged with 2,4-bis(3-nitro-1*H*-1,2,4-triazol-1-yl)pyrimidine (**4**) (0.98 mmol, 300 mg), 4-methoxyaniline (1.97 mmol, 242 mg) and DMSO (3 mL). The suspension was stirred at 100 °C for 24 h. The reaction mixture was poured into water (10 mL) and cooled in a refrigerator. The resulting precipitate was filtered and purified by silica column chromatography with ethyl acetate‒*n*-hexane (1:2) to afford a pure compound. Greenish solid, yield 45%; mp 216–218 °C. ^1^H NMR (400 MHz, DMSO-*d*_6_) δ 9.90 (s, 1H, NH), 9.44 (s, 1H, triazole), 8.32 (d, *J* = 5.9 Hz, 1H, H_Ar_), 7.62–7.48 (m, 2H, H_Ar_), 7.06–6.93 (m, 2H, H_Ar_), 6.80 (d, *J* = 6.0 Hz, 1H, H_Ar_), 3.80 (s, 3H, CH**_3_**). ^13^C NMR (126 MHz, DMSO-*d*_6_) δ 163.2, 161.7, 156.4, 153.4, 146.5, 131.9, 122.7, 114.8, 107.7, 55.7. HRMS-ESI (*m/z*): calcd for C**_13_**H**_11_**N**_7_**NaO**_3_** [M + Na]: 336.0816; found: 336.0826.

##### 2-(3-Nitro-1*H*-1,2,4-triazol-1-yl)-*N*-(p-tolyl)pyrimidin-4-amine (1**m**)

A glass screw-capped vial containing a magnetic stir bar was added to with 2,4-bis(3-nitro-1*H*-1,2,4-triazol-1-yl)pyrimidine (**4**) (0.98 mmol, 300 mg), *p*-toluidine (1.97 mmol, 211 mg) and DMSO (3 mL). The suspension was stirred at 100 °C for 48 h. The reaction mass was poured into water (25 mL) and extracted by ethyl acetate (3 × 20 mL). The combined organic solutions were washed with water and brine, dried over MgSO**_4_**, absorbed on silica gel by concentration in vacuo and loaded on a chromatographic column. Elution with ethyl acetate‒*n*-hexane (1:2) afforded the title compound. Yellow solid, yield 35%; mp 199–201 °C (decomp.). ^1^H NMR (400 MHz, DMSO-*d*_6_) δ 9.96 (s, 1H, NH), 9.45 (s, 1H, triazole), 8.35 (d, *J* = 5.9 Hz, 1H, H_Ar_), 7.64–7.51 (m, 2H, H_Ar_), 7.23 (d, *J* = 8.1 Hz, 2H, H_Ar_), 6.86 (d, *J* = 5.9 Hz, 1H, H_Ar_), 2.33 (s, 3H, CH**_3_**). ^13^C NMR (126 MHz, DMSO-*d*_6_) δ 163.2, 161.7, 156.7, 153.4, 146.5, 136.4, 133.3, 130.0, 121.2, 107.8, 20.9. HRMS-ESI (*m/z*): calcd for C**_13_**H**_11_**N**_7_**NaO**_2_** [M + Na]: 320.0866; found: 320.0874.

#### 4.2.3. Preparation of 2-chloro-4-(3-nitro-1*H*-1,2,4-triazol-1-yl)pyrimidine (**5**)

A two-neck round-bottomed flask equipped with a magnetic stir bar was charged with 2,4-dichloropyrimidine (1.96 g, 13.4 mmol) and dry DMF (5 mL). Potassium 3-nitro-1,2,4-triazol-1-ide (**3**, 1 g, 6.57 mmol) was dissolved in dry DMF (20 mL) and was added slowly via a syringe pump to a vigorously stirred solution of 2,4-dichloropyrimidine at 80 °C over 8 h. The resulting mixture was left under stirring for 16 h at 80 °C. After cooling to room temperature, the reaction mixture was concentrated under reduced pressure. The residue was dissolved in ethyl acetate and passed through a short plug of silica eluting with 50 mL more of ethyl acetate. The eluted solution was concentrated in vacuo and the crude product was purified by column chromatography on silica gel (ethyl acetate–*n*-hexane, 1:2) to give 634 mg (43%) of the title compound as a white solid, mp 143–145 °C (934 mg of starting 2,4-dichloropyrimidine was recovered by the same purification). ^1^H NMR (400 MHz, DMSO-*d*_6_) δ 9.87 (s, 1H, triazole), 9.07 (d, *J* = 5.5 Hz, 1H, H_Ar_), 8.08 (d, *J* = 5.4 Hz, 1H, H_Ar_). ^13^C NMR (101 MHz, DMSO-*d*_6_) δ 164.5, 163.6, 159.9, 156.3, 146.8, 109.8. HRMS-ESI (*m/z*): calcd for C**_6_**H**_4_**ClN**_6_**O**_2_** [M + H]: 227.0079; found: 227.0083.

#### 4.2.4. General Procedure 1 (GP1) for Preparation of Compounds **1a-d**, **1f-l** and **1n-s**

A glass screw-capped vial containing a magnetic stir bar was charged with 2-chloro-4-(3-nitro-1*H*-1,2,4-triazol-1-yl)pyrimidine (**5**) (0.44 mmol, 100 mg), amine (1.32 mmol) and acetonitrile (1 mL). The reaction mixture was stirred at room temerature. After completion of the reaction (TLC analysis), the solvent was removed in vacuo. The resulting solid was suspended in water (5 mL) and the suspension was cooled in the refrigerator. After 2 h, the thick precipitate was filtered off, air-dried and additionally cristallyzed from 96% ethanol to afford the product.

##### 2-((4-(3-Nitro-1*H*-1,2,4-triazol-1-yl)pyrimidin-2-yl)amino)ethan-1-ol (**2a**)

Prepared according to GP1 using 2-aminoethan-1-ol. Greenish solid, yield 39%; mp 200–202 °C (decomp.). 1H NMR (400 MHz, DMSO-*d*_6_) δ 9.55 (s, 1H, triazole), 8.55 (d, *J* = 5.1 Hz, 1H, H_Ar_), 7.37 (s, *J* = 7.0 Hz, ^1^H, NH), 7.03 (d, *J* = 5.2 Hz, 1H, H_Ar_), 4.44 (s, 1H, OH), 3.61 (t, *J* = 6.1 Hz, 2H, CH_2_), 3.50 (q, *J* = 5.9 Hz, 2H, CH**_2_**). ^13^C NMR (126 MHz, DMSO-*d*_6_) δ 163.3, 162.6, 162.2, 155.0, 145.4, 97.1, 59.9, 44.0. HRMS-ESI (*m/z*): calcd for C**_8_**H**_10_**N**_7_**O**_3_** [M + H]: 252.0840; found: 252.0848.

##### *N*,*N*-Dimethyl-4-(3-nitro-1*H*-1,2,4-triazol-1-yl)pyrimidin-2-amine (**2b**)

Prepared according to GP1 using dimethylamine. Greenish solid, yield 54%; mp 167–169 °C (decomp.). ^1^H NMR (400 MHz, DMSO-*d*_6_) δ 9.69 (s, 1H, triazole), 8.61 (d, *J* = 5.2 Hz, 1H, H_Ar_), 7.01 (d, *J* = 5.2 Hz, 1H, H_Ar_), 3.23 (s, 6H, CH**_3_**). ^13^C NMR (126 MHz, DMSO-*d*_6_) δ 163.3, 162.4, 161.6, 154.9, 96.5, 37.1. HRMS-ESI (*m/z*): calcd for C**_8_**H**_10_**N**_7_**O**_2_** [M + H]: 236.0890; found: 236.0881.

##### *N*-Methyl-4-(3-nitro-1*H*-1,2,4-triazol-1-yl)pyrimidin-2-amine (**2c**)

Prepared according to GP1 using methanamine. Greenish solid, yield 62%; mp 238–240 °C (decomp.). ^1^H NMR (400 MHz, DMSO-*d*_6_) δ 9.57 (s, 1H, triazole), 8.56 (d, *J* = 5.2 Hz, 1H, H_Ar_), 7.47 (s, 1H, NH), 7.03 (d, *J* = 5.1 Hz, 1H, H_Ar_), 2.94 (d, *J* = 4.8 Hz, 3H, CH**_3_**). ^13^C NMR (126 MHz, DMSO) δ 163.3, 162.9, 162.6, 155.1, 145.5, 96.9, 28.2. HRMS-ESI (*m/z*): calcd for C**_7_**H**_8_**N**_7_**O**_2_** [M + H]: 222.0734; found: 222.0742.

##### *N*-Benzyl-*N*-methyl-4-(3-nitro-1*H*-1,2,4-triazol-1-yl)pyrimidin-2-amine (**2d**)

Prepared according to GP1 using *N*-methyl-1-phenylmethanamine. Greenish solid, yield 23%; mp 145–147 °C. ^1^H NMR (400 MHz, DMSO-*d*_6_) δ 9.70 (s, 1H, triazole), 8.66 (d, *J* = 5.2 Hz, 1H, H_Ar_), 7.39–7.24 (m, 5H, H_Ar_), 7.09 (d, *J* = 5.2 Hz, 1H, H_Ar_), 4.98 (s, 2H, CH_2_), 3.21 (s, 3H, CH**_3_**). ^13^C NMR (126 MHz, DMSO-*d*_6_) δ 163.3, 162.7, 161.6, 155.0, 145.7, 138.3, 129.0, 128.1, 127.6, 97.2, 52.2, 35.4. HRMS-ESI (*m/z*): calcd for C**_14_**H**_13_**N**_7_**NaO**_2_** [M + Na]: 334.1023; found: 334.1025.

##### 3-((4-(3-Nitro-1*H*-1,2,4-triazol-1-yl)pyrimidin-2-yl)amino)propan-1-ol (**2f**)

Prepared according to GP1 using 3-aminopropan-1-ol. Greenish solid, yield 58%; mp 178–180 °C. ^1^H NMR (400 MHz, DMSO-*d*_6_) δ 9.54 (s, 1H, triazole), 8.55 (d, *J* = 5.2 Hz, 1H, H_Ar_), 7.49 (s, 1H, NH), 7.01 (d, *J* = 5.2 Hz, 1H, H_Ar_), 4.24 (t, *J* = 5.2 Hz, 1H, OH), 3.55 (td, *J* = 6.2, 5.2 Hz, 2H, CH_2_), 3.51–3.43 (m, 2H, CH_2_), 1.78 (h, *J* = 6.8 Hz, 2H, CH**_2_**). ^13^C NMR (101 MHz, DMSO-*d*_6_) δ 163.3, 162.6, 162.2, 145.4, 96.9, 59.1, 38.5, 32.5. HRMS-ESI (*m/z*): calcd for C**_9_**H**_12_**N**_7_**O**_3_** [M + H]: 266.0996; found: 266.1002.

##### *N*-Ethyl-4-(3-nitro-1*H*-1,2,4-triazol-1-yl)pyrimidin-2-amine (**2g**)

Prepared according to GP1 using ethanamine. Green solid, yield 75%; mp 229–231 °C (decomp.). ^1^H NMR (400 MHz, DMSO-*d*_6_) δ 9.53 (s, 1H, triazole), 8.55 (d, *J* = 5.2 Hz, 1H, H_Ar_), 7.52 (s, 1H, NH), 7.01 (d, *J* = 5.2 Hz, 1H, H_Ar_), 3.44 (qd, *J* = 7.1, 5.7 Hz, 2H, CH_2_), 1.20 (t, *J* = 7.2 Hz, 3H, CH**_3_**). ^13^C NMR (101 MHz, DMSO-*d*_6_) δ 163.3, 162.6, 162.3, 155.1, 145.4, 97.0, 36.0, 14.9. HRMS-ESI (*m/z*): calcd for C**_8_**H**_10_**N**_7_**O**_2_** [M + H]: 236.0890; found: 236.0897.

##### 4-(4-(3-Nitro-1*H*-1,2,4-triazol-1-yl)pyrimidin-2-yl)morpholine (**2h**)

Prepared according to GP1 using morpholine. Yellow solid, yield 83%; mp 201–203 °C (decomp.). ^1^H NMR (400 MHz, DMSO-*d*_6_) δ 9.77 (s, 1H, triazole), 8.66 (d, *J* = 5.2 Hz, 1H, H_Ar_), 7.10 (d, *J* = 5.2 Hz, 1H, H_Ar_), 3.87 (dd, *J* = 5.7, 4.1 Hz, 4H, CH_2_), 3.73 (dd, *J* = 5.7, 4.1 Hz, 4H, CH**_2_**). ^13^C NMR (101 MHz, DMSO-*d*_6_) δ 163.3, 162.7, 161.0, 155.0, 145.8, 97.7, 66.4, 44.4. HRMS-ESI (*m/z*): calcd for C**_10_**H**_11_**N**_7_**NaO**_3_** [M + Na]: 300.0816; found: 300.0828.

##### 2-(4-Methylpiperazin-1-yl)-4-(3-nitro-1*H*-1,2,4-triazol-1-yl)pyrimidine (**2i**)

Prepared according to GP1 using 1-methylpiperazine. Greenish solid, yield 76%; mp 210–212 °C (decomp.). ^1^H NMR (400 MHz, DMSO-*d*_6_) δ 9.75 (s, 1H, triazole), 8.63 (d, *J* = 5.2 Hz, 1H, H_ar_), 7.06 (d, *J* = 5.2 Hz, 1H, H_Ar_), 3.92 – 3.83 (m, 4H, CH_2_), 2.47 – 2.40 (m, 4H, CH**_2_**), 2.27 (s, 3H, CH**_3_**). ^13^C NMR (101 MHz, DMSO-*d*_6_) δ 163.3, 162.7, 160.9, 155.1, 145.8, 97.4, 54.8, 46.2, 43.9. HRMS-ESI (*m/z*): calcd for C**_11_**H**_15_**N**_8_**O**_2_** [M + H]: 291.1312; found: 291.1319.

##### *N*-Benzyl-4-(3-nitro-1*H*-1,2,4-triazol-1-yl)pyrimidin-2-amine (**2j**)

Prepared according to GP1 using benzylamine. White solid, yield 81%; mp 212–214 °C (decomp.). ^1^H NMR (400 MHz, DMSO-*d*_6_) δ 9.52 (s, 1H, triazole), 8.57 (d, *J* = 5.1 Hz, 1H, H_Ar_), 8.11 (s, 1H, NH), 7.41 (d, *J* = 7.0 Hz, 2H, H_Ar_), 7.36–7.29 (m, 2H, H_Ar_), 7.27–7.20 (m, 1H, H_Ar_), 7.05 (d, *J* = 5.1 Hz, 1H, H_Ar_), 4.64 (d, *J* = 6.3 Hz, 2H, CH_2_). ^13^C NMR (101 MHz, DMSO-*d*_6_) δ 163.3, 162.9, 162.3, 145.3, 140.2, 128.7, 128.1, 127.5, 127.2, 97.4, 44.6. HRMS-ESI (*m/z*): calcd for C**_13_**H**_12_**N**_7_**O**_2_** [M + H]: 296.1047; found: 296.1050.

##### *N*-Isobutyl-4-(3-nitro-1*H*-1,2,4-triazol-1-yl)pyrimidin-2-amine (**2k**)

Prepared according to GP1 using 2-methylpropan-1-amine. Greenish solid, yield 44%; mp 149–151 °C. ^1^H NMR (400 MHz, DMSO-*d*_6_) δ 9.53 (s, 1H, triazole), 8.54 (d, *J* = 5.1 Hz, 1H, H_Ar_), 7.57 (s, 1H, NH), 7.01 (d, *J* = 5.2 Hz, 1H, H_Ar_), 3.25 (dd, *J* = 6.8, 6.0 Hz, 2H, CH_2_), 1.94 (dt, *J* = 13.4, 6.7 Hz, 1H, CH), 0.95 (d, *J* = 6.7 Hz, 6H, CH**_3_**). ^13^C NMR (101 MHz, DMSO-*d*_6_) δ 163.3, 162.6, 155.0, 145.4, 145.0, 96.9, 48.6, 28.3, 20.6. HRMS-ESI (*m/z*): calcd for C**_10_**H**_14_**N**_7_**O**_2_** [M + H]: 264.1203; found: 264.1212.

##### 2,2′-((4-(3-nitro-1*H*-1,2,4-triazol-1-yl)pyrimidin-2-yl)azanediyl)bis(ethan-1-ol) (**2l**)

Prepared according to GP1 using 2,2′-azanediylbis(ethan-1-ol). Yellow solid, yield 71%; mp 172–174 °C. ^1^H NMR (400 MHz, DMSO-*d*_6_) δ 9.65 (s, 1H, triazole), 8.61 (d, *J* = 5.2 Hz, 1H, H_Ar_), 7.03 (d, *J* = 5.1 Hz, 1H, H_Ar_), 4.52 (s, 2H, OH), 3.80 (t, *J* = 6.2 Hz, 4H, CH**_2_**), 3.69 (d, *J* = 6.2 Hz, 4H, CH**_2_**). ^13^C NMR (126 MHz, DMSO-*d*_6_) δ 163.3, 162.4, 161.1, 154.9, 145.5, 96.8, 58.9, 51.5, 51.4. HRMS-ESI (*m/z*): calcd for C**_10_**H**_14_**N**_7_**O**_4_** [M + H]: 296.1102; found: 296.1114.

##### *N*-cyclopropyl-4-(3-nitro-1*H*-1,2,4-triazol-1-yl)pyrimidin-2-amine (**2n**)

Prepared according to GP1 using cyclopropylamine. Greenish solid, yield 49%; mp 207–209 °C. ^1^H NMR (400 MHz, DMSO-*d*_6_) δ 9.51 (s, 1H, triazole), 8.59 (d, *J* = 5.2 Hz, 1H, H_Ar_), 7.75 (d, *J* = 3.9 Hz, 1H, NH), 7.08 (d, *J* = 5.2 Hz, 1H, H_Ar_), 2.91 (td, *J* = 7.2, 3.6 Hz, 1H, CH), 0.78 (td, *J* = 7.0, 4.7 Hz, 2H, CH_2_), 0.61–0.54 (m, 2H, CH**_2_**). ^13^C NMR (101 MHz, DMSO-*d*_6_) δ 163.4, 163.3, 162.5, 155.0, 145.1, 97.8, 24.4, 6.6. HRMS-ESI (*m/z*): calcd for C**_9_**H**_10_**N**_7_**O**_2_** [M + H]: 248.0890; found: 248.0901.

##### *N*-Isopropyl-4-(3-nitro-1*H*-1,2,4-triazol-1-yl)pyrimidin-2-amine (**2o**)

Prepared according to GP1 using propan-2-amine. Green solid, yield 59%; mp 193–195 °C (decomp.). ^1^H NMR (400 MHz, DMSO-*d*_6_) δ 9.51 (s, 1H, triazole), 8.55 (d, *J* = 5.1 Hz, 1H, H_Ar_), 7.39 (d, *J* = 7.9 Hz, 1H, NH), 7.00 (d, *J* = 5.2 Hz, 1H, H_Ar_), 4.20 (dp, *J* = 7.9, 6.5 Hz, 1H, CH), 1.23 (d, *J* = 6.5 Hz, 6H, CH**_3_**). ^13^C NMR (101 MHz, DMSO-*d*_6_) δ 163.3, 162.6, 161.6, 155.1, 145.3, 96.9, 42.7, 22.6. HRMS-ESI (*m/z*): calcd for C**_9_**H**_12_**N**_7_**O**_2_** [M + H]: 250.1047; found: 250.1056.

##### *N*-(3-(1*H*-Imidazol-1-yl)propyl)-4-(3-nitro-1H-1,2,4-triazol-1-yl)pyrimidin-2-amine (**2p**)

Prepared according to GP1 using 3-(1*H*-imidazol-1-yl)propan-1-amine. Greenish solid, yield 26%; mp 196–198 °C. ^1^H NMR (400 MHz, DMSO-*d*_6_) δ 9.48 (s, 1H, triazole), 8.56 (d, *J* = 5.2 Hz, 1H, H_Ar_), 7.69 (t, *J* = 5.9 Hz, 1H, NH), 7.63 (d, *J* = 1.2 Hz, 1H, imidazole), 7.17 (d, *J* = 1.3 Hz, 1H, imidazole), 7.04 (d, *J* = 5.2 Hz, 1H, H_Ar_), 6.91 (d, *J* = 1.1 Hz, 1H, imidazole), 4.09 (t, *J* = 7.0 Hz, 2H, CH**_2_**), 3.40 (q, *J* = 6.6 Hz, 2H, CH**_2_**), 2.06 (p, *J* = 6.9 Hz, 2H, CH**_2_**). ^13^C NMR (126 MHz, DMSO-*d*_6_) δ 163.3, 162.7, 162.4, 155.0, 145.3, 137.8, 128.8, 119.8, 97.2, 56.5, 44.2, 38.3, 30.8, 19.0. HRMS-ESI (*m/z*): calcd for C**_12_**H**_14_**N**_9_**O**_2_** [M + H]: 316.1265; found: 316.1270.

##### 4-(3-Nitro-1*H*-1,2,4-triazol-1-yl)-2-(pyrrolidin-1-yl)pyrimidine (**2q**)

Prepared according to GP1 using pyrrolidine. White solid, yield 40%; mp 188–190 °C (decomp.). ^1^H NMR (400 MHz, DMSO-*d*_6_) δ 9.64 (s, 1H, triazole), 8.61 (d, *J* = 5.2 Hz, 1H, H_Ar_), 7.02 (d, *J* = 5.2 Hz, 1H, H_Ar_), 3.67–3.57 (m, 4H, CH**_2_**), 2.06–1.94 (m, 4H, CH**_2_**). ^13^C NMR (101 MHz, DMSO-*d*_6_) δ 163.3, 162.4, 159.6, 154.8, 145.4, 96.5, 47.0, 25.4, 25.2. HRMS-ESI (*m/z*): calcd for C**_10_**H**_12_**N**_7_**O**_2_** [M + H]: 262.1047; found: 262.1053.

##### 4-(3-Nitro-1*H*-1,2,4-triazol-1-yl)-*N*-propylpyrimidin-2-amine (**2r**)

Prepared according to GP1 using propan-1-amine. White solid, yield 49%; mp 175–177 °C (decomp.). ^1^H NMR (400 MHz, DMSO-*d*_6_) δ 9.53 (s, 1H, triazole), 8.55 (d, *J* = 5.2 Hz, 1H, H_Ar_), 7.54 (s, 1H, NH), 7.01 (d, *J* = 5.1 Hz, 1H, H_Ar_), 3.44–3.32 (m, 2H, CH**_2_**), 1.62 (h, *J* = 7.3 Hz, 2H, CH**_2_**), 0.95 (t, *J* = 7.4 Hz, 3H, CH**_3_**). ^13^C NMR (101 MHz, DMSO-*d*_6_) δ 163.3, 162.5, 162.2, 155.0, 145.4, 96.9, 42.9, 22.5, 11.9. HRMS-ESI (*m/z*): calcd for C**_9_**H**_12_**N**_7_**O**_2_** [M + H]: 250.1047; found: 250.1058.

##### *N*-(4-Methoxybenzyl)-4-(3-nitro-1*H*-1,2,4-triazol-1-yl)pyrimidin-2-amine (**2s**)

Prepared according to GP1 using (4-methoxyphenyl)methanamine. Greenish solid, yield 46%; mp 248–250 °C (decomp.). ^1^H NMR (400 MHz, DMSO-*d*_6_) δ 9.54 (s, 1H, triazole), 8.57 (d, *J* = 5.2 Hz, 1H, H_Ar_), 8.02 (s, 1H, NH), 7.38–7.29 (m, 2H, H_Ar_), 7.04 (d, *J* = 5.2 Hz, 1H, H_Ar_), 6.93–6.84 (m, 2H, H_Ar_), 4.56 (d, *J* = 6.2 Hz, 2H, CH**_2_**), 3.74 (s, 3H, CH**_3_**). ^13^C NMR (101 MHz, DMSO-*d*_6_) δ 163.3, 162.8, 162.3, 158.6, 145.3, 132.1, 129.4, 128.9, 114.1, 97.3, 55.5, 44.0. HRMS-ESI (*m/z*): calcd for C**_14_**H**_13_**N**_7_**NaO**_3_** [M + Na]: 350.0972; found: 350.0973.

##### *N*-(4-Methoxyphenyl)-4-(3-nitro-1*H*-1,2,4-triazol-1-yl)pyrimidin-2-amine (2**e**)

A glass screw-capped vial containing a magnetic stir bar was charged with 2-chloro-4-(3-nitro-1*H*-1,2,4-triazol-1-yl)pyrimidine (**5**, 0.44 mmol, 100 mg), 4-methoxyaniline (1.32 mmol, 163 mg) and DMSO (2 mL). The reaction mixture was stirred at 100 °C for 3 h. The reaction mixture was poured into water (10 mL) and cooled in a refrigerator. The resulting precipitate was filtered off and additionally crystallized from 96% ethanol to afford the title compound. Orange solid, yield 41%; mp 236–238 °C (decomp.). ^1^H NMR (500 MHz, DMSO-*d*_6_) δ 9.71 (s, 1H, NH), 9.46 (s, 1H, triazole), 8.70 (d, *J* = 5.2 Hz, 1H, H_Ar_), 7.74–7.59 (m, 2H, H_Ar_), 7.23 (d, *J* = 5.2 Hz, 1H, H_Ar_), 7.04–6.87 (m, 2H, H_Ar_), 3.78 (s, 3H, CH**_3_**). ^13^C NMR (101 MHz, DMSO-*d*_6_) δ 163.3, 162.7, 160.0, 155.4, 154.9, 145.4, 132.8, 121.8, 114.4, 99.6, 55.6. HRMS-ESI (*m/z*): calcd for C_13_H_11_N_7_O_3_Ag [M + Ag]: 419.9969, 421.9966; found: 419.9987, 421.9984.

##### 4-(3-Nitro-1*H*-1,2,4-triazol-1-yl)-*N*-(p-tolyl)pyrimidin-2-amine (2**m**)

A glass screw-capped vial containing a magnetic stir bar was charged with 2-chloro-4-(3-nitro-1*H*-1,2,4-triazol-1-yl)pyrimidine (**5**, 0.44 mmol, 100 mg), *p*-toluidine (1.32 mmol, 142 mg) and DMSO (2 mL). The reaction mixture was stirred at 100 °C for 3 h. The reaction mixture was poured into water (10 mL) and cooled in a refrigerator. The resulting precipitate was filtered off and additionally crystallized from ethanol to afford the title compound. Orange solid, yield 62%; mp 229–231 °C (decomp.). ^1^H NMR (400 MHz, DMSO-*d*_6_) δ 9.78 (s, 1H, NH), 9.47 (s, 1H, triazole), 8.73 (d, *J* = 5.2 Hz, 1H, H_Ar_), 7.68–7.59 (m, 2H, H_Ar_), 7.26 (d, *J* = 5.2 Hz, 1H, H_Ar_), 7.17 (d, *J* = 8.1 Hz, 2H, H_Ar_), 2.31 (s, 3H, CH**_3_**). ^13^C NMR (101 MHz, DMSO) δ 163.3, 162.6, 159.9, 154.9, 145.4, 137.2, 131.8, 129.6, 120.1, 99.9, 20.9. HRMS-ESI (*m/z*): calcd for C**_13_**H**_11_**N**_7_**NaO**_2_** [M + Na]: 320.0866; found: 320.0876.

### 4.3. Evaluation of Antimycobacterial Activity

*Mycobacterium tuberculosis* H_37_Rv strain was obtained from the Federal Scientific Center for Expertise of Medical Products (RF Ministry of Health Care) on 7 August 2013 (origin: Prague, Institute of Hygiene and Epidemiology, 1976). The lyophilized strain was seeded on Löwenstein–Jensen growth medium. The 21-days culture was suspended in physiological solution containing glycerol (15%) and transferred into cryotubes to be stored at −80 °C. Prior to the experiment (3 weeks), the culture was brought to ambient temperature and re-seeded into Löwenstein–Jensen growth medium. Thus, the second generation of the original *M. tuberculosis* culture was used in this study.

REMA (resazurin microtitre plate assay) [23] was used to determine the activity of the synthesized compounds. A 3-week *M. tuberculosis* culture was transferred into a dry, sterile tube containing 8–9 3 mm glass beads. The tube was placed on a Vortex shaker for 30–40 s, followed by addition of Middlebrook 7H9 Broth (5 mL; Becton Dickinson, Franklin Lakes, NJ, USA; catalogue No. 271310). The turbidity of the bacterial suspension was adjusted to 1.0 McFarland units (corresponding to approximately 3 × 10^8^ bacteria/mL) and diluted 20-fold with Middlebrook 7H9 Broth containing OADC enrichment (Becton Dickinson, Franklin Lakes, NJ, USA; catalogue No. 245116). The same culture medium was used to prepare the 1:100 *M. tuberculosis* (1% population) control. The concentration of stock solutions of the compounds in DMSO (10 mg/mL) was adjusted to 800 μg/mL by dilution with Middlebrook 7H9 Broth (containing OADC enrichment). A 200 µL aliquot of this solution was introduced into the 2nd row of a 96-well microtitre plate. This row was used to perform 2-fold serial dilutions using and 8-channel pipette to obtain final concentrations of 1.6, 3.1, 6.2, 12.5, 25, 50, 100, 200 and 400 μg/mL of the compound in rows 2–9 (accounting for 100 μL of bacterial suspension introduced for testing). Row 10—MTb suspension control, row 11—same culture diluted 10-fold (the 1% control). Row 12 was used as a blank control for optical density reading (200 μL of the grown medium). The bacterial suspension (100 μL) was introduced into each well except rows 11 (1% population control) and 12 (blank culture medium), to the total volume of 200 μL in each well. The plates were incubated at 35 °C for 7 days, followed by addition of 0.01% aqueous solution (30 μL) of resazurin (Sigma, St. Louis, MO, USA; product No. R7017) in each well and the further incubation for 18 h at 35 °C. The fluorescence reading was performed using the FLUOstar Optima plate reader operating at λ_ex_ = 520 nm and λ_em_ = 590 nm. By comparing the mean values (± SD at *p* = 0.05) of fluorescence in the control wells (row 12, blank and row 11, 1% control) and the wells containing the compound tested, the bacterial viability was determined.

### 4.4. Screening against ESKAPE Pathogens

Susceptibility testing of the following microorganisms: Enterococcus faecium, Staphylococcus aureus, Klebsiella pneumoniae, Acinetobacter baumannii, Pseudomonas aeruginosa and Enterobacter aerogenes, to compounds **1a-s**, **2a-s** and **4** as well as ciprofloxacin (positive control) was performed using the conventional Kirby–Bauer disk diffusion test [24] under the Standard Operating Procedure of The European Committee on Antimicrobial Susceptibility Testing (EUCAST) [25]. Disks containing 5 mg of ciprofloxacin were used. Solutions of compounds **1a-s**, **2a-s** and **4** in dimethyl sulfoxide (1 mg/10 mL) were prepared and diluted to a volume of 1 mL with deionized water. The resulting solutions aliquots (5 mL) were added to a Petri dish containing Muller-Hilton agar inoculated with a bacterial suspension (McFarland OD ¼ 0.5). After drying of the compound solution, the Petri dish was incubated at 37 °C for 18 h. By measuring the bacterial growth inhibition zone diameter around the disc with ciprofloxacin or the compounds’ dried solution circular spot, the susceptibility to a drug was assessed. Additionally, minimum inhibitory concentrations (MIC, μg/mL) were determined using serial broth dilutions [26].

### 4.5. Cyclic Voltammetry

Cyclic voltammetry studies were performed with the potential scan rate of 100 mV∙s^−1^ in a sealed three-electrode cell at 25 °C under argon atmosphere. A flow of argon (extra-purity grade, 99.998%) was bubbled through the solutions to remove dissolved oxygen. Electrochemical measurements were performed in 10^−3^ mol∙dm^−3^ solutions of the tested compounds in 0.1 M TBAClO_4_/DMF. A typical cell used consisted of a working electrode (glassy carbon disk, 0.07 cm^2^), a counter electrode (platinum plate, 1 cm^2^) and a reference electrode (BAS MF-2062 Ag/0.1 M AgNO**_3_** solution in CH**_3_**CN, calibrated by 10^−3^ mol ∙dm^−3^ ferrocene external standard as a pseudo-reference electrode to comprise −188 mV referred to Fc/Fc^+^). All potentials are quoted versus the above reference electrode.

## Figures and Tables

**Table 1:**
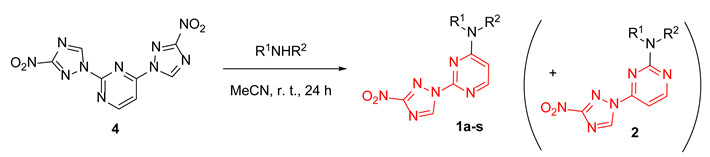
Synthesis of 2-(3-nitro-1,2,4-triazol-1-yl)pyrimidines **1a-s**.

Entry	Compound	Product	Ratio 1:2	Isolated Yield of 1 (%)
1	**1a**	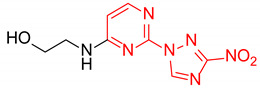	4.6:1	65
2	**1b**	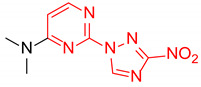	2.5:1	48
3	**1c**	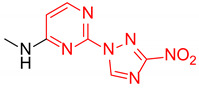	3.2:1	61
4	**1d**	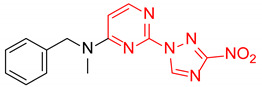	4:1	68
5	**1e**	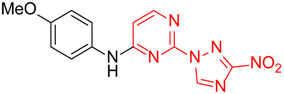	11.2:1 ^a^	45
6	**1f**	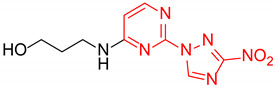	4.6:1	35
7	**1g**	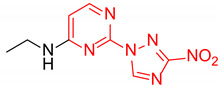	2.2:1	46
8	**1h**	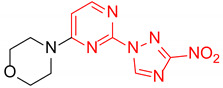	3.5:1	38
9	**1i**	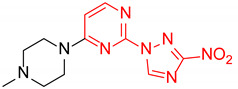	22:1	66
10	**1j**	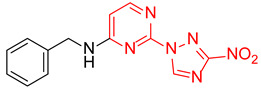	3.5:1	39
11	**1k**	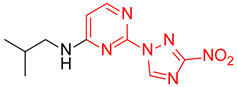	1.4:1	32
12	**1l**	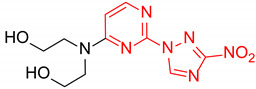	1.2:1	28
13	**1m**	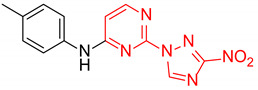	1.6:1 ^a^	35
14	**1n**	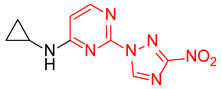	3.5:1	47
15	**1o**	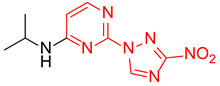	2.4:1	43
16	**1p**	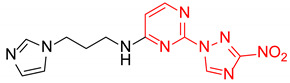	7.2:1	66
17	**1q**	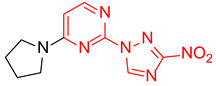	4:1	44
18	**1r**	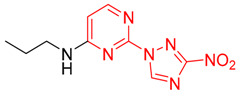	- ^b^	61
19	**1s**	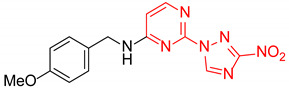	- ^b^	19

^a^ Reactions were performed at 100 °C in DMSO over 24–48 h. ^b^ No regioisomer was formed.

**Table 2:**
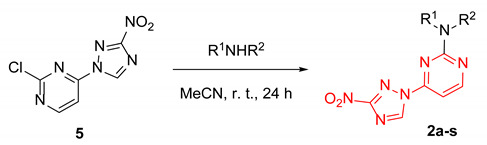
Synthesis of 4-(3-nitro-1,2,4-triazol-1-yl)pyrimidines **2a-s**.

Entry	Compound	Product	Yield (%)
1	**2a**	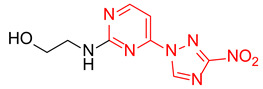	39
2	**2b**	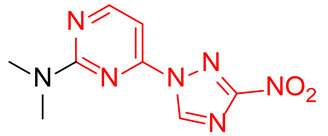	54
3	**2c**	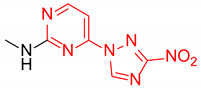	62
4	**2d**	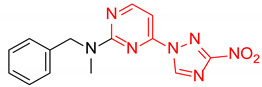	23
5	**2e**	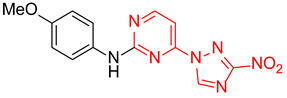	41 ^a^
6	**2f**	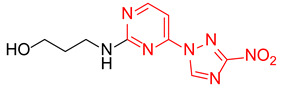	58
7	**2g**	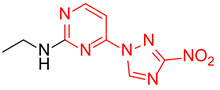	75
8	**2h**	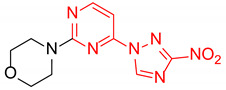	83
9	**2i**	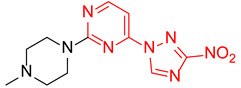	76
10	**2j**	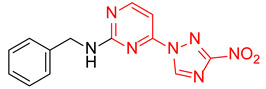	81
11	**2k**	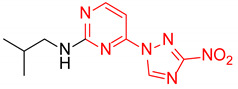	44
12	**2l**	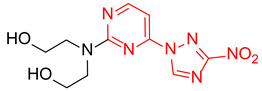	71
13	**2m**	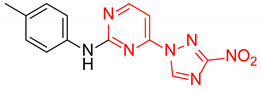	62 ^a^
14	**2n**	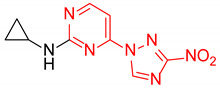	49
15	**2o**	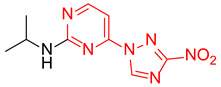	59
16	**2p**	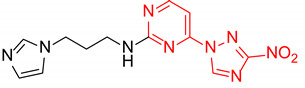	26
17	**2q**	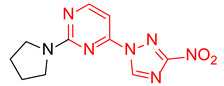	40
18	**2r**	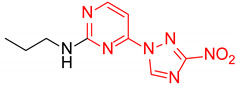	49
19	**2s**	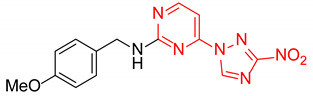	46

^a^ Reactions were performed at 100 °C in DMSO over 24–48 h.

**Table 3:**
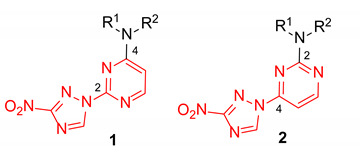
Antibacterial activity (minimal inhibitory concentration, MIC) of compounds **1a-s**, **2a-s**, **4** and ciprofloxacin (positive control) against the ESKAPE panel of pathogens (E1 = *Enterococcus faecium* (G+), S = *Staphylococcus aureus* (G+), K = *Klebsiella pneumonia* (G-), A = *Acinetobacter baumannii* (G-), P = *Pseudomonas aeruginosa* (G-), E2 = *Enterobacter aerogenes* (G-)).

Entry	Compound	R^1^NR^2^ Side Chain	MIC, μg/mL					
			E1	S	K	A	P	E2
1	**1a**	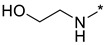	16	>100	>100	16	>100	>100
2	**2a**		8	63	8	>100	>100	63
3	**1b**	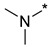	8	>100	>100	4	>100	>100
4	**2b**		8	>100	2	2	>100	8
5	**1c**	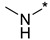	16	>100	>100	4	>100	>100
6	**2c**		63	>100	>100	8	>100	>100
7	**1d**	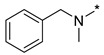	8	16	32	>100	>100	63
8	**2d**		8	63	16	>100	>100	63
9	**1e**	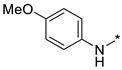	4	>100	63	>100	>100	>100
10	**2e**		8	8	>100	4	>100	63
11	**1f**	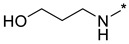	4	>100	>100	8	>100	>100
12	**2f**		8	63	8	32	>100	16
13	**1g**	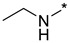	4	>100	>100	32	>100	>100
14	**2g**		32	>100	>100	8	>100	63
15	**1h**	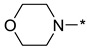	16	>100	>100	32	>100	>100
16	**2h**		4	16	>100	63	>100	>100
17	**1i**	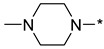	2	4	32	32	>100	32
18	**2i**		2	>100	>100	>100	>100	>100
19	**1j**	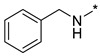	4	>100	>100	4	>100	>100
20	**2j**		8	>100	>100	63	>100	>100
21	**1k**	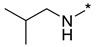	2	63	>100	>100	>100	>100
22	**2k**		>100	>100	>100	4	>100	>100
23	**1l**	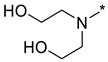	63	>100	>100	4	>100	>100
24	**2l**		16	16	>100	8	>100	>100
25	**1m**	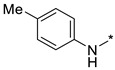	8	8	32	2	>100	16
26	**2m**		16	>100	>100	8	>100	>100
27	**1n**	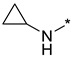	>100	>100	>100	>100	>100	>100
28	**2n**		4	32	>100	63	>100	>100
29	**1o**	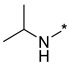	2	4	16	32	>100	32
30	**2o**		>100	>100	>100	8	>100	>100
31	**1p**	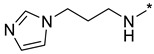	32	>100	>100	8	>100	>100
32	**2p**		4	>100	>100	8	>100	>100
33	**1q**	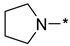	16	>100	>100	4	>100	>100
34	**2q**		32	63	>100	16	>100	>100
35	**1r**	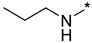	16	>100	>100	8	>100	63
36	**2r**		16	63	>100	>100	>100	>100
37	**1s**	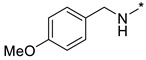	16	63	63	>100	>100	>100
38	**2s**		2	>100	>100	4	>100	>100
39	**4**	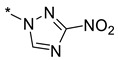	32	>100	>100	>100	>100	>100
40	ciprofloxacin		0.3	1.25	0.6	2.5	0.6	1.25

* Attachment to position 4 in triazolopyrimidine scaffold (compounds **1**) or position 2 (compounds **2**).

**Table 4:** Reduction potentials (E_1/2_, V) of selected compounds **1** and **2** and their MIC values (μg/mL) observed against *E. faecium*.

Entry	Compound	E_1/2_ (V vs. Ag/Ag^+^)	E_1/2_ (V vs. Fc/Fc^+^)	*E. faecium* MIC (μg/mL)
1	**1i**	−1.253	−1.439	2
2	**1j**	−1.262	−1.447	4
3	**1m**	−1.246	−1.432	8
4	**1n**	−1.255	−1.441	>100
5	**2b**	−1.195	1.381	8
6	**2e**	−1.178	−1.364	8
7	**2i**	−1.192	−1.378	2
8	**2r**	−1.196	−1.382	16
9	ferrocene	0.186	0.000	–

## Supplementary Materials

The following are available online at https://www.mdpi.com/2079-6382/9/10/666/s1, Table S1: Crystal data and structure refinement for 1e, 5; Table S2: Crystal data and structure refinement for **2o**, **2k**; Table S3: Crystal data and structure refinement for **2n**, **2h**; Copies of ^1^H and ^13^C NMR spectra.
