# Supplementary material for: Mutually Isomeric 2- and 4-(3-Nitro-1,2,4-triazol-1-yl)pyrimidines Inspired by an Antimycobacterial Screening Hit: Synthesis and Biological Activity against the ESKAPE Panel of Pathogens
Source: Antibiotics (Basel). 2020 Oct 1;9(10):666. doi: 10.3390/antibiotics9100666 (PMC7601023; doi:10.3390/antibiotics9100666)

Supporting information for

Mutually isomeric 2- and 4-(3-nitro-1,2,4-triazol-1-yl)pyrimidines inspired by an antimycobacterial screening hit: synthesis and biological activity against the ESKAPE panel of pathogens

Sergey Chuprun,[a] Dmitry Dar'in,[a] Elizaveta Rogacheva,[b] Liudmila Kraeva,[b] Oleg Levin,[a] Olga Manicheva,[c] Marine Dogonadze,[c] Tatiana Vinogradova,[c] Olga Bakulina,[a] and Mikhail Krasavin[a,d]*

[a]Saint Petersburg State University, Saint Petersburg 199034 Russian Federation

[b]Pasteur Institute of Epidemiology and Microbiology, 14 Mira Street, Saint Petersburg 197101 Russian Federation

[c]Saint Petersburg Research Institute of Phthisiopulmonology, 2-4 Ligovsky Prospekt, Saint Petersburg, 191036, Russian Federation

[d]Immanuel Kant Baltic Federal University, Kaliningrad 236016 Russian Federation

*Corresponding author. Tel.: +7 931 3617872; fax: +7 812 428 6939. E-mail address: m.krasavin@spbu.ru (M. Krasavin).

Table of Contents

Crystallographic data for compounds 1e, 2h,k,n,o and 5 ....S2

References S6

Copies of ^{1}H and ^{13}C NMR spectra of compounds 1a-s,2a-s,4 and 5 ....S7

Crystallographic data for compounds 1e, 2h,k,n,o and 5

Suitable crystals of **2o**, **2k**, **2n**, and **5** were studied using Xcalibur, Eos diffractometer (monochromated Mo*Kα* radiation, *λ* = 0.71073 Å). Suitable crystals of **2h** and **1e** were studied using SuperNova, Dual, Cu at zero, Atlas diffractometer (monochromated Cu*Kα* radiation, *λ* = 1.542 Å). In all cases apart from **2n**, the temperature was kept at 100(2) K, whereas in the case of **2n**, the temperature was kept at 200(2) K. In each case the structure has been solved with the ShelXT [1] structure solution program using Intrinsic Phasing and refined with the ShelXL [1] refinement package incorporated in the OLEX2 program package [2] using Least Squares minimization. Empirical absorption correction was applied in CrysAlisPro [3] program complex using spherical harmonics, implemented in SCALE3 ABSPACK scaling algorithm.

Table S1. Crystal data and structure refinement for **1e**, **5**

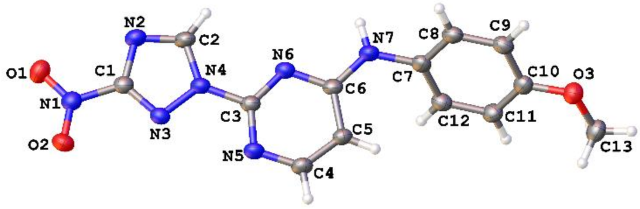

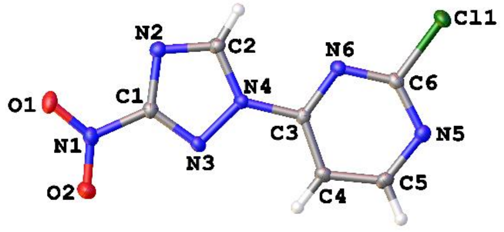

Identification code	**1e**	**5**
Empirical formula	$C_{13}H_{11}N_7O_3$	$C_6H_3ClN_6O_2$
Formula weight	313.29	226.59
Temperature/K	100(2)	100(2)
Crystal system	orthorhombic	monoclinic
Space group	Pbca	$P2_1/c$
a/Å	11.5561(9)	12.8957(3)
b/Å	6.9509(7)	11.5155(3)
c/Å	34.027(4)	11.5580(3)
α/°	90	90
β/°	90	90.988(2)
γ/°	90	90
Volume/Å³	2733.2(5)	1716.11(7)
Z	8	8
ρ_{calc}g/cm³	1.523	1.754
μ/mm⁻¹	0.966	0.434
F(000)	1296.0	912.0
Crystal size/mm³	0.18 × 0.09 × 0.08	0.21 × 0.20 × 0.16
Radiation	Cu Kα (λ = 1.54184)	Mo Kα (λ = 0.7107)
2Θ range for data collection/°	9.252 to 152.326	5.878 to 56.992
Index ranges	–14 ≤ h ≤ 14, –4 ≤ k ≤ 8, –40 ≤ l ≤ 42	–17 ≤ h ≤ 17, –15 ≤ k ≤ 15, –15 ≤ l ≤ 15

Reflections collected	11128	17415
Independent reflections	2821 [R_{int} = 0.0742, R_{sigma} = 0.0454]	4347 [R_{int} = 0.0310, R_{sigma} = 0.0280]
Data/restraints/parameters	2821/0/209	4347/0/271
Goodness-of-fit on F^2	1.040	1.025
Final R indexes [I≥2σ (I)]	R_1 = 0.0706, wR_2 = 0.1827	R_1 = 0.0298, wR_2 = 0.0713
Final R indexes [all data]	R_1 = 0.0840, wR_2 = 0.1941	R_1 = 0.0369, wR_2 = 0.0760
Largest diff. peak/hole / e·Å^{-3}	0.34/-0.31	0.40/-0.28
CCDC	1582454	1582455

Table S2. Crystal data and structure refinement for **2o**, **2k**

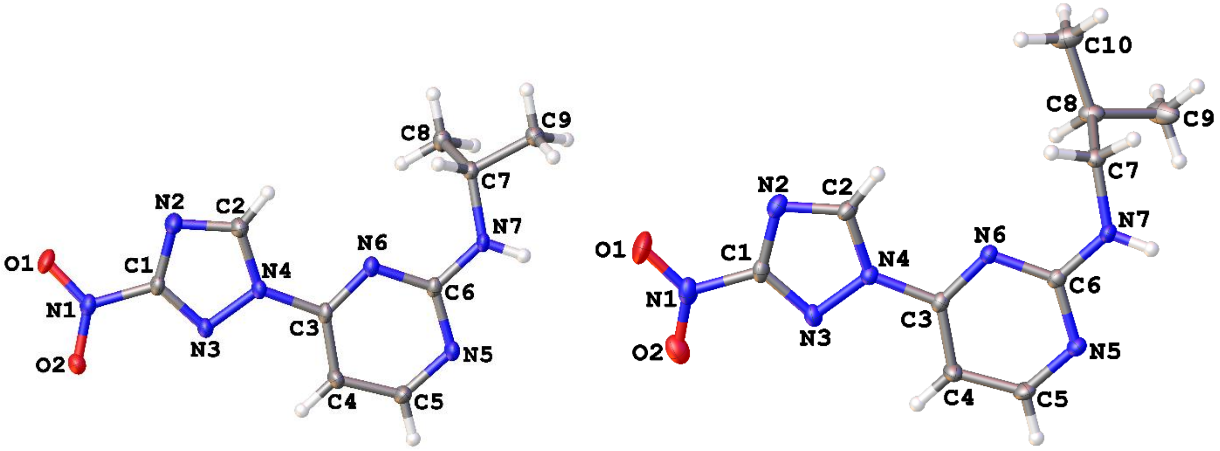

Identification code	**2o**	**2k**
Empirical formula	$C_9H_{11}N_7O_2$	$C_{10}H_{13}N_7O_2$
Formula weight	249.25	263.27
Temperature/K	100(2)	100(2)
Crystal system	monoclinic	monoclinic
Space group	$P2_1/c$	I2/a
a/Å	17.1962(6)	25.0022(15)
b/Å	11.4000(5)	5.6435(3)
c/Å	22.8966(6)	18.6084(7)
α/°	90	90
β/°	94.205(3)	99.093(5)
γ/°	90	90
Volume/Å^3	4476.5(3)	2592.6(2)
Z	16	8
ρ_{calc}g/cm^3	1.479	1.349
μ/mm^{-1}	0.112	0.100
F(000)	2080.0	1104.0
Crystal size/mm^3	0.23 × 0.18 × 0.16	0.20 × 0.11 × 0.08

Radiation	Mo Kα (λ = 0.7107)	Mo Kα (λ = 0.7107)
2Θ range for data collection/°	5.468 to 56.996	5.932 to 56.998
Index ranges	$-23 \le h \le 23$, $-15 \le k \le 9$, $-17 \le l \le 30$	$-33 \le h \le 32$, $-6 \le k \le 7$, $-24 \le l \le 20$
Reflections collected	26644	7489
Independent reflections	11339 [R_{int} = 0.0298, R_{sigma} = 0.0425]	3274 [R_{int} = 0.0270, R_{sigma} = 0.0430]
Data/restraints/parameters	11339/0/657	3274/0/174
Goodness-of-fit on F^2	1.042	1.038
Final R indexes [I≥2σ (I)]	R_1 = 0.0467, wR_2 = 0.1018	R_1 = 0.0450, wR_2 = 0.0955
Final R indexes [all data]	R_1 = 0.0741, wR_2 = 0.1152	R_1 = 0.0696, wR_2 = 0.1052
Largest diff. peak/hole / e·Å^{-3}	0.32/-0.34	0.24/-0.20
CCDC	1582439	1582440

Table S3. Crystal data and structure refinement for **2n**, **2h**

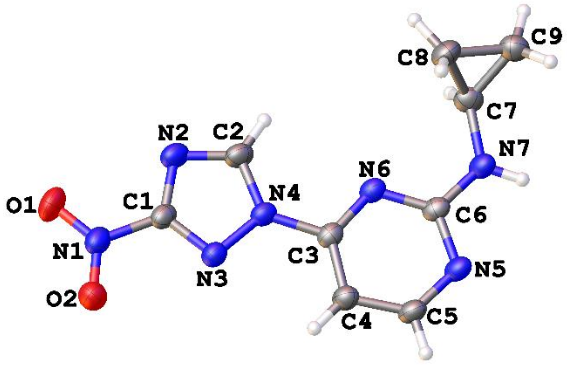

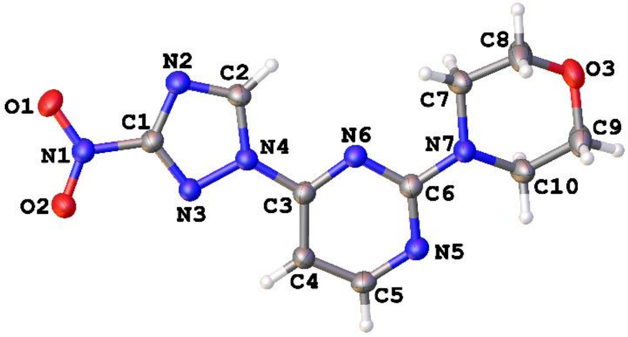

Identification code	**2n**	**2h**
Empirical formula	$C_9H_9N_7O_2$	$C_{10}H_{11}N_7O_3$
Formula weight	247.23	277.26
Temperature/K	200(2)	100(2)
Crystal system	triclinic	triclinic
Space group	P-1	P-1
a/Å	7.8740(6)	8.5133(5)
b/Å	8.5556(6)	8.8429(4)
c/Å	9.2299(6)	16.4034(6)
α/°	77.309(5)	97.654(3)
β/°	72.904(6)	97.519(4)
γ/°	66.902(7)	97.057(4)
Volume/Å^3	542.81(7)	1200.93(10)
Z	2	4
ρ_{calc}g/cm^3	1.513	1.533
μ/mm^{-1}	0.115	1.009
F(000)	256.0	576.0

Crystal size/mm^3	0.18 × 0.11 × 0.07	0.24 × 0.14 × 0.11
Radiation	Mo Kα (λ = 0.7107)	Cu Kα (λ = 1.54184)
2Θ range for data collection/°	5.782 to 56.998	10.198 to 152.69
Index ranges	–10 ≤ h ≤ 10, –11 ≤ k ≤ 11, –12 ≤ l ≤ 12	–10 ≤ h ≤ 10, –10 ≤ k ≤ 10, –18 ≤ l ≤ 20
Reflections collected	9450	10191
Independent reflections	2753 [R_{int} = 0.0252, R_{sigma} = 0.0250]	4905 [R_{int} = 0.0323, R_{sigma} = 0.0393]
Data/restraints/parameters	2753/0/163	4905/40/397
Goodness-of-fit on F^2	1.037	1.051
Final R indexes [I≥2σ (I)]	R_1 = 0.0387, wR_2 = 0.0993	R_1 = 0.0572, wR_2 = 0.1548
Final R indexes [all data]	R_1 = 0.0506, wR_2 = 0.1085	R_1 = 0.0726, wR_2 = 0.1686
Largest diff. peak/hole / e·Å^{-3}	0.22/-0.25	0.41/-0.45
CCDC	1582442	1582450

References

1. Sheldrick, G. M. Acta Crystallographica Section C 2015, 71, 3–8.

2. O. V. Dolomanov, L. J. Bourhis, R. J. Gildea, J. A. K. Howard and H. Puschmann, J. Appl. Cryst., 2009, 42, 339–341.

3. CrysAlisPro, Agilent Technologies Ltd., Version 1.171.136.120 (release 127-106-2012).

Copies of ^1H and ^{13}C NMR spectra of compounds 1a-s, 2a-s, 4 and 5

^1H and ^{13}C NMR of 2,4-bis(3-nitro-1*H*-1,2,4-triazol-1-yl)pyrimidine (4)

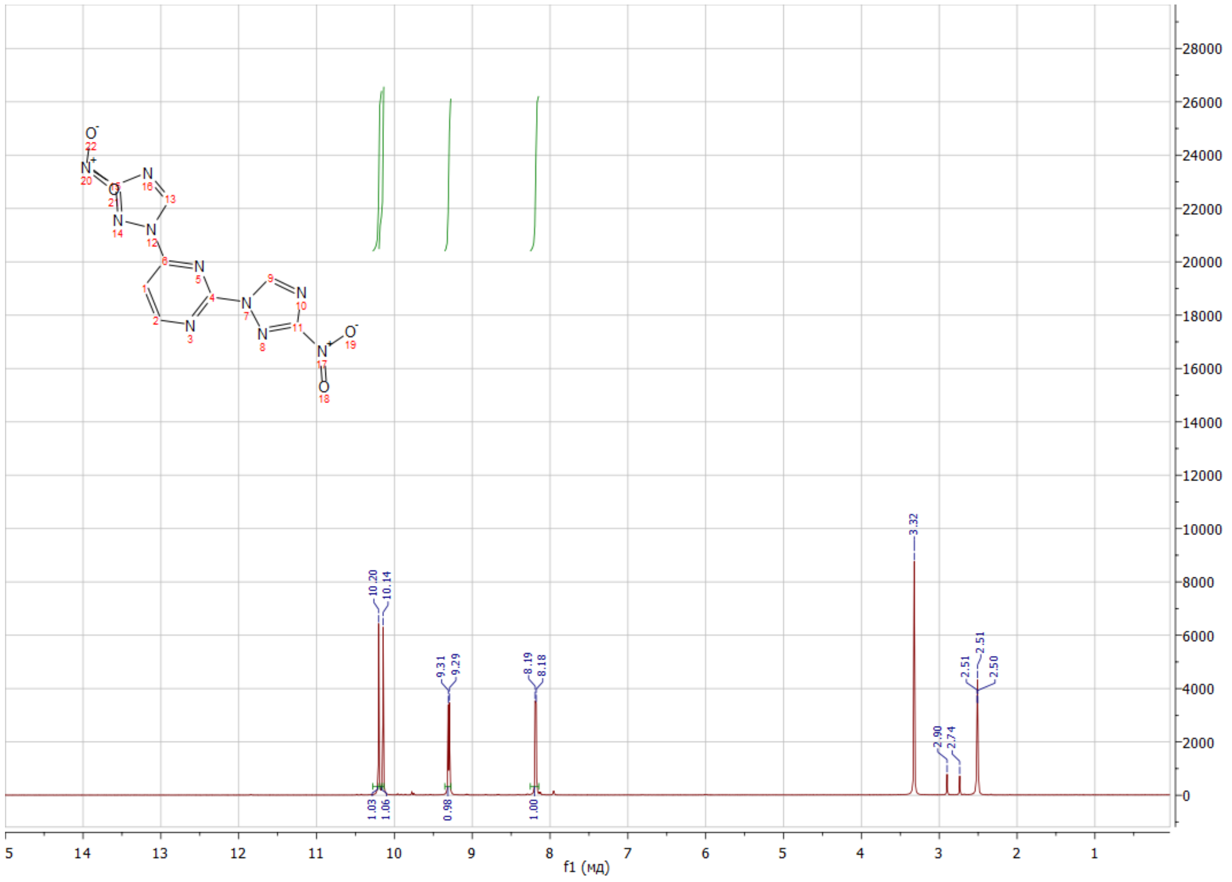

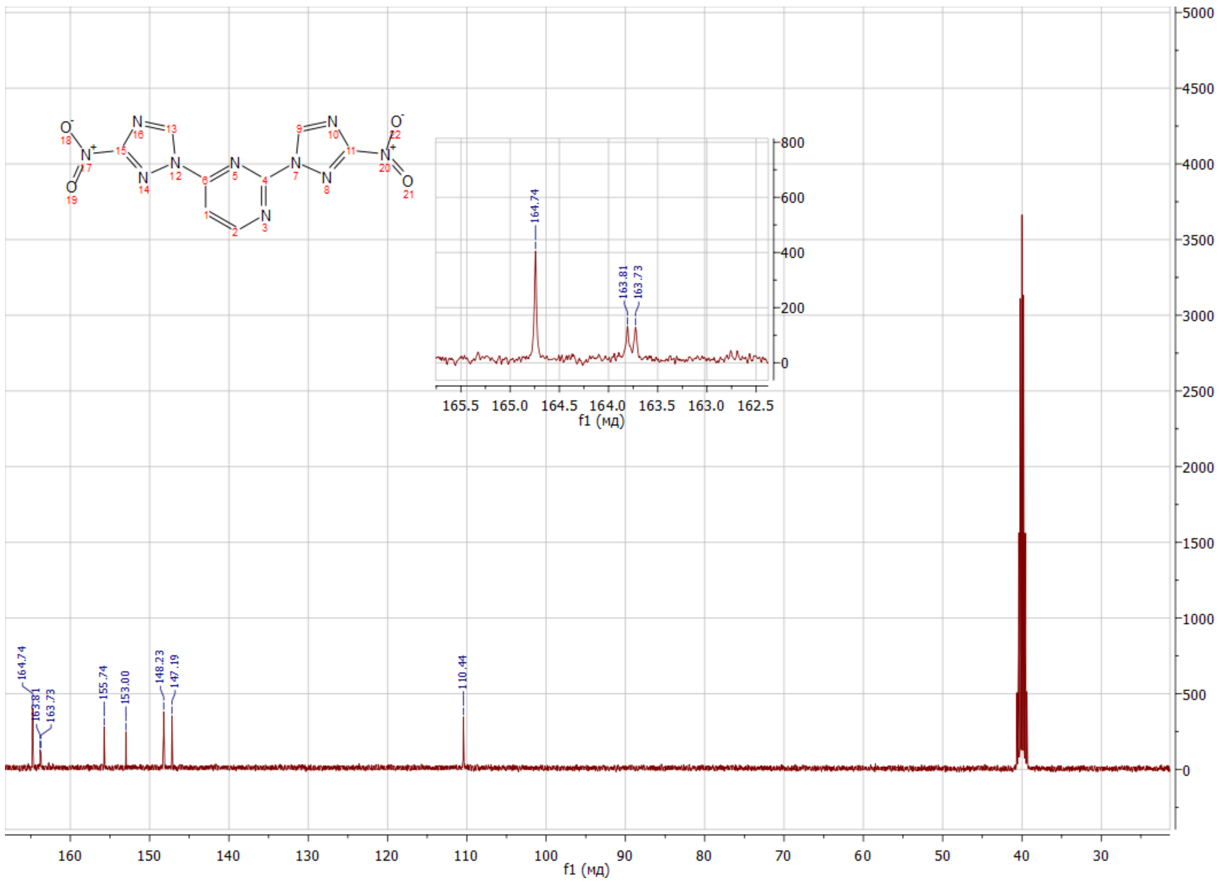

^{1}H and ^{13}C NMR of 2-((2-(3-nitro-1*H*-1,2,4-triazol-1-yl)pyrimidin-4-yl)amino)ethan-1-ol (1a)

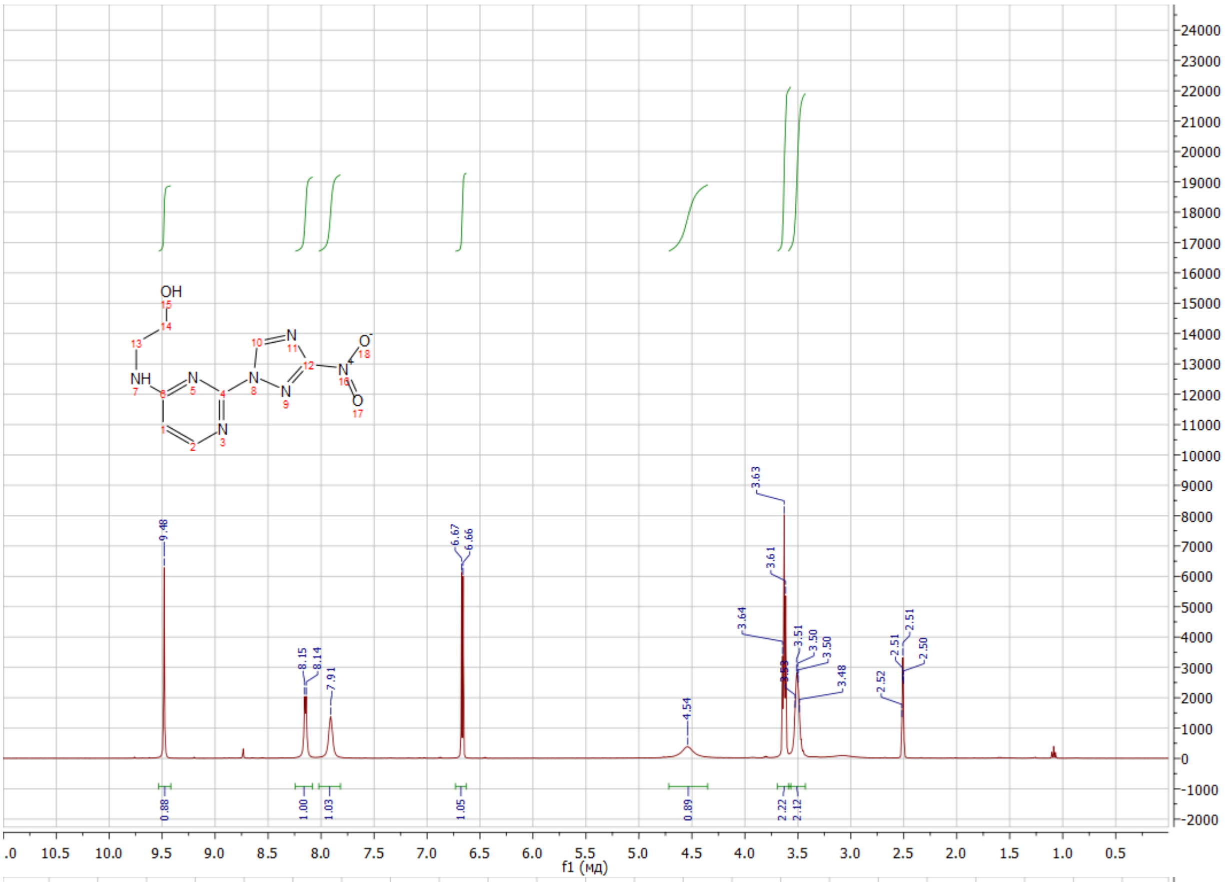

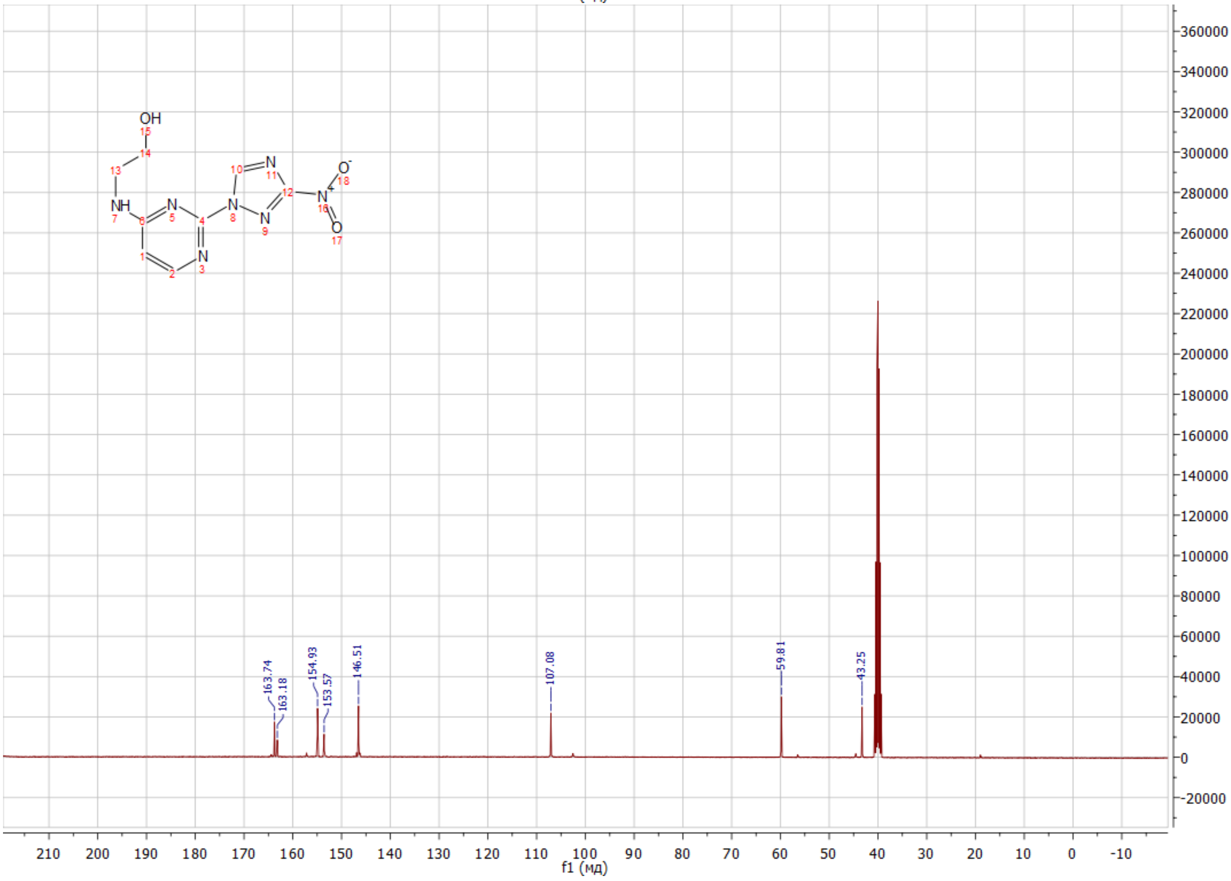

^{1}H and ^{13}C NMR of *N*,*N*-dimethyl-2-(3-nitro-1*H*-1,2,4-triazol-1-yl)pyrimidin-4-amine (1b)

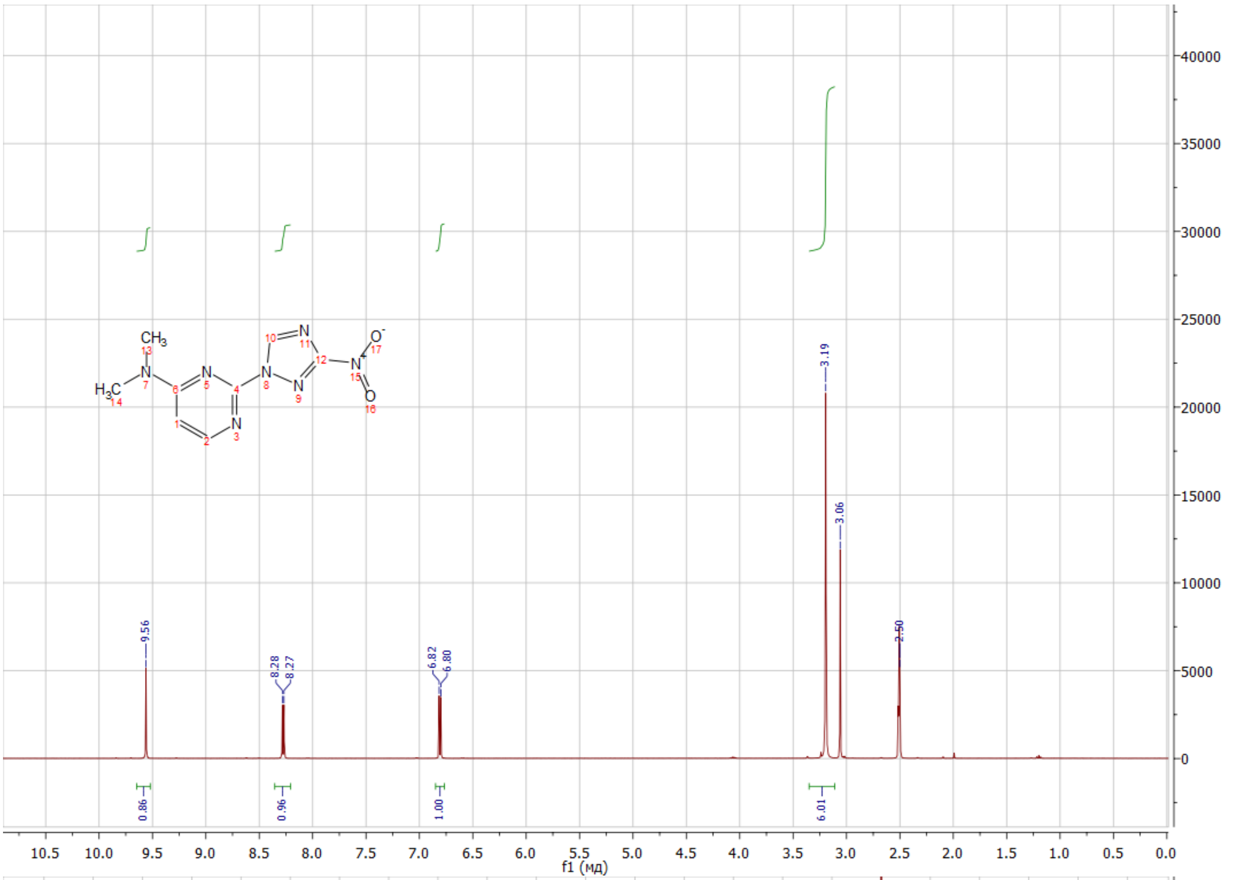

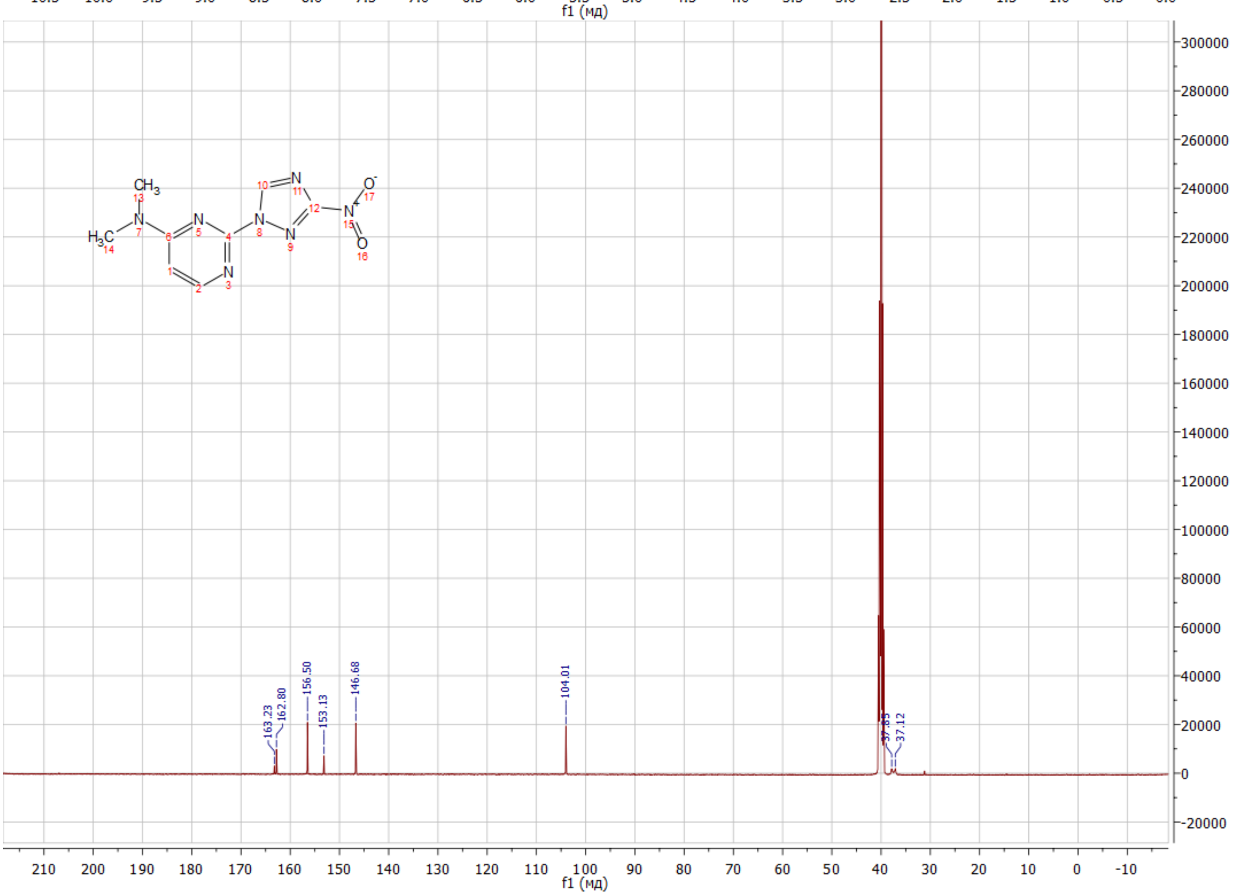

^{1}H and ^{13}C NMR of *N*-methyl-2-(3-nitro-1*H*-1,2,4-triazol-1-yl)pyrimidin-4-amine (1c)

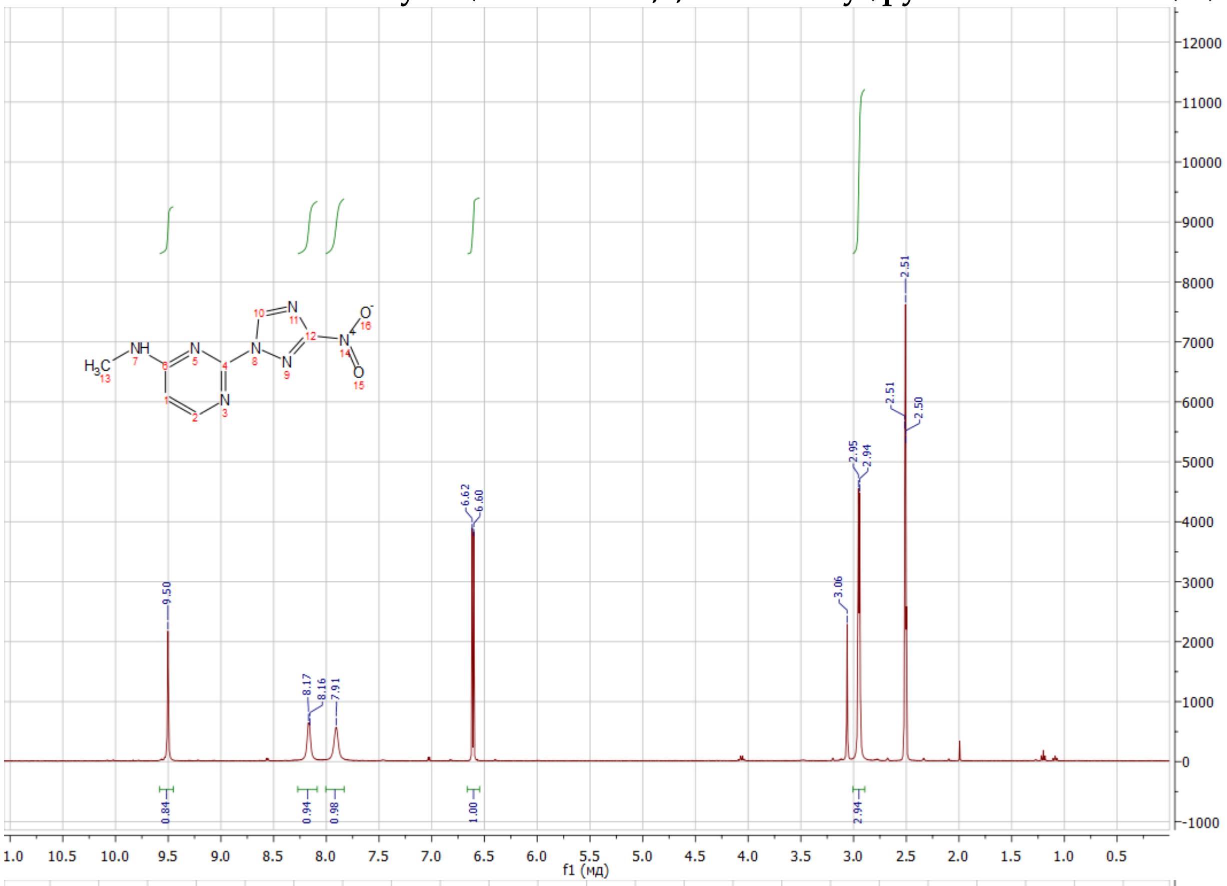

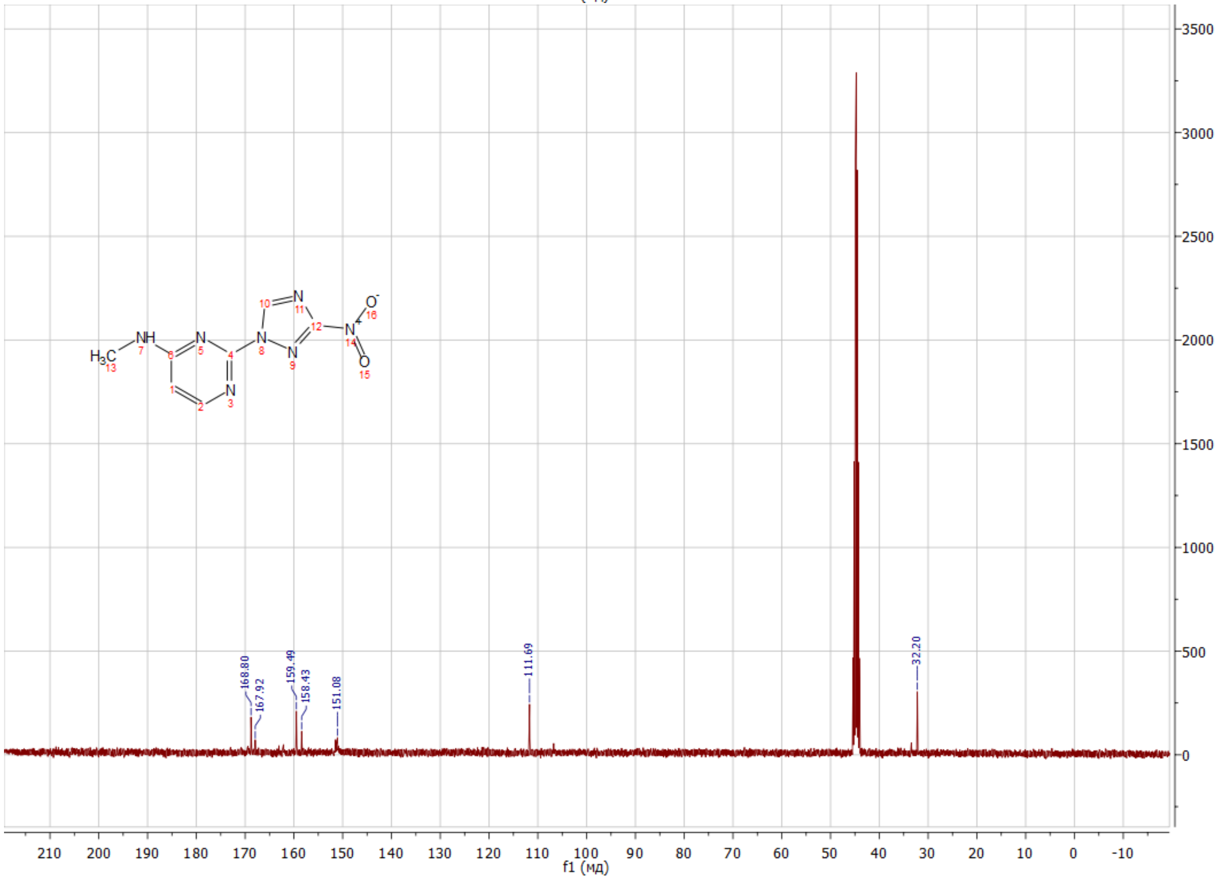

^{1}H and ^{13}C NMR of *N*-benzyl-*N*-methyl-2-(3-nitro-1*H*-1,2,4-triazol-1-yl)pyrimidin-4-amine (1d)

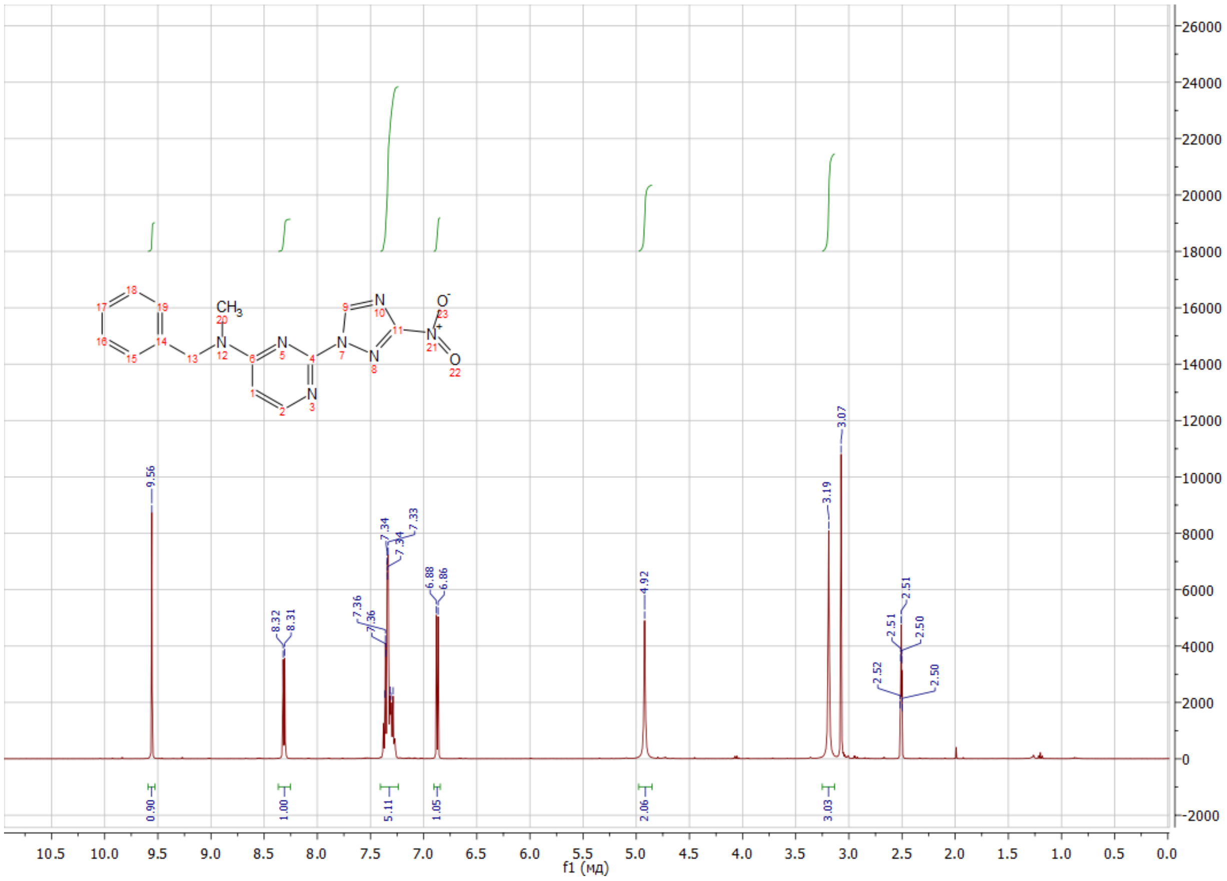

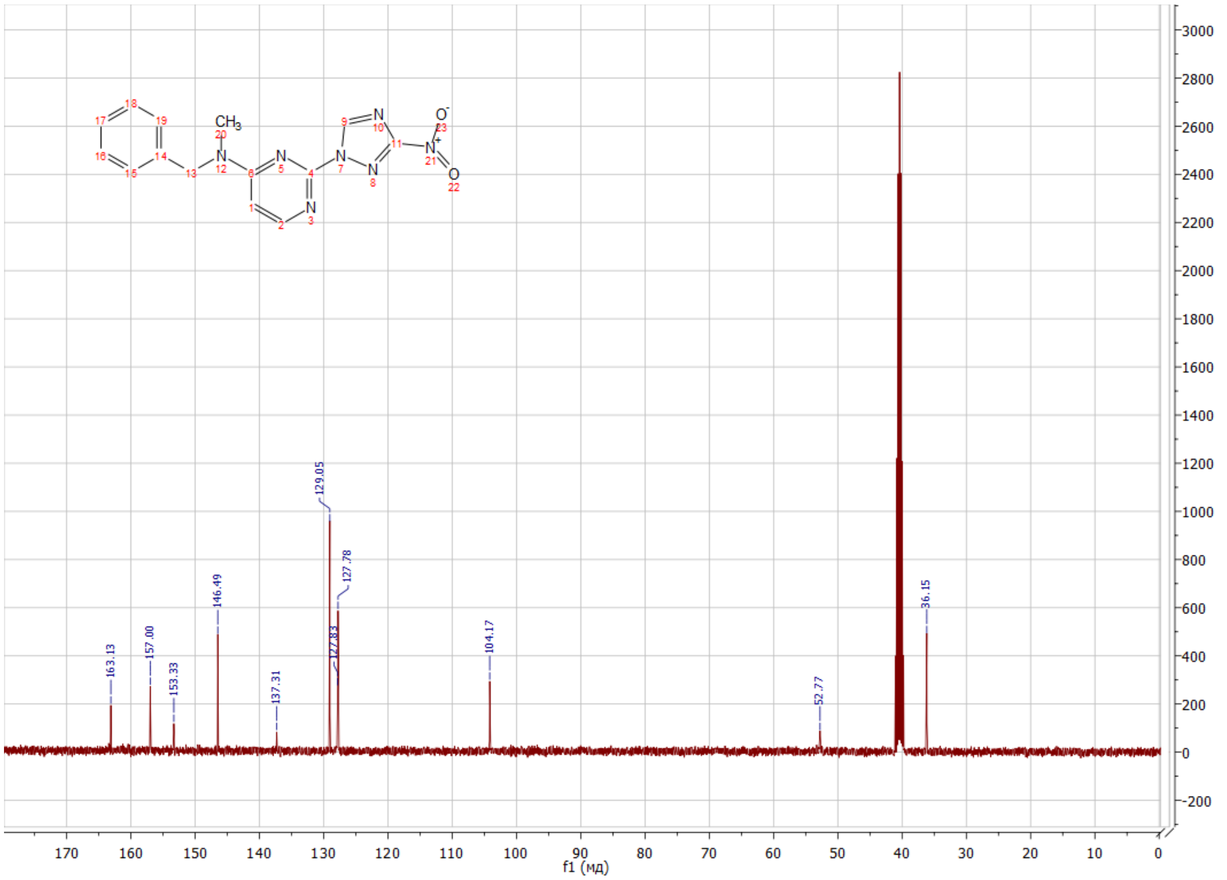

^{1}H and ^{13}C NMR of *N*-(4-methoxyphenyl)-2-(3-nitro-1*H*-1,2,4-triazol-1-yl)pyrimidin-4-amine (1e)

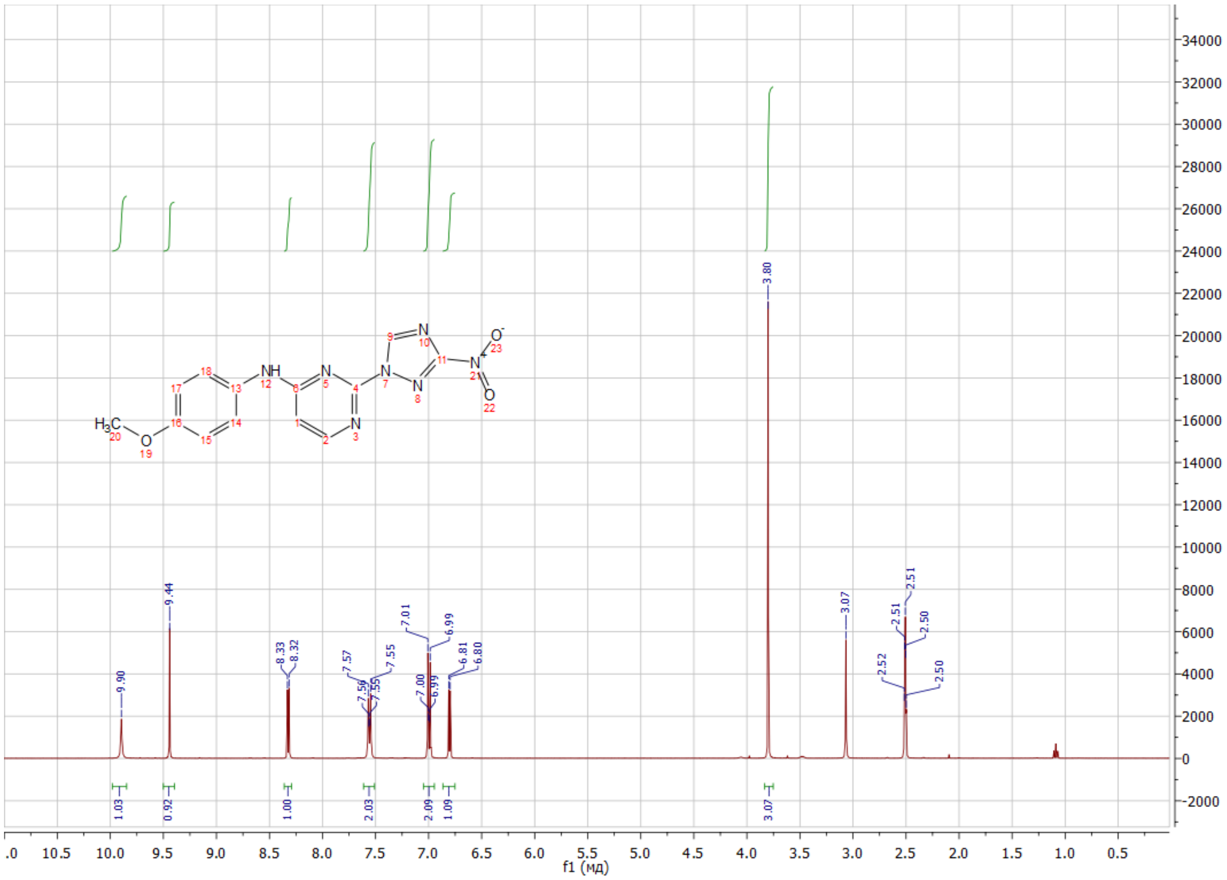

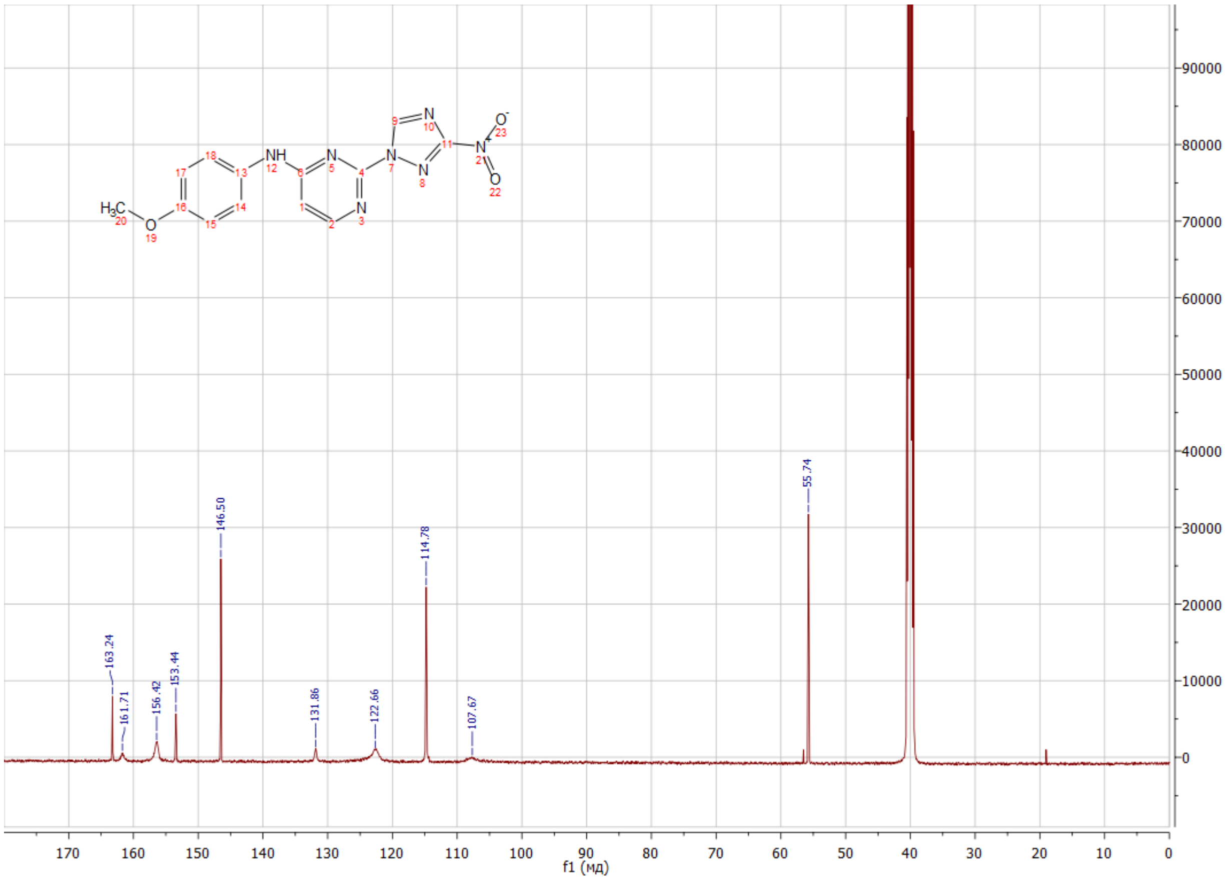

^{1}H and ^{13}C NMR of 3-((2-(3-nitro-1*H*-1,2,4-triazol-1-yl)pyrimidin-4-yl)amino)propan-1-ol (1f)

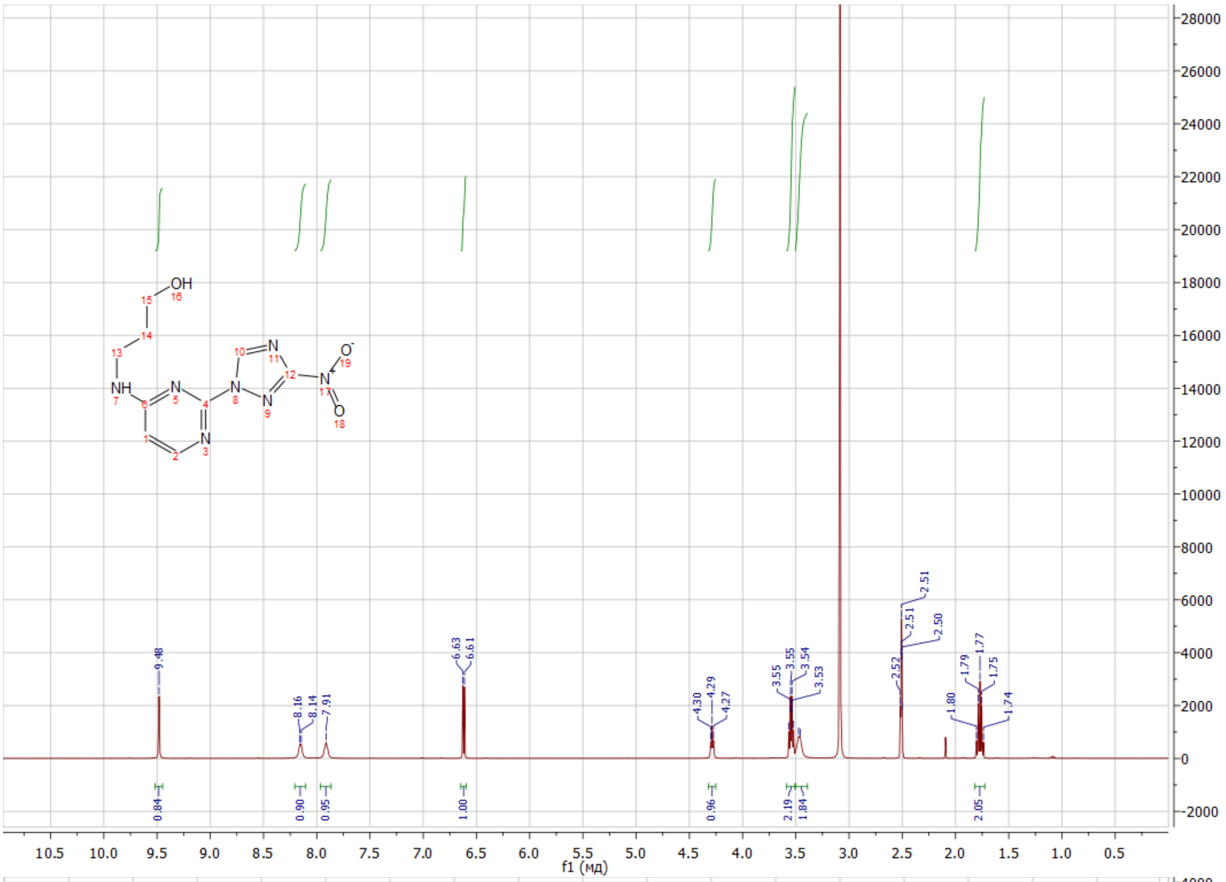

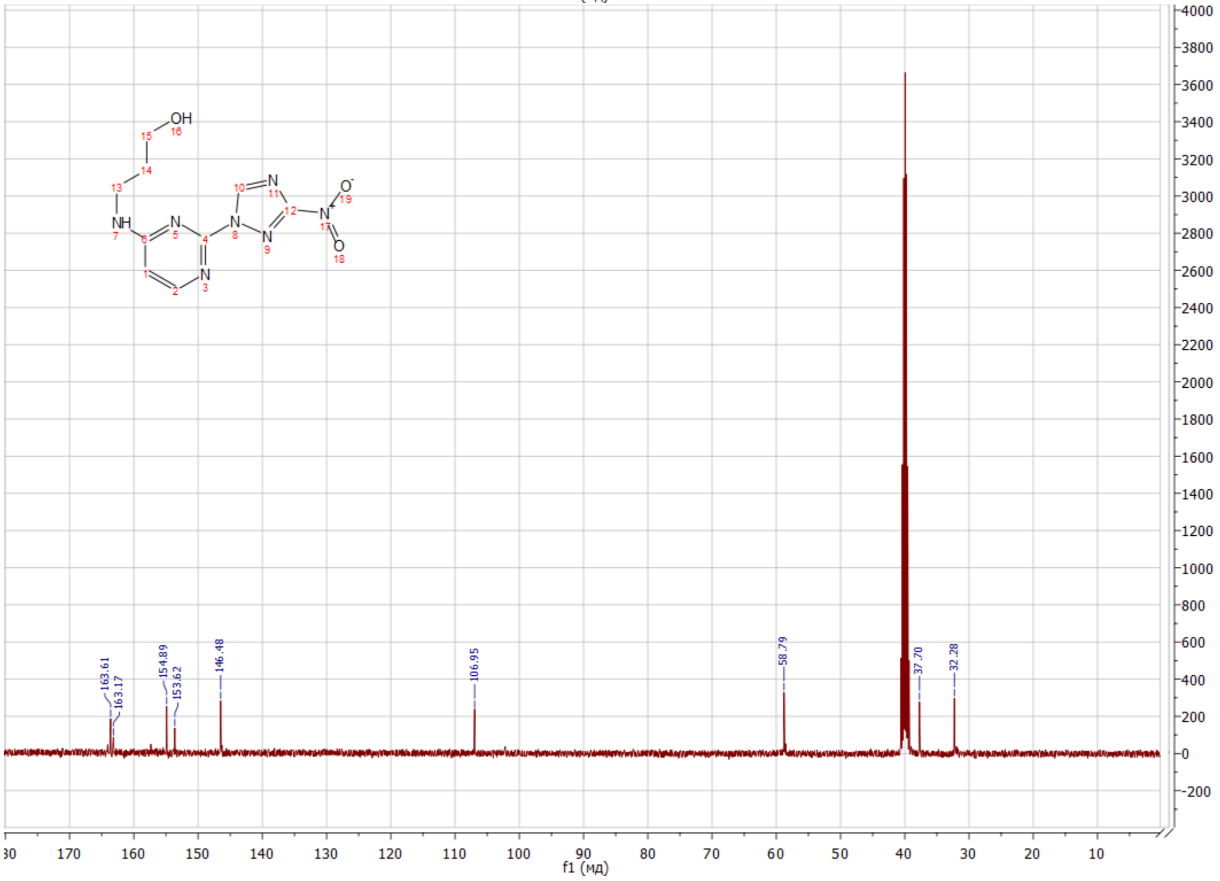

^{1}H and ^{13}C NMR of *N*-ethyl-2-(3-nitro-1*H*-1,2,4-triazol-1-yl)pyrimidin-4-amine (1g)

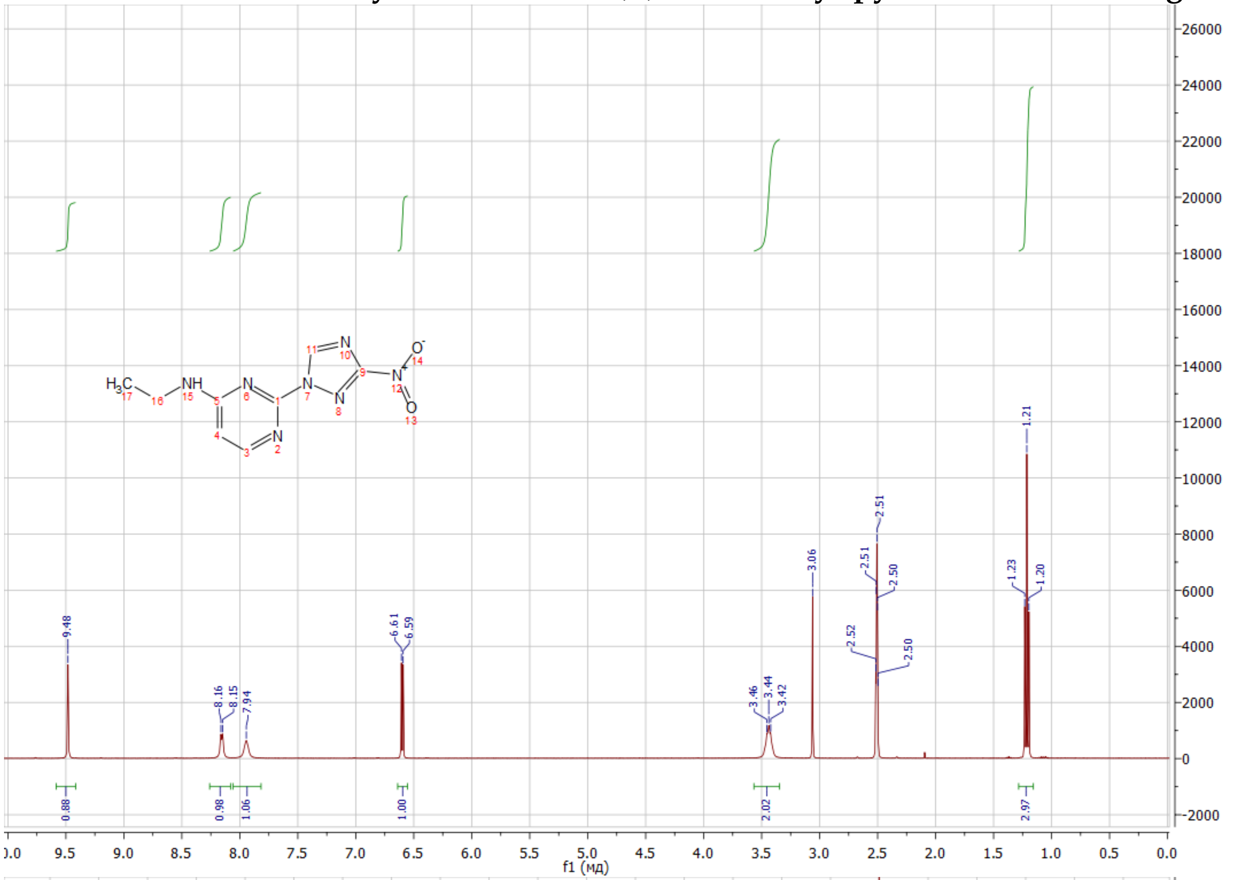

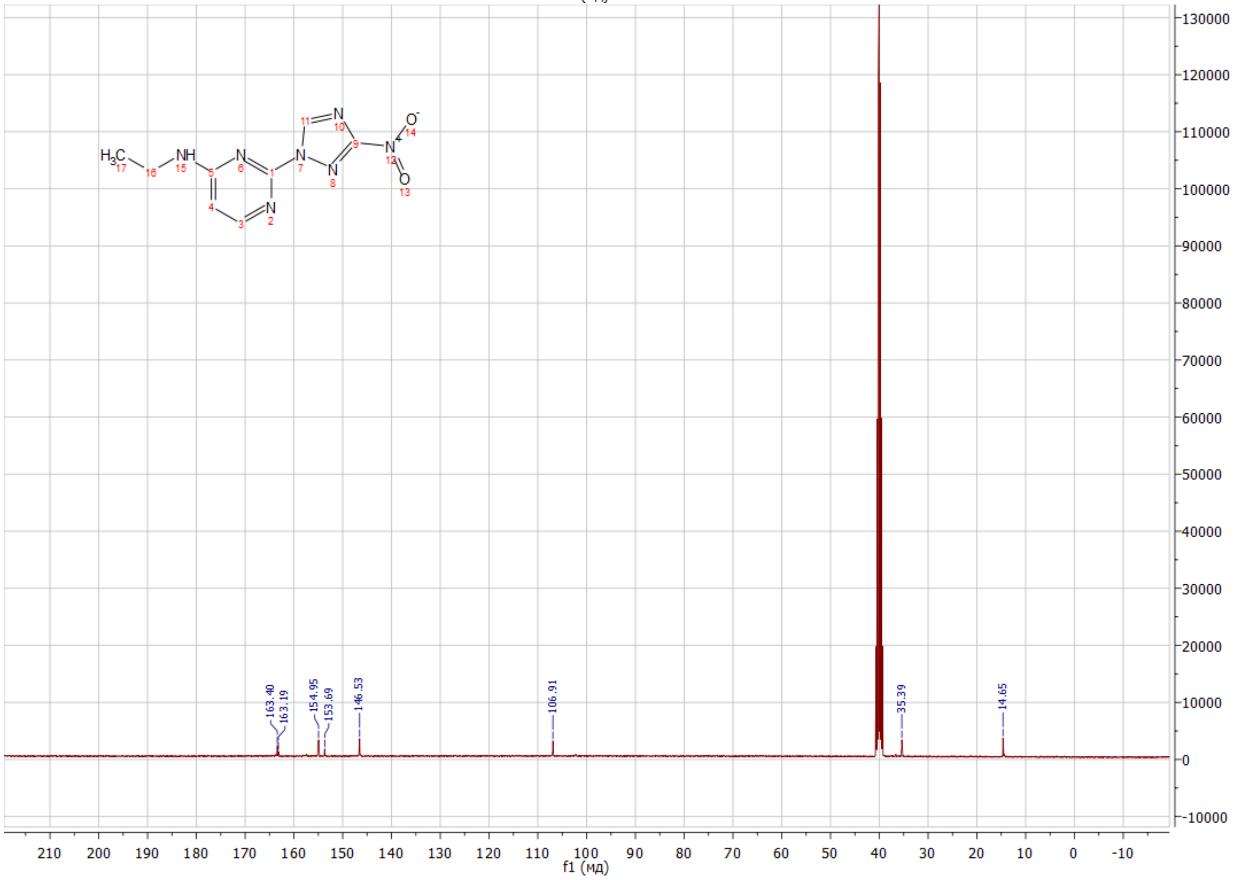

^{1}H and ^{13}C NMR of 4-(2-(3-Nitro-1*H*-1,2,4-triazol-1-yl)pyrimidin-4-yl)morpholine (1h)

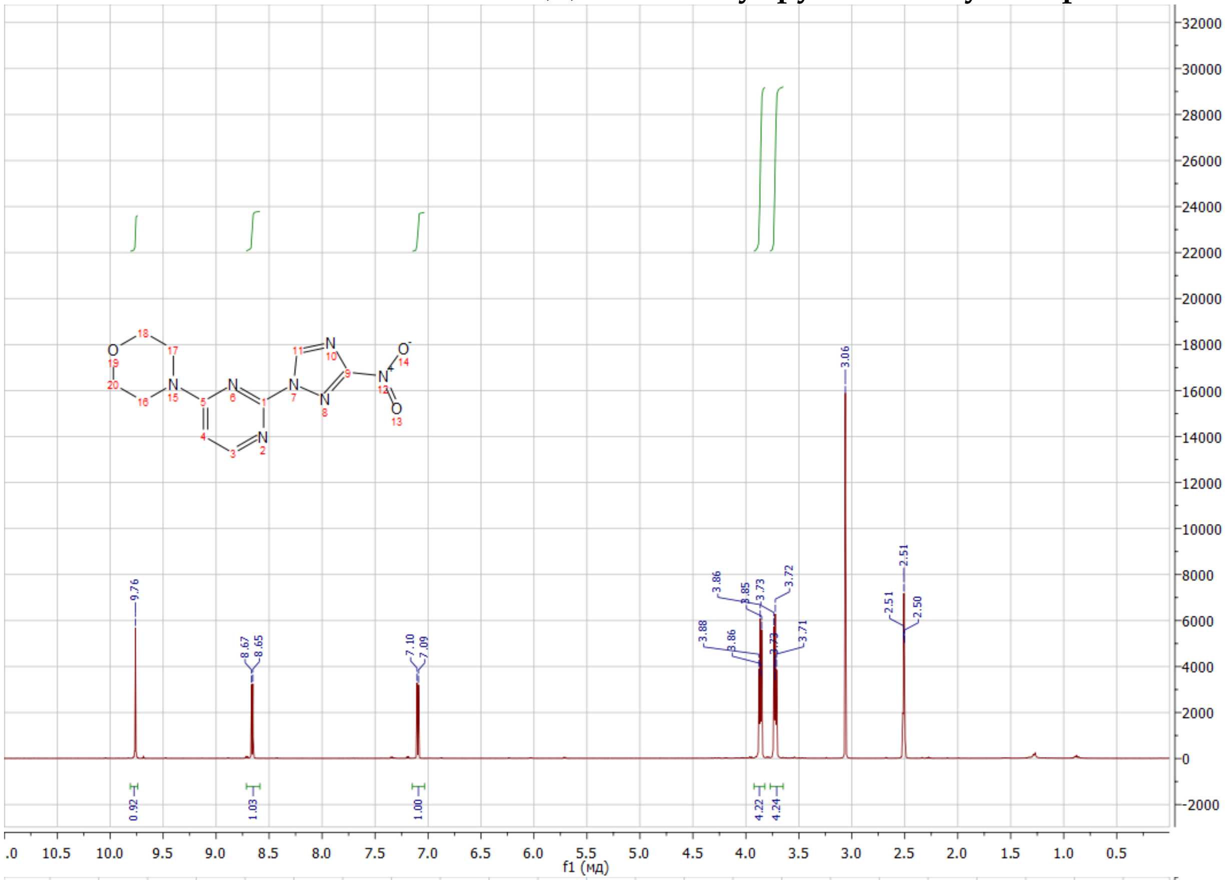

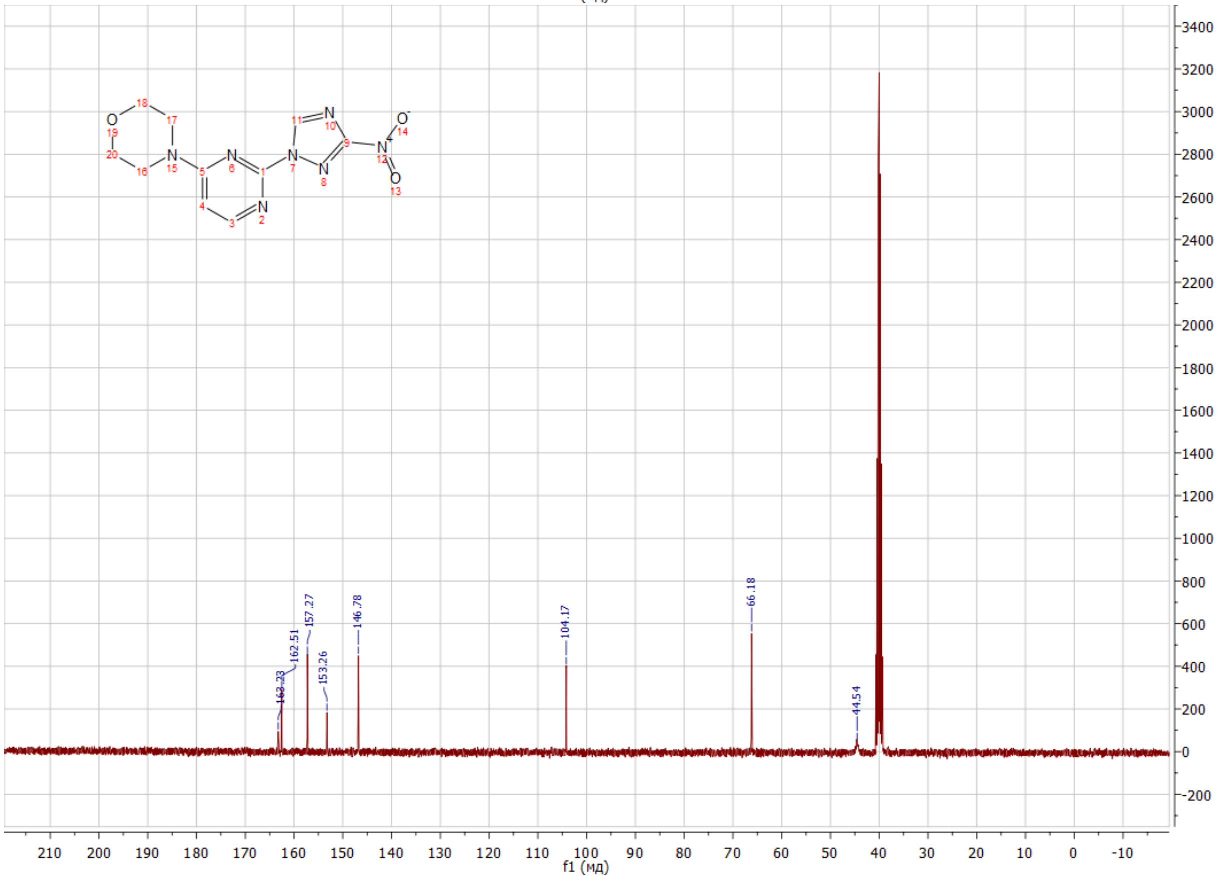

^{1}H and ^{13}C NMR of 4-(4-methylpiperazin-1-yl)-2-(3-nitro-1*H*-1,2,4-triazol-1-yl)pyrimidine (1i)

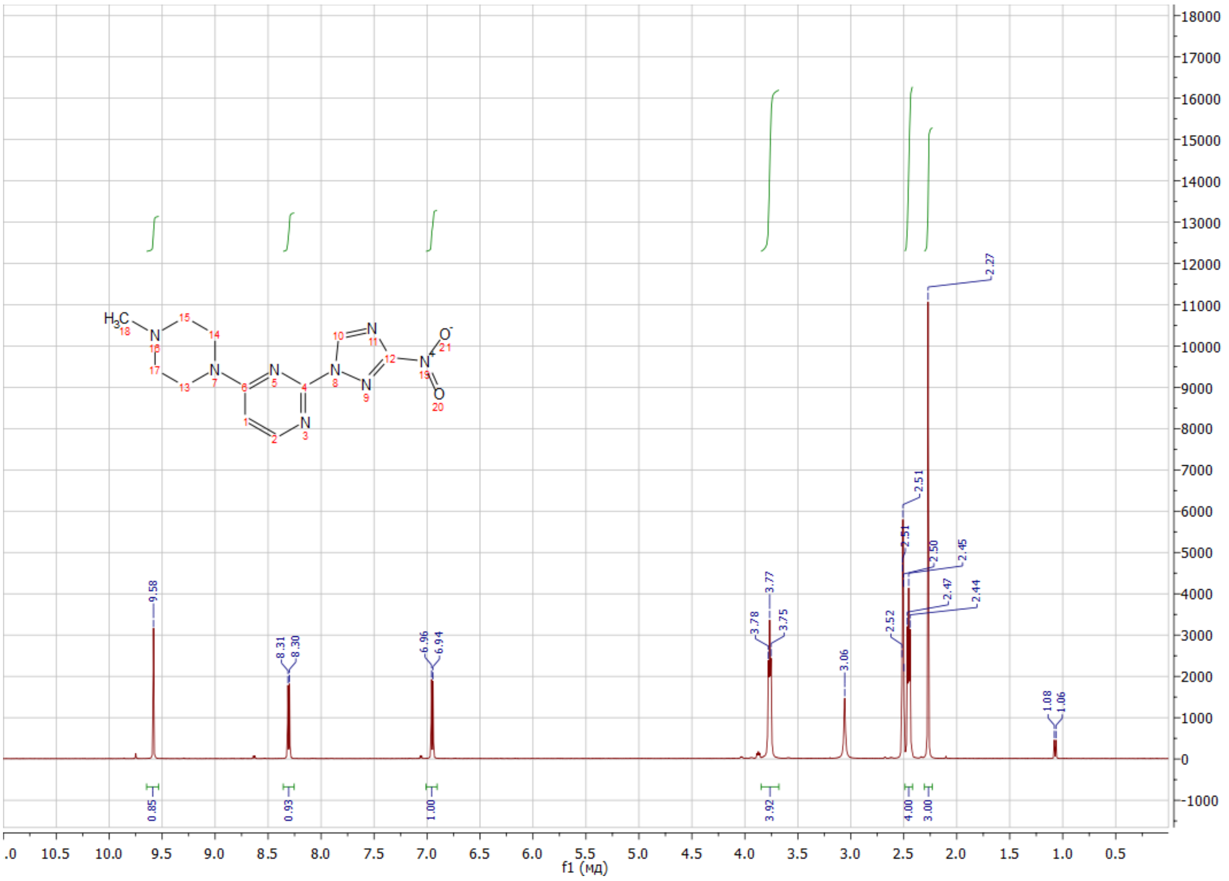

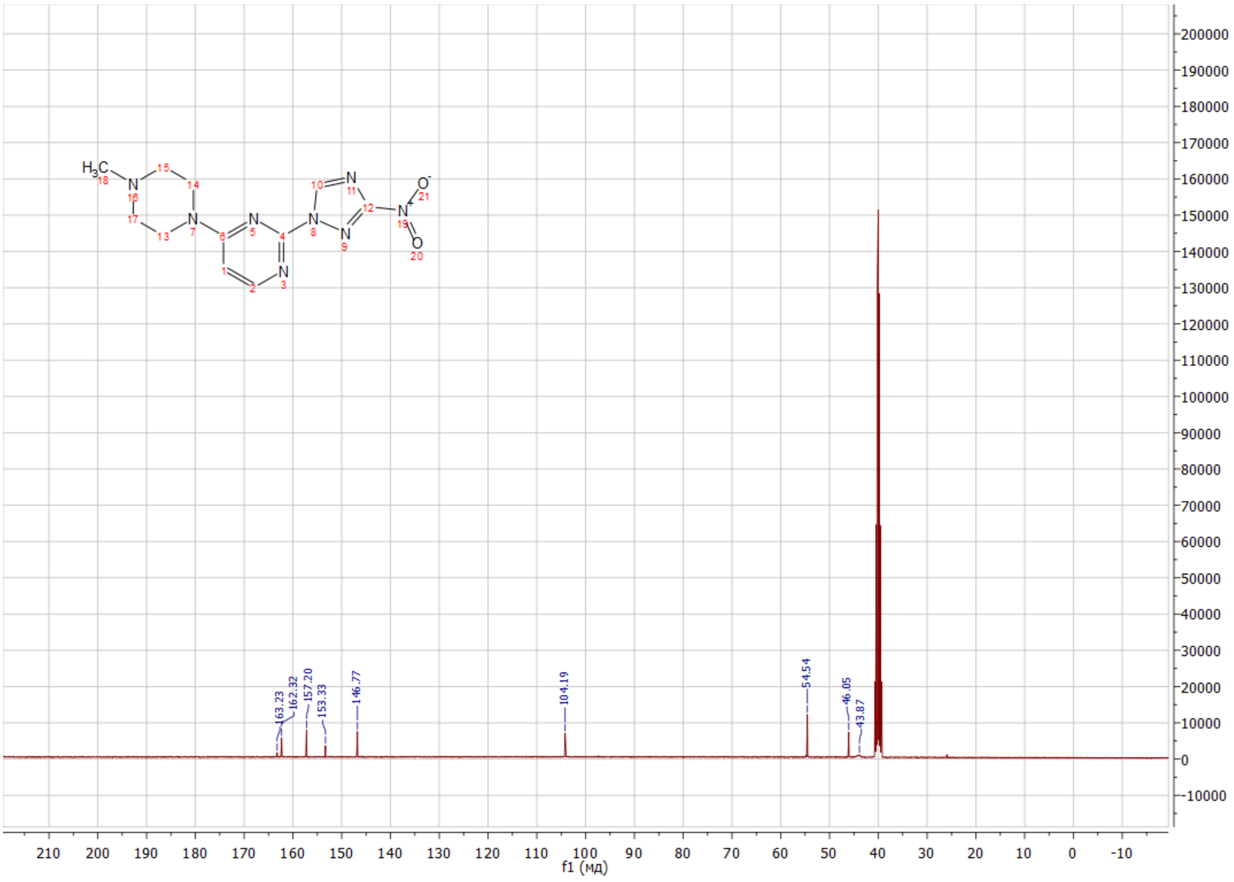

^{1}H and ^{13}C NMR of *N*-benzyl-2-(3-nitro-1*H*-1,2,4-triazol-1-yl)pyrimidin-4-amine (1j)

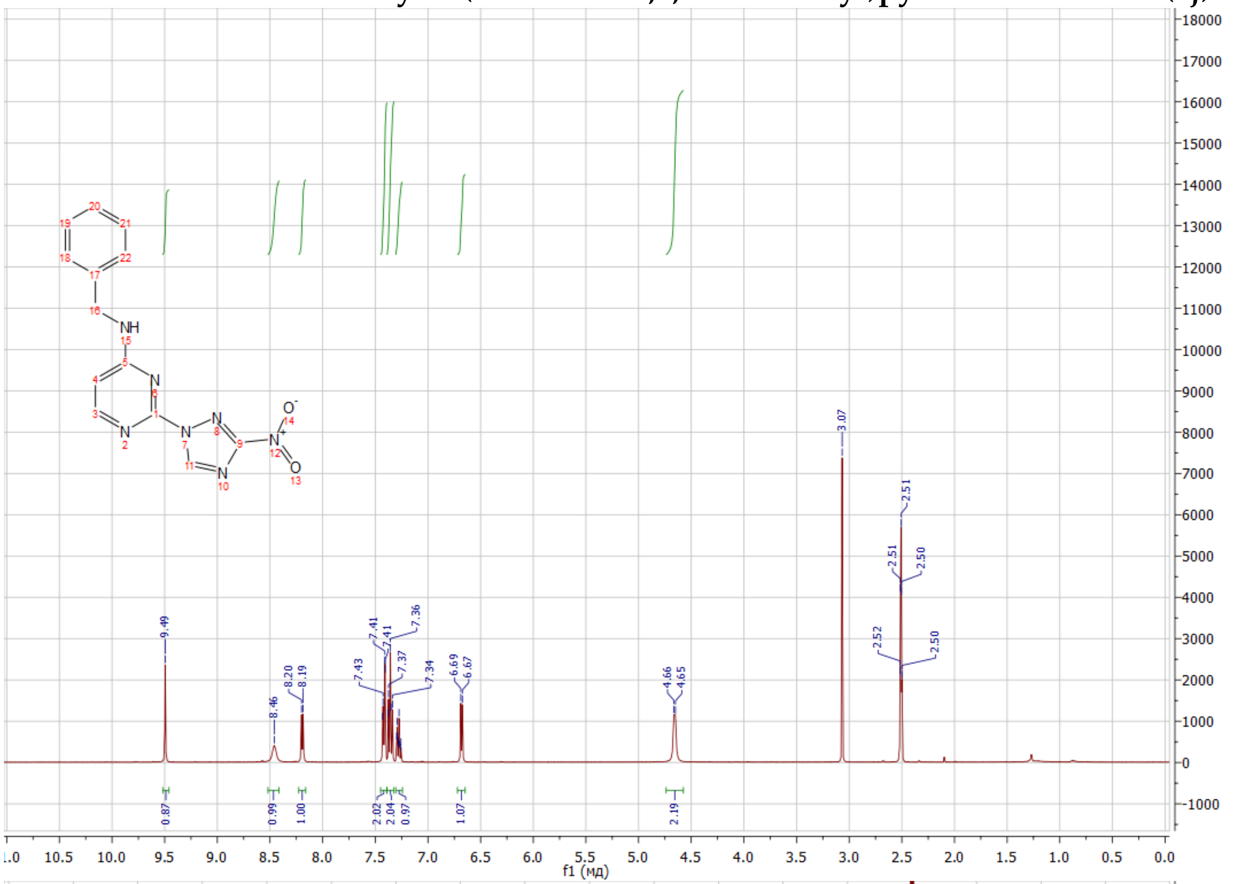

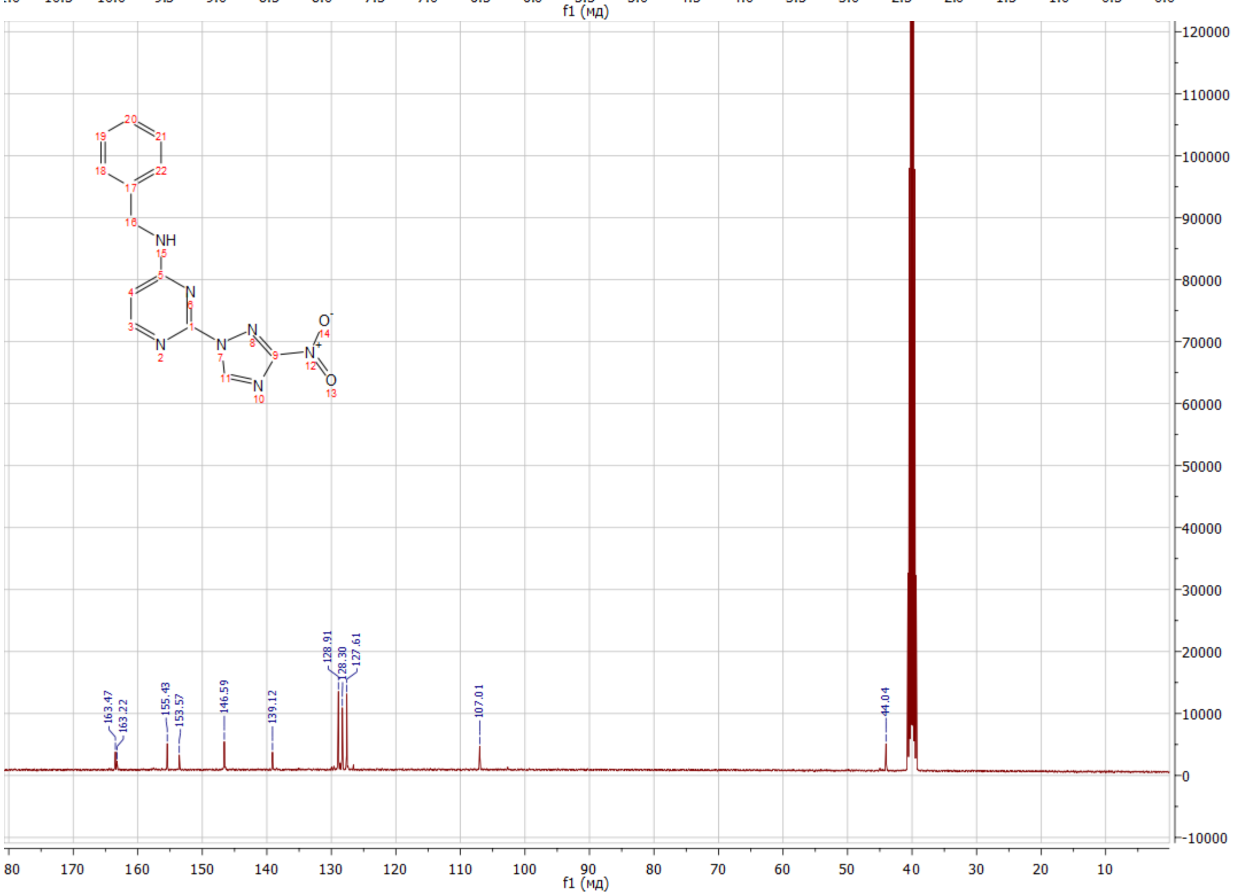

^{1}H and ^{13}C NMR of *N*-isobutyl-2-(3-nitro-1*H*-1,2,4-triazol-1-yl)pyrimidin-4-amine (1k)

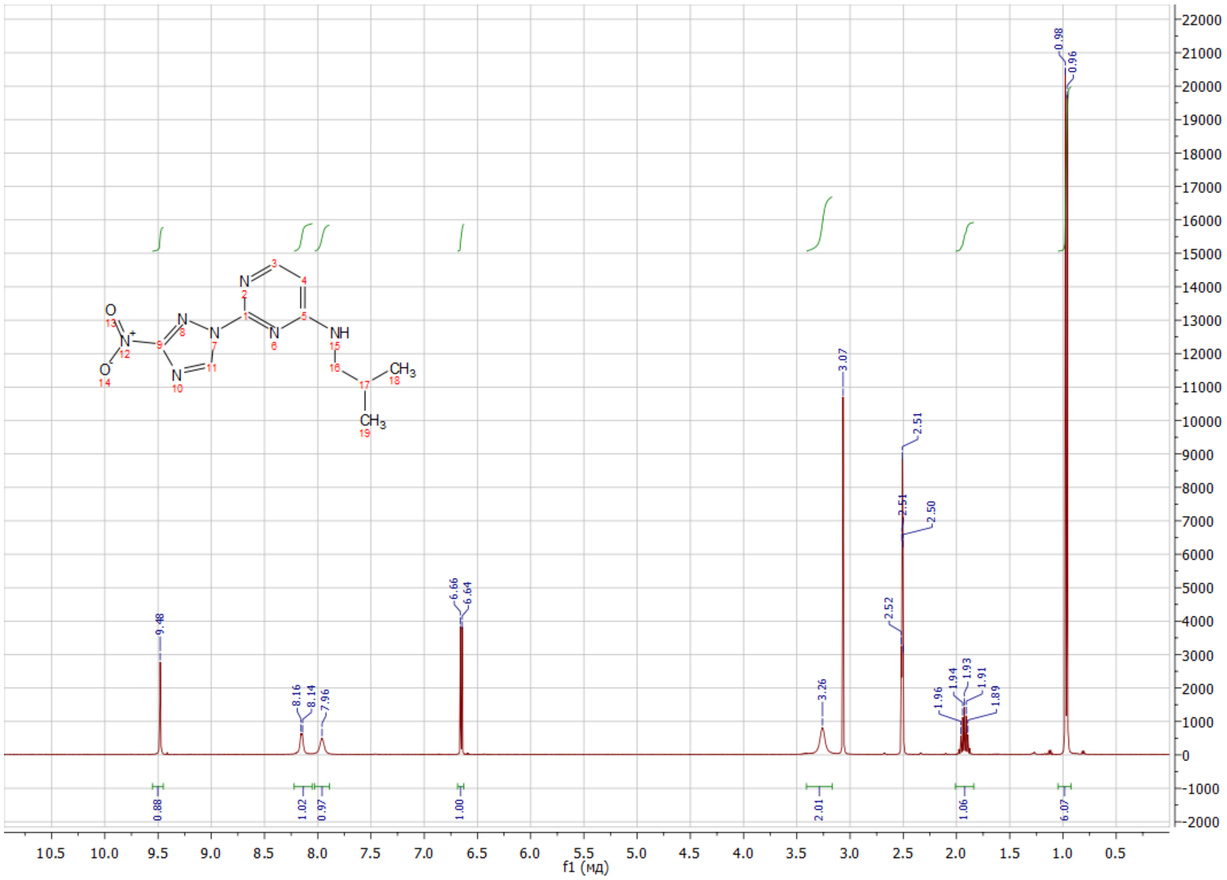

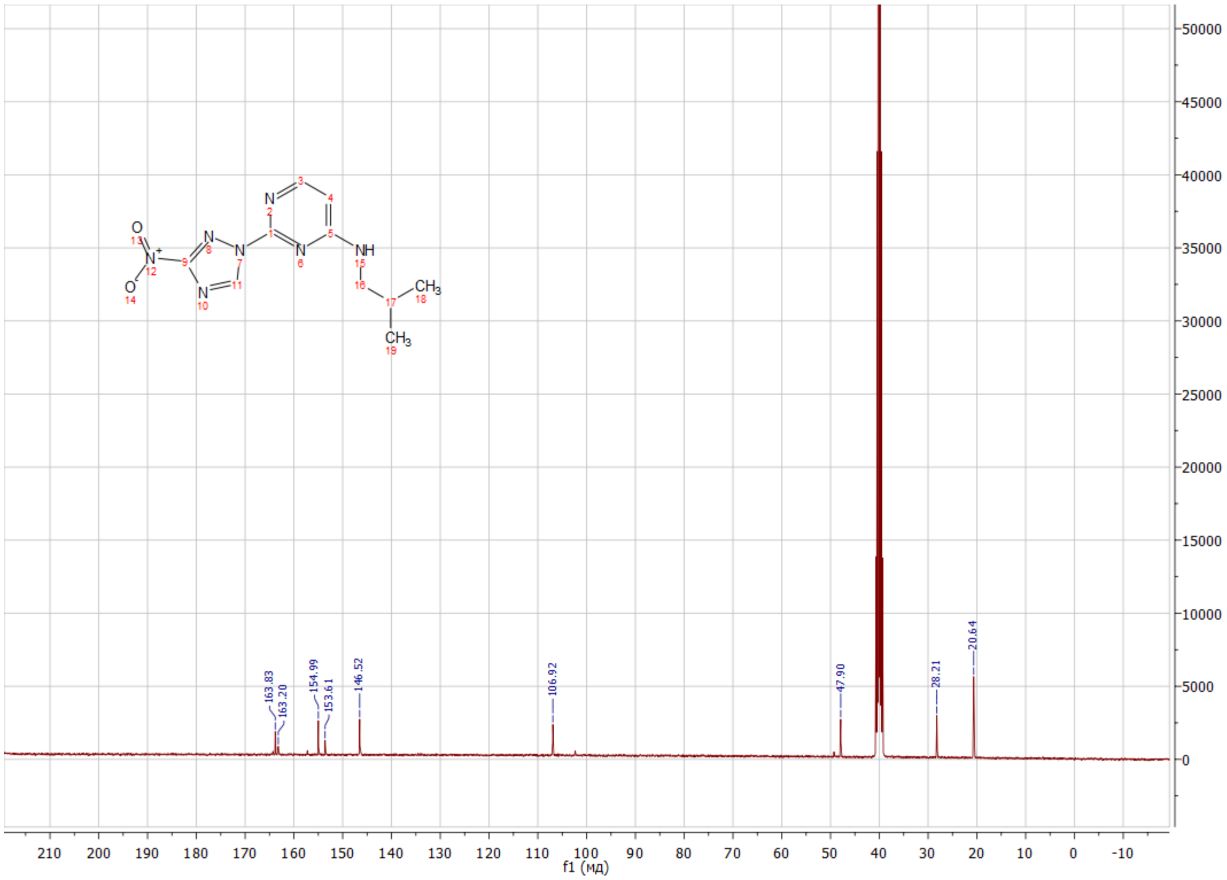

^{1}H and ^{13}C NMR of 2,2'-((2-(3-nitro-1*H*-1,2,4-triazol-1-yl)pyrimidin-4-yl)azanediyl)bis(ethan-1-ol) (1l)

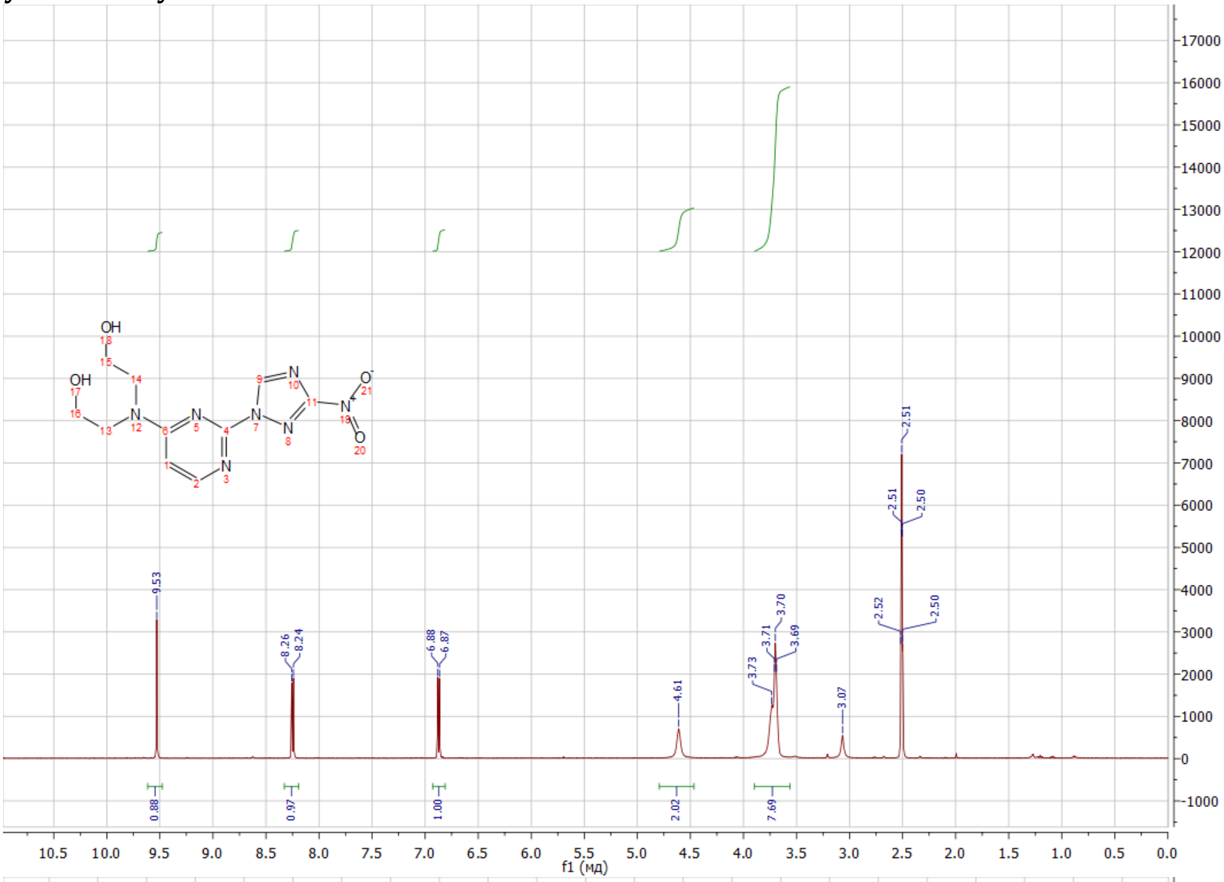

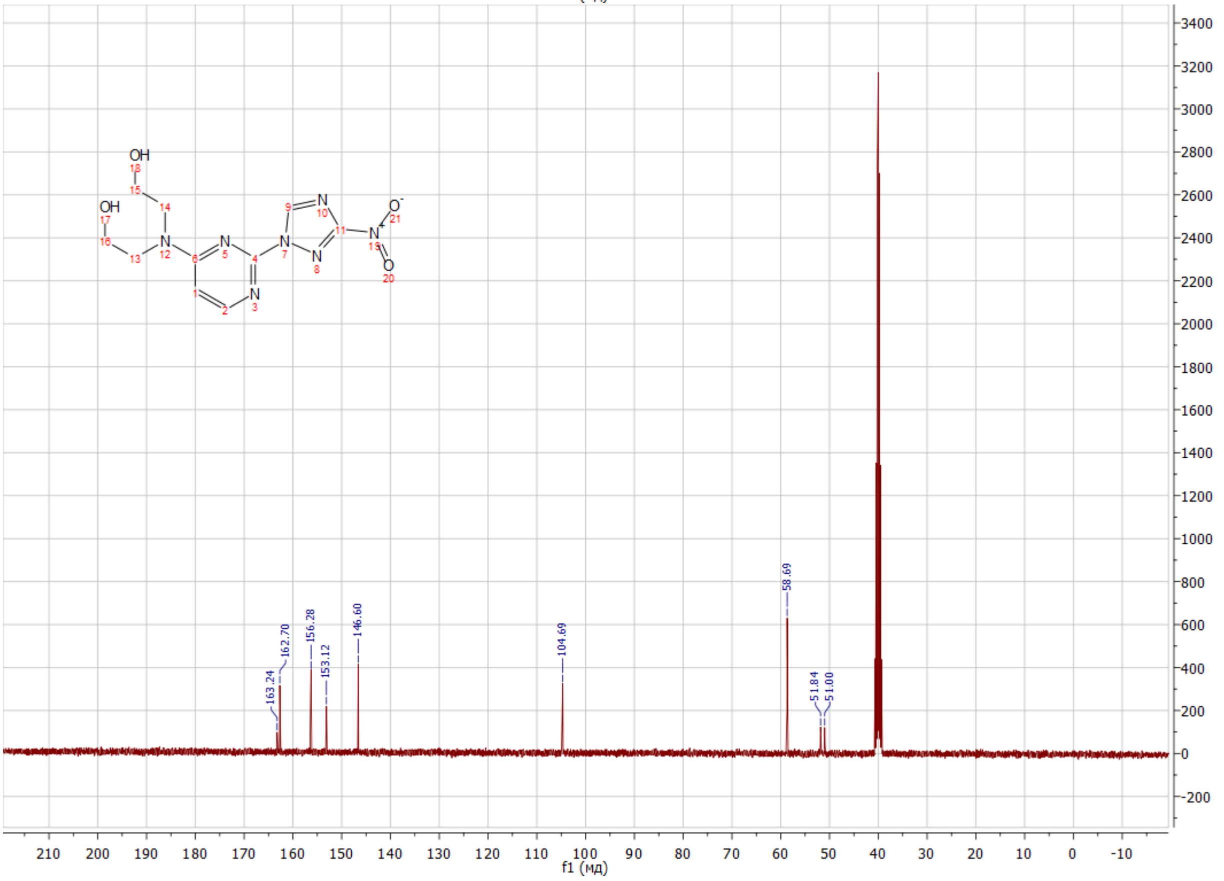

^{1}H and ^{13}C NMR of 2-(3-nitro-1*H*-1,2,4-triazol-1-yl)-*N*-(p-tolyl)pyrimidin-4-amine (1m)

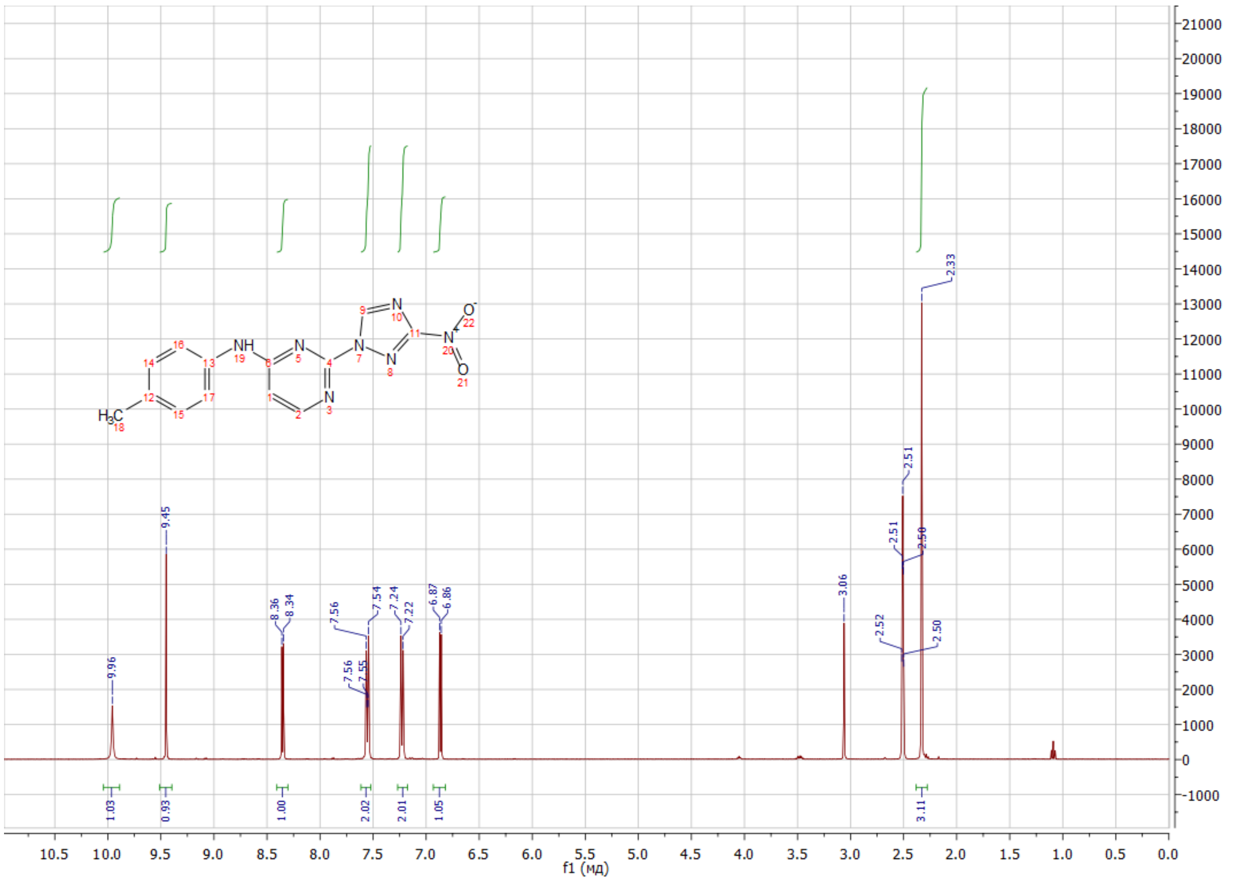

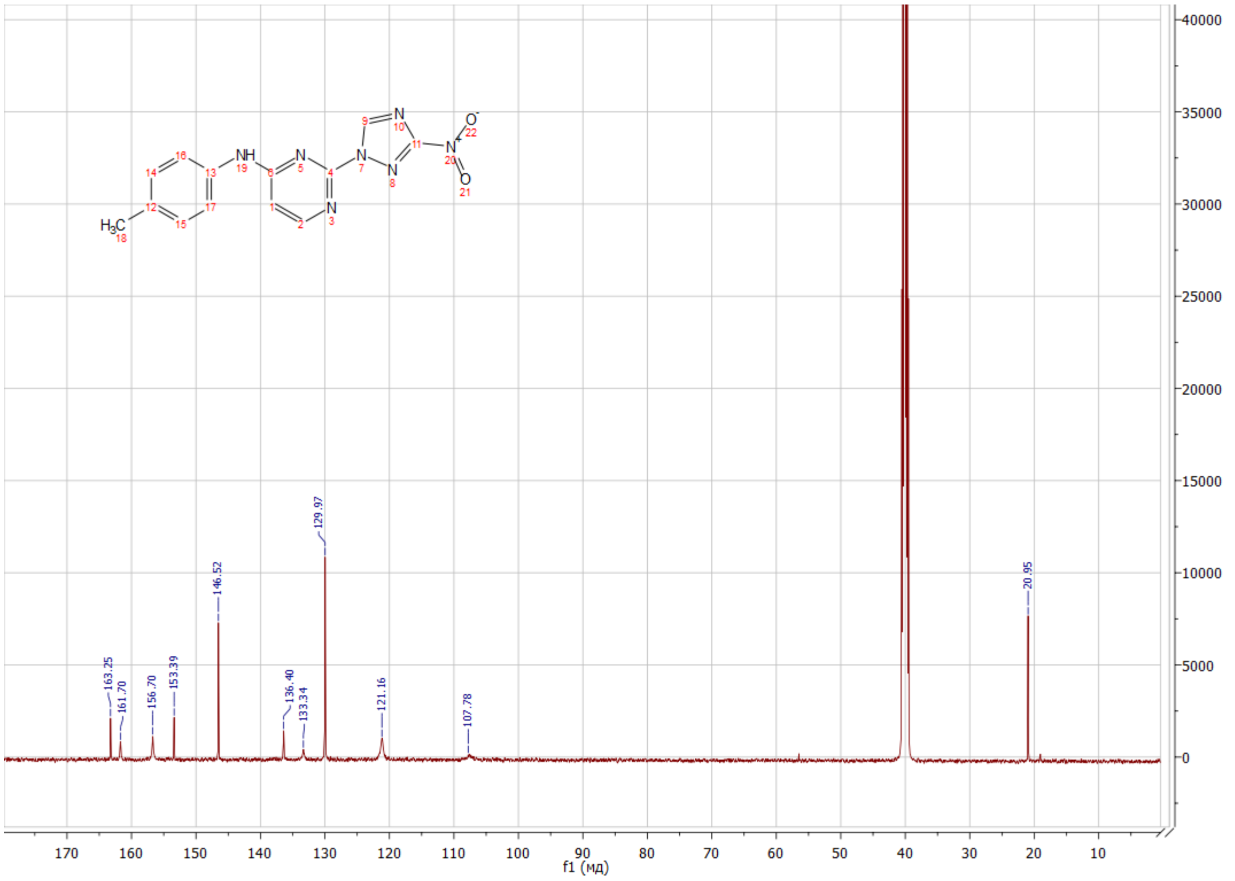

^{1}H and ^{13}C NMR of *N*-cyclopropyl-2-(3-nitro-1*H*-1,2,4-triazol-1-yl)pyrimidin-4-amine (1n)

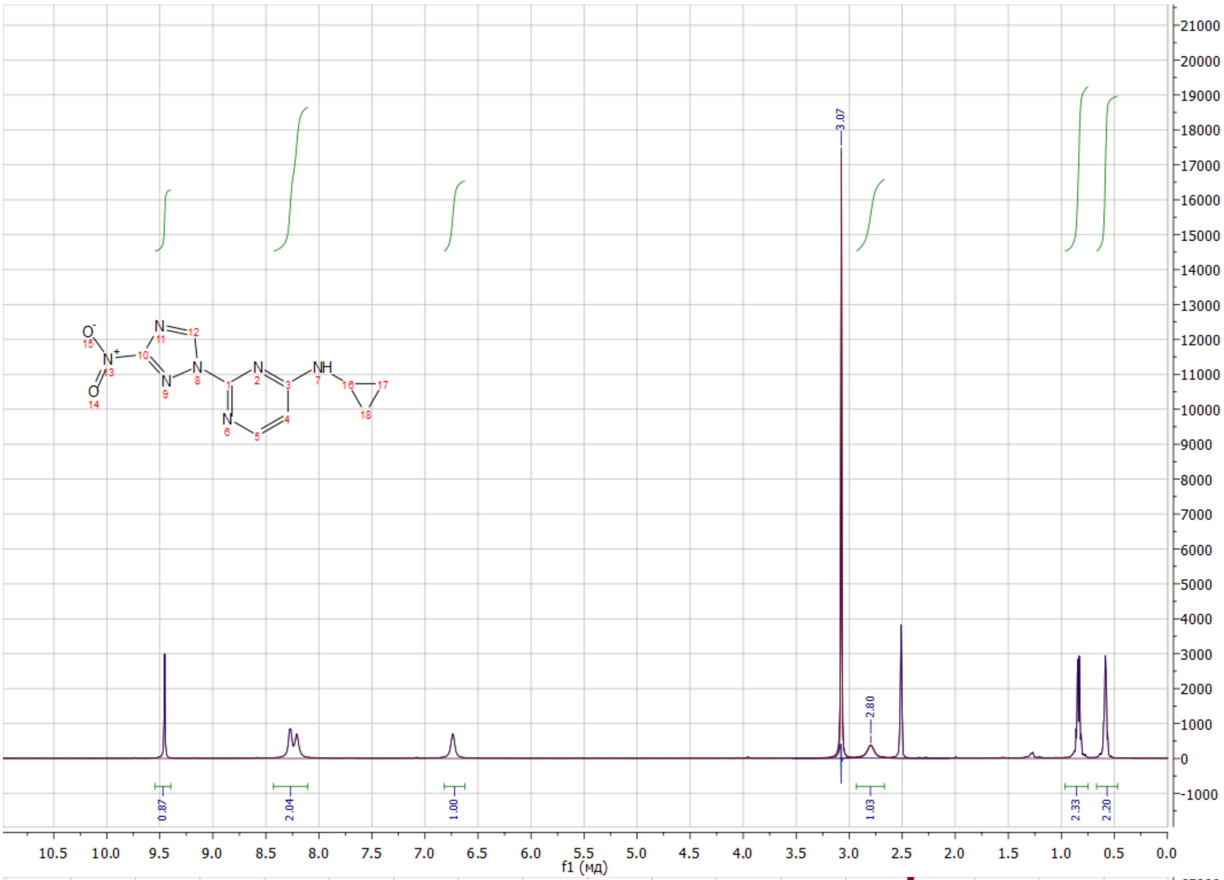

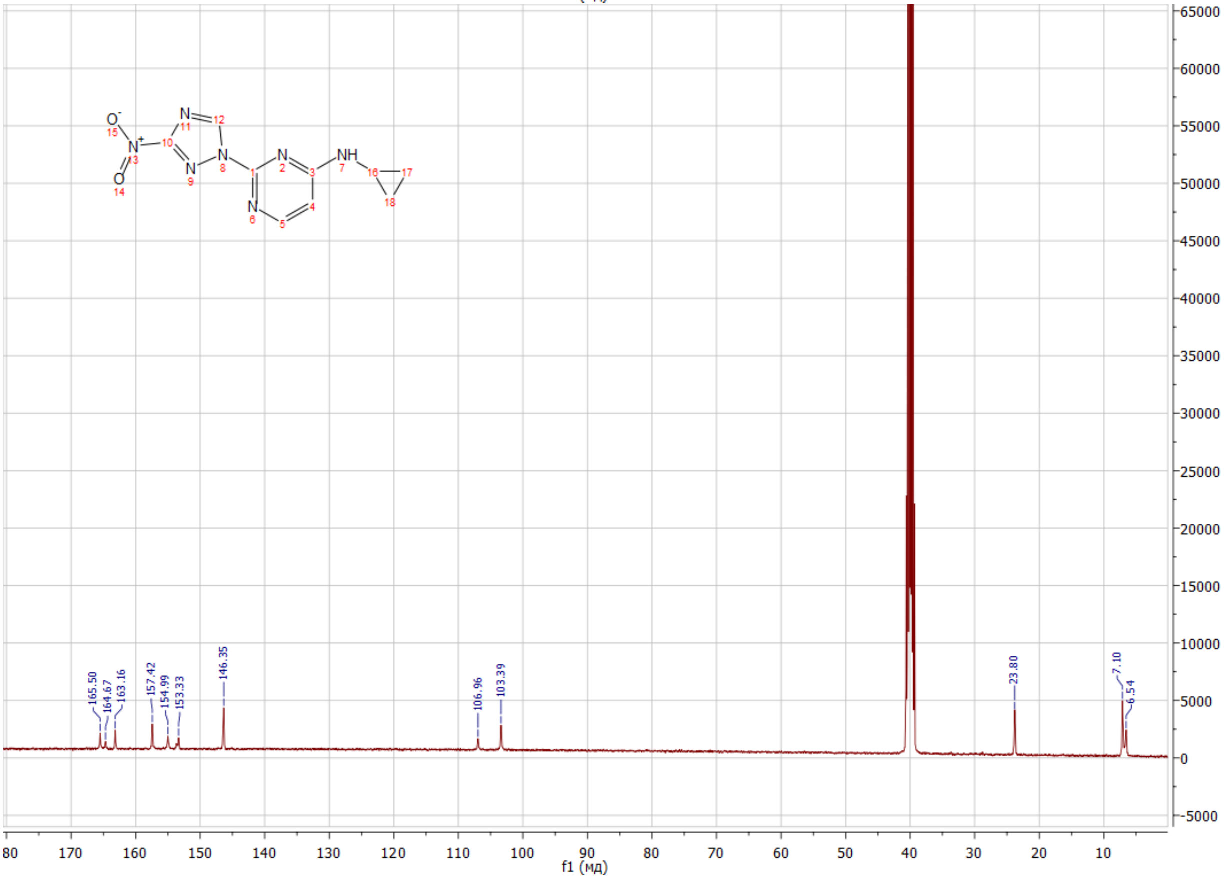

^{1}H and ^{13}C NMR of *N*-isopropyl-2-(3-nitro-1*H*-1,2,4-triazol-1-yl)pyrimidin-4-amine (1o)

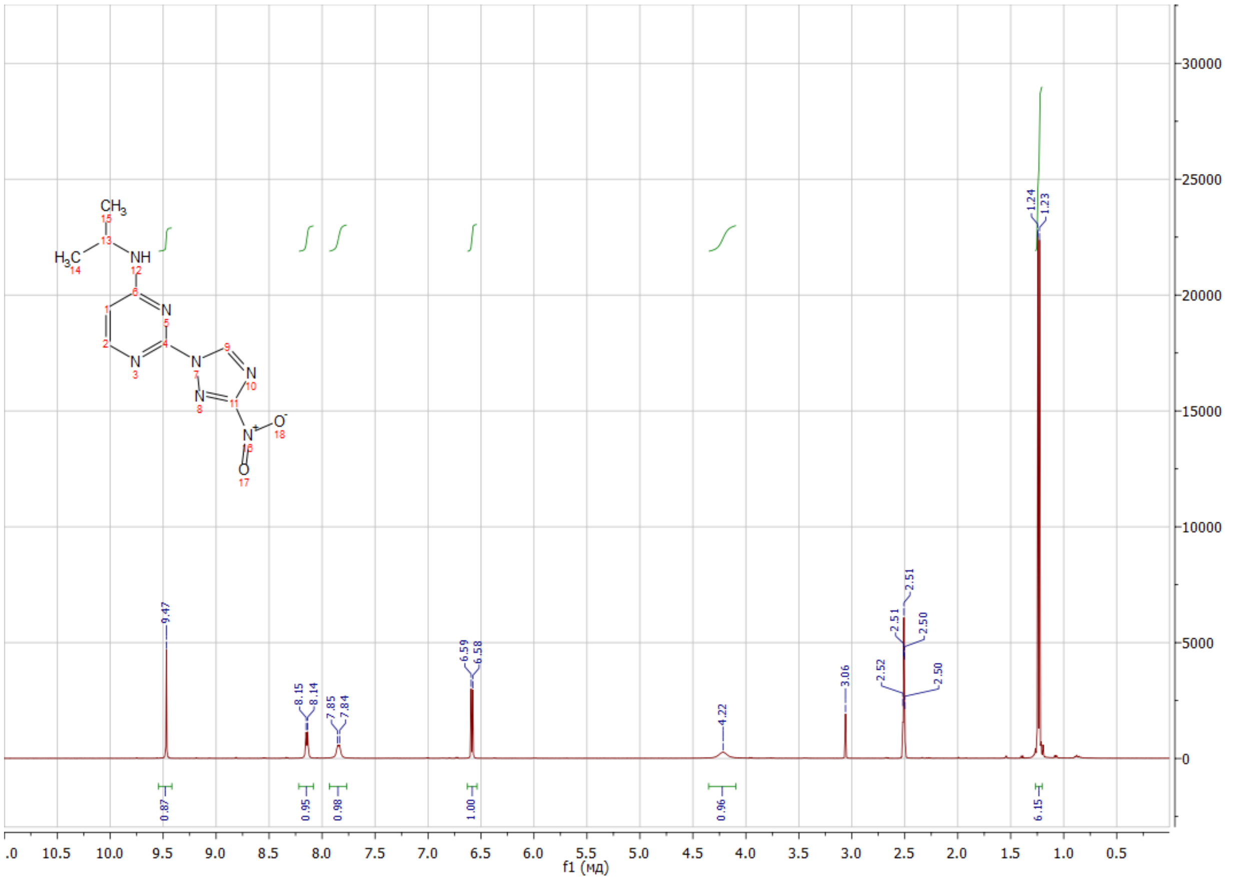

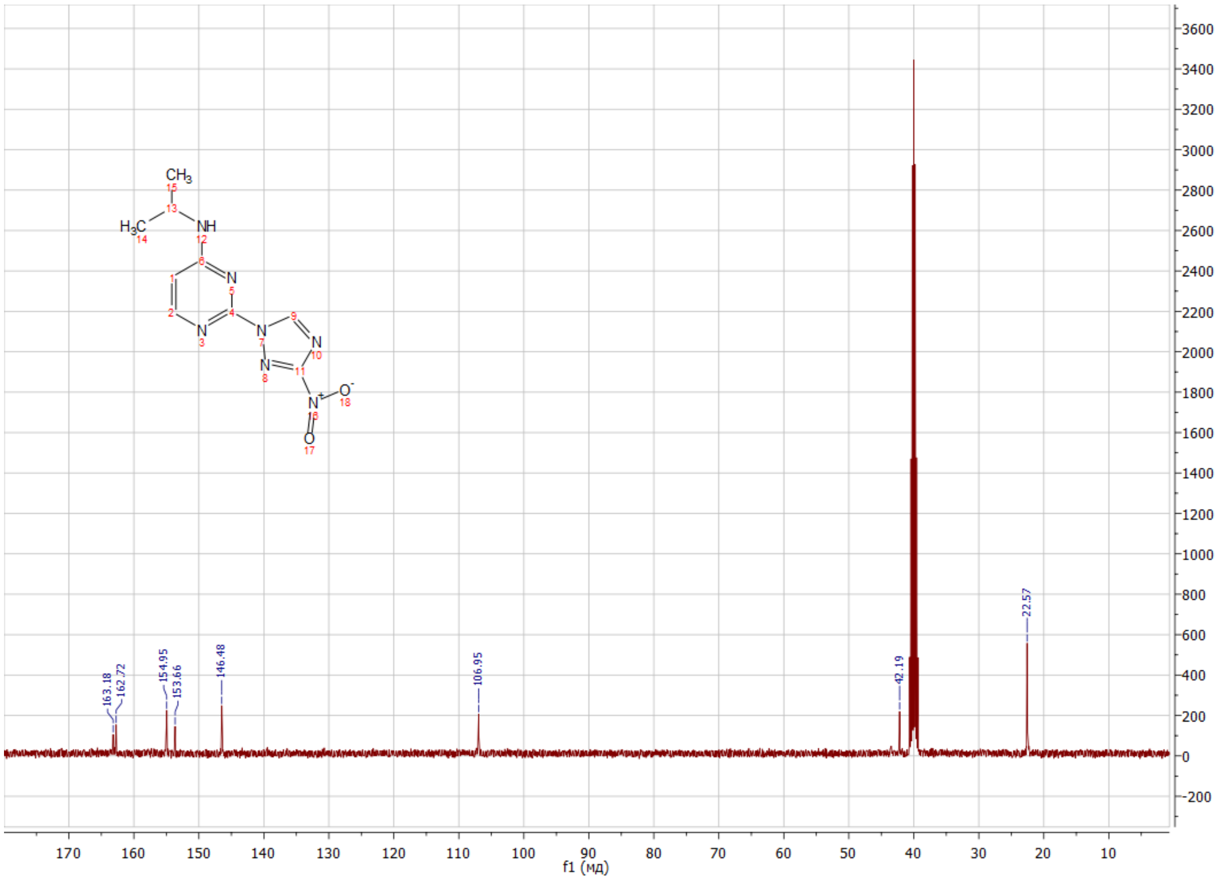

^{1}H and ^{13}C NMR of *N*-(3-(1*H*-imidazol-1-yl)propyl)-2-(3-nitro-1*H*-1,2,4-triazol-1-yl)pyrimidin-4-amine (1p)

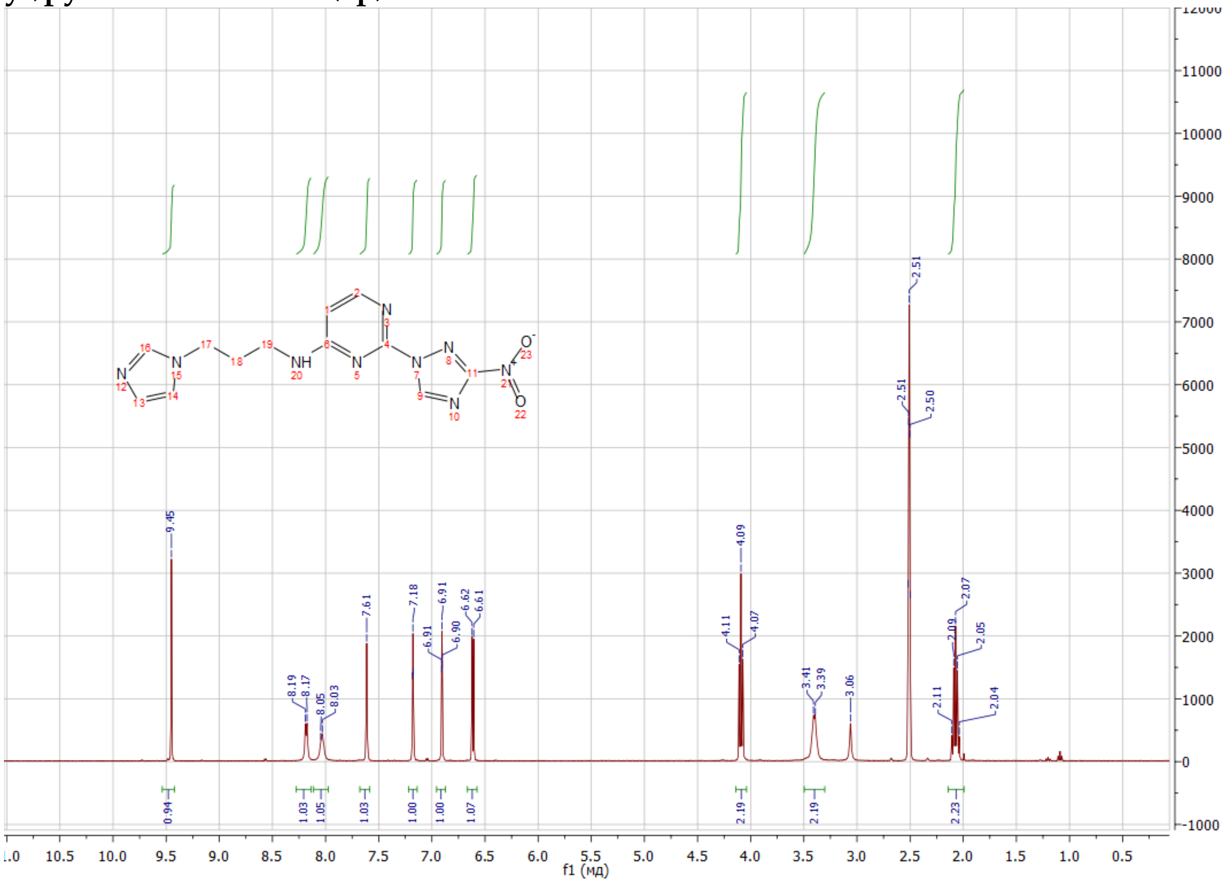

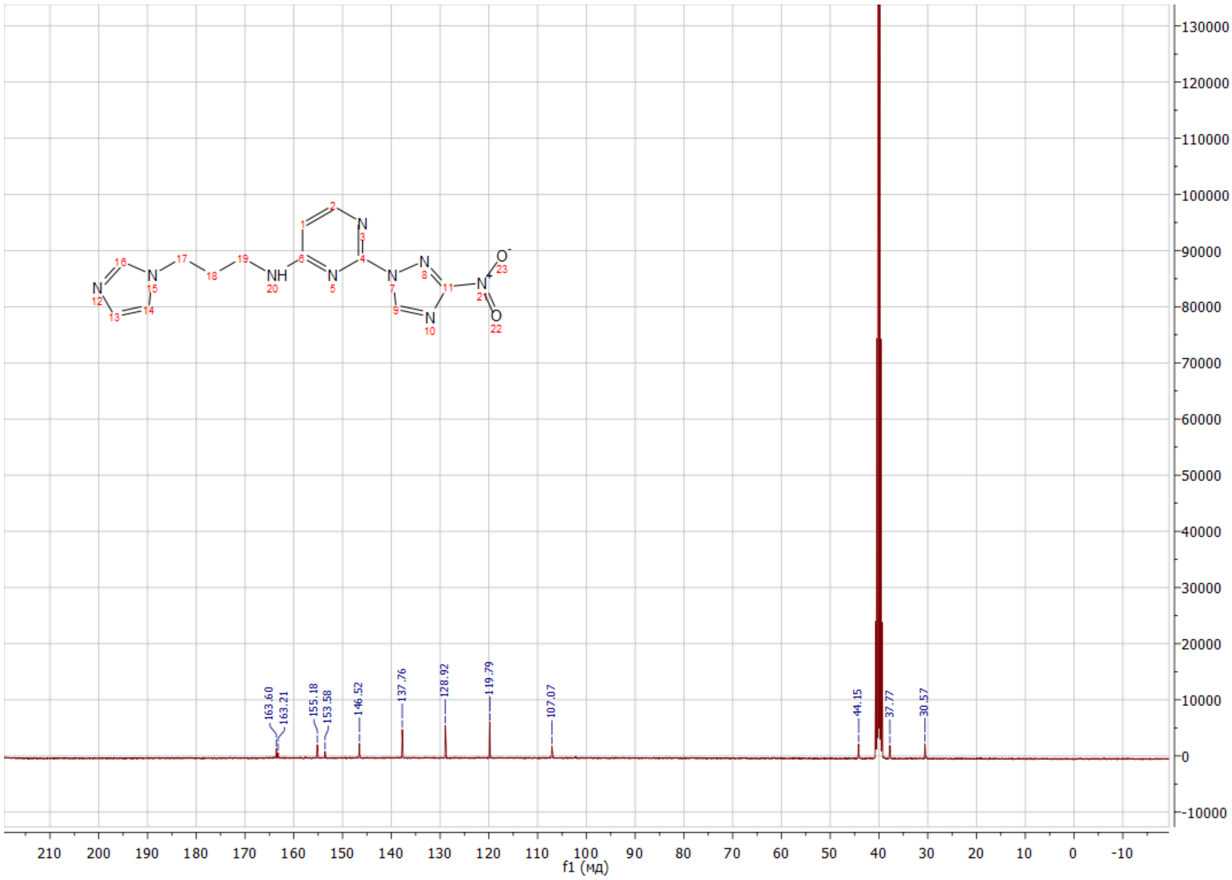

¹H and ¹³C NMR of 2-(3-nitro-1*H*-1,2,4-triazol-1-yl)-4-(pyrrolidin-1-yl)pyrimidine (1q)

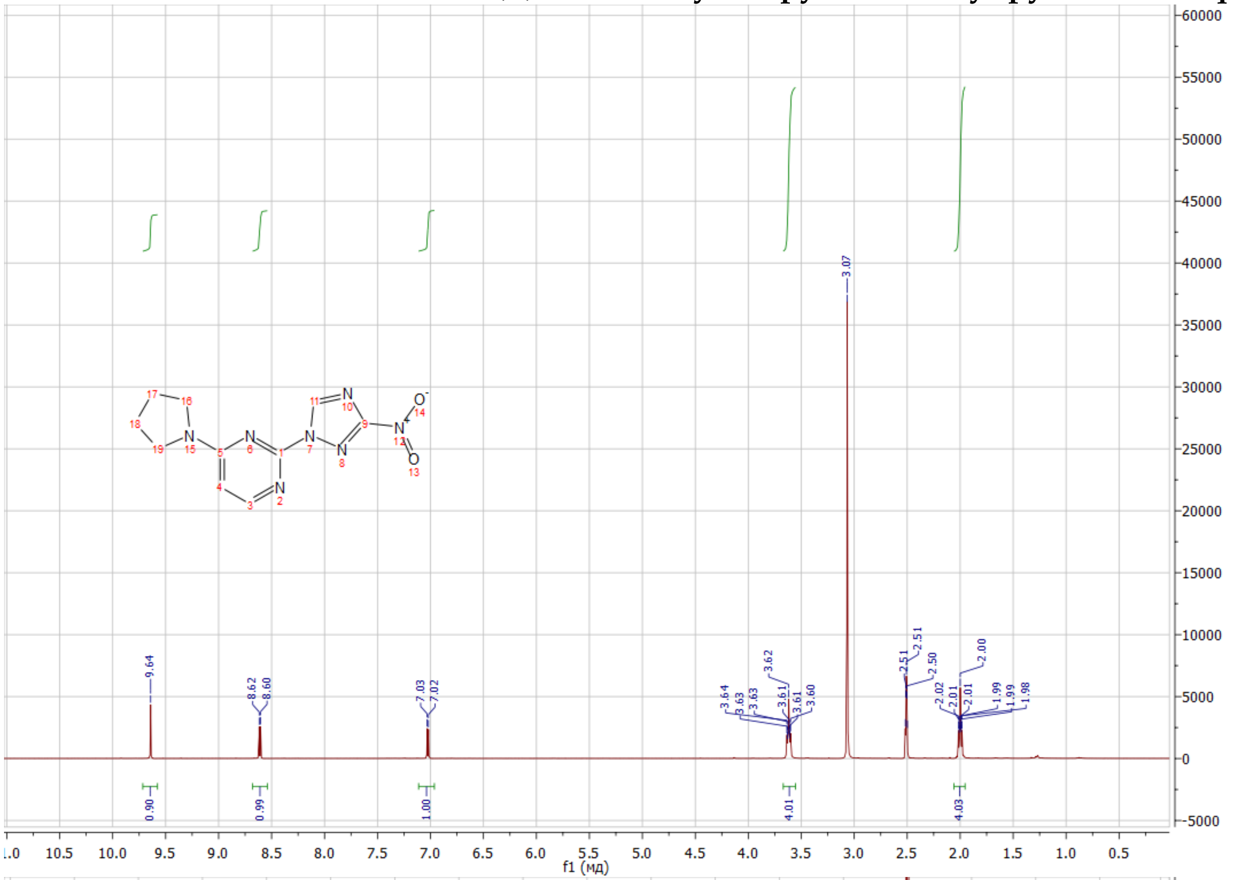

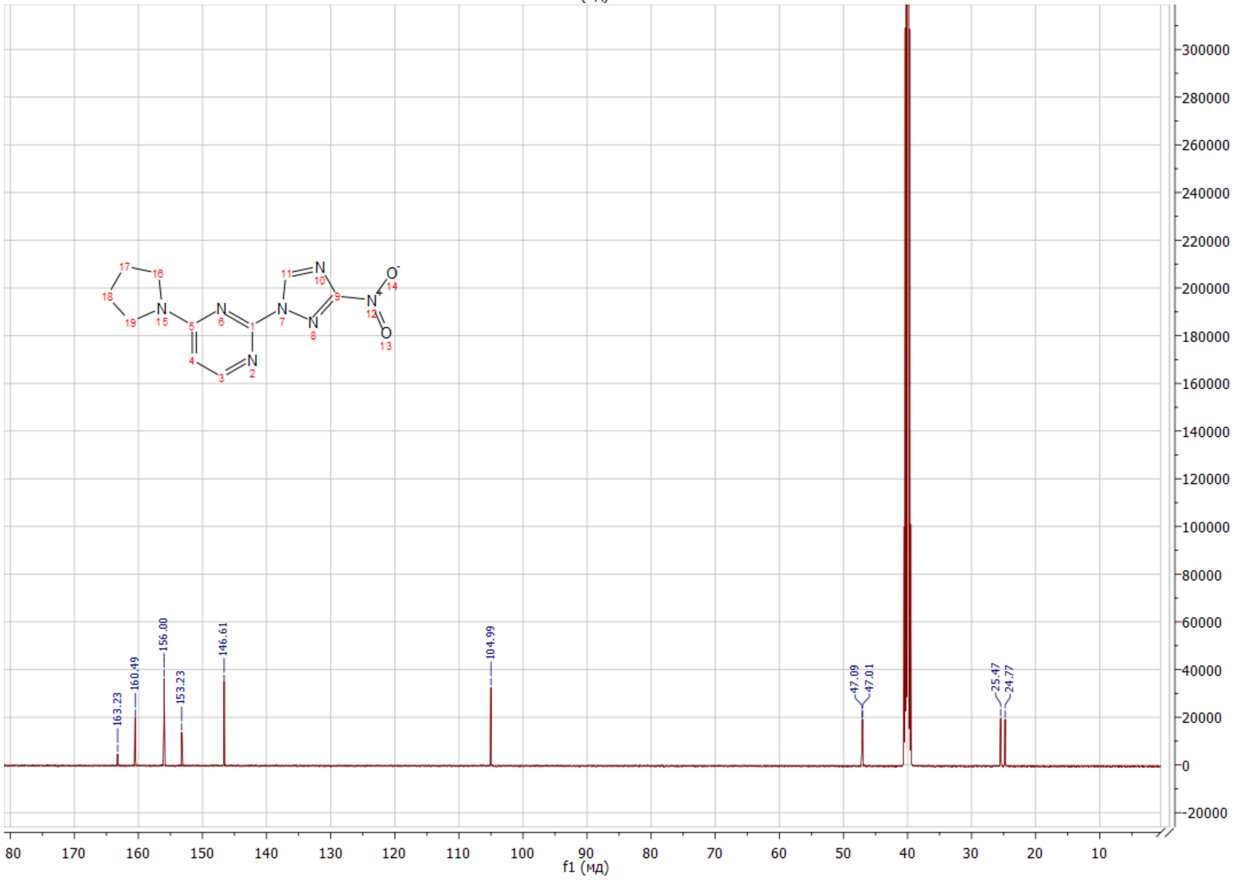

^{1}H and ^{13}C NMR of 2-(3-nitro-1*H*-1,2,4-triazol-1-yl)-*N*-propylpyrimidin-4-amine (1r)

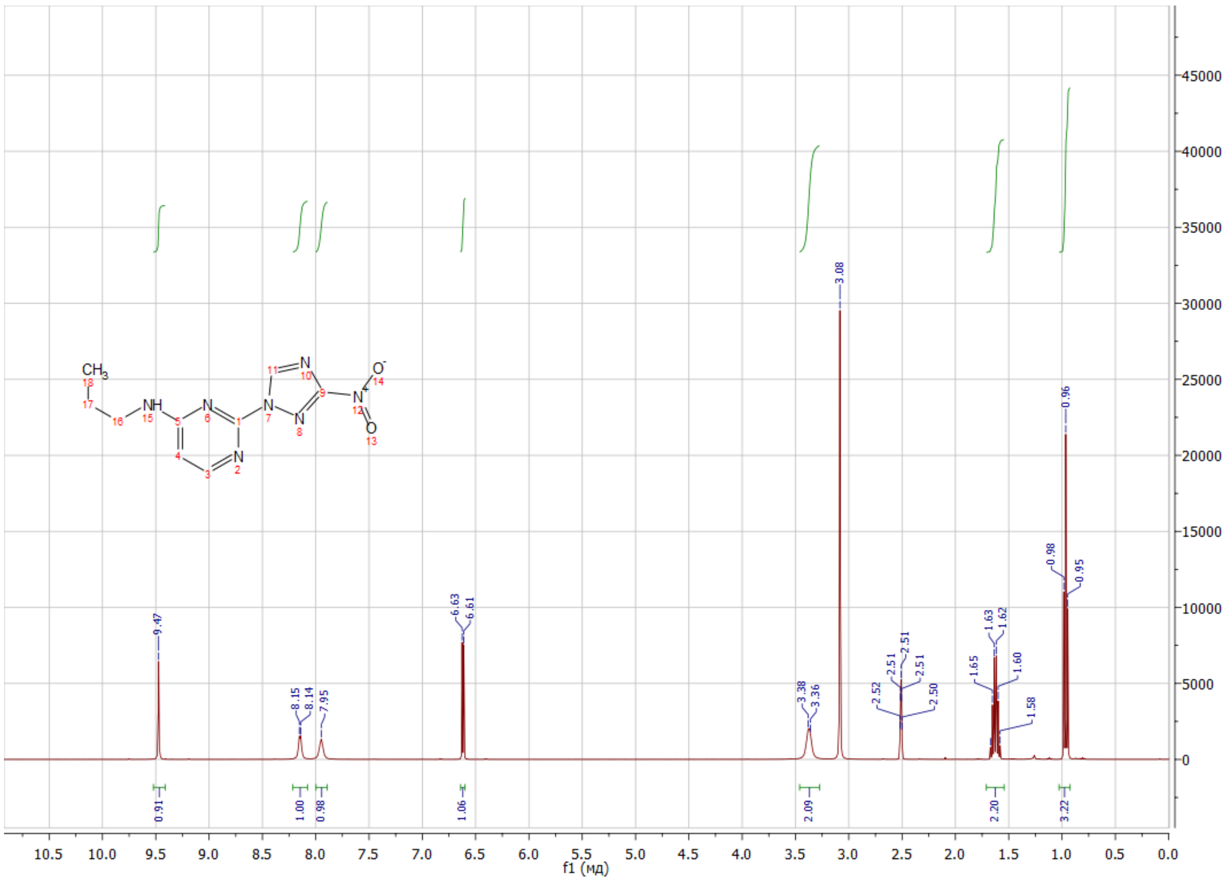

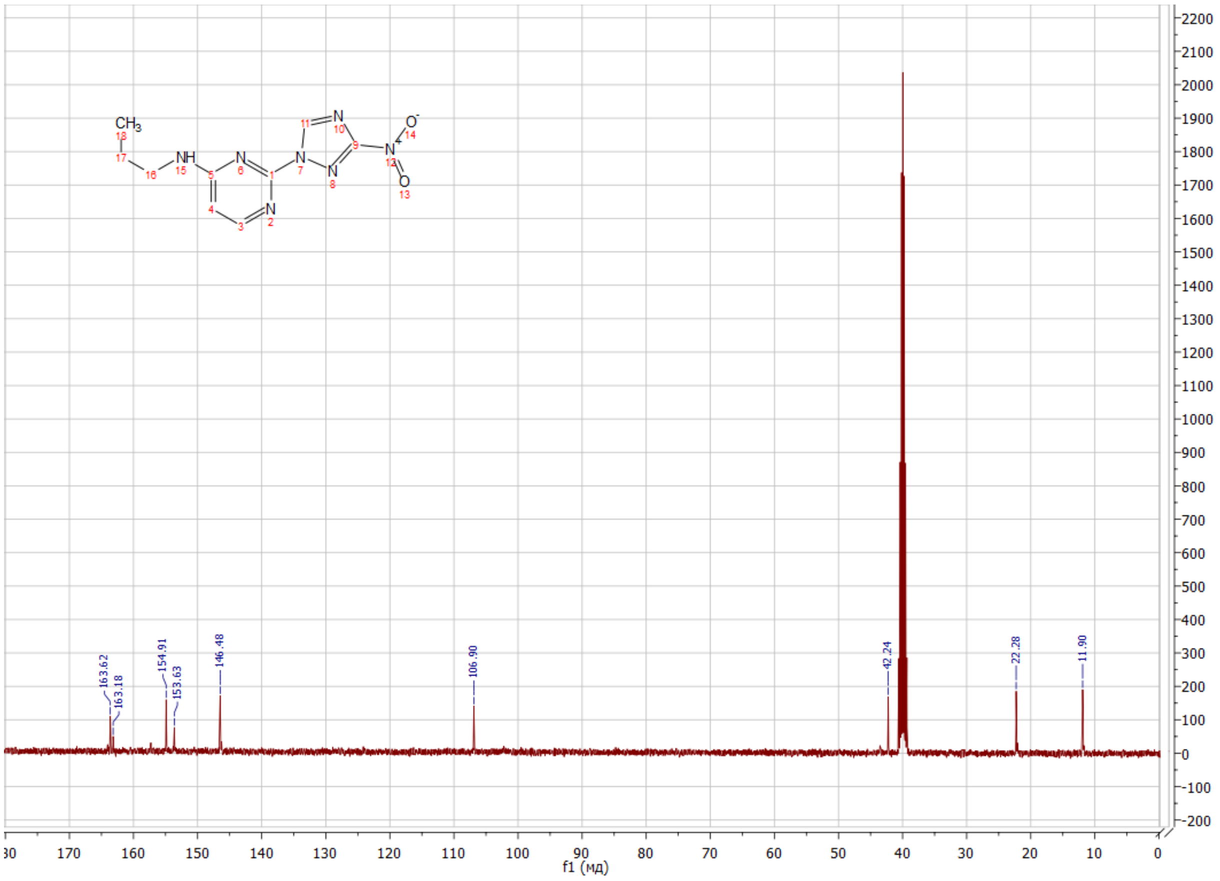

^{1}H and ^{13}C NMR of *N*-(4-methoxybenzyl)-2-(3-nitro-1*H*-1,2,4-triazol-1-yl)pyrimidin-4-amine (1s)

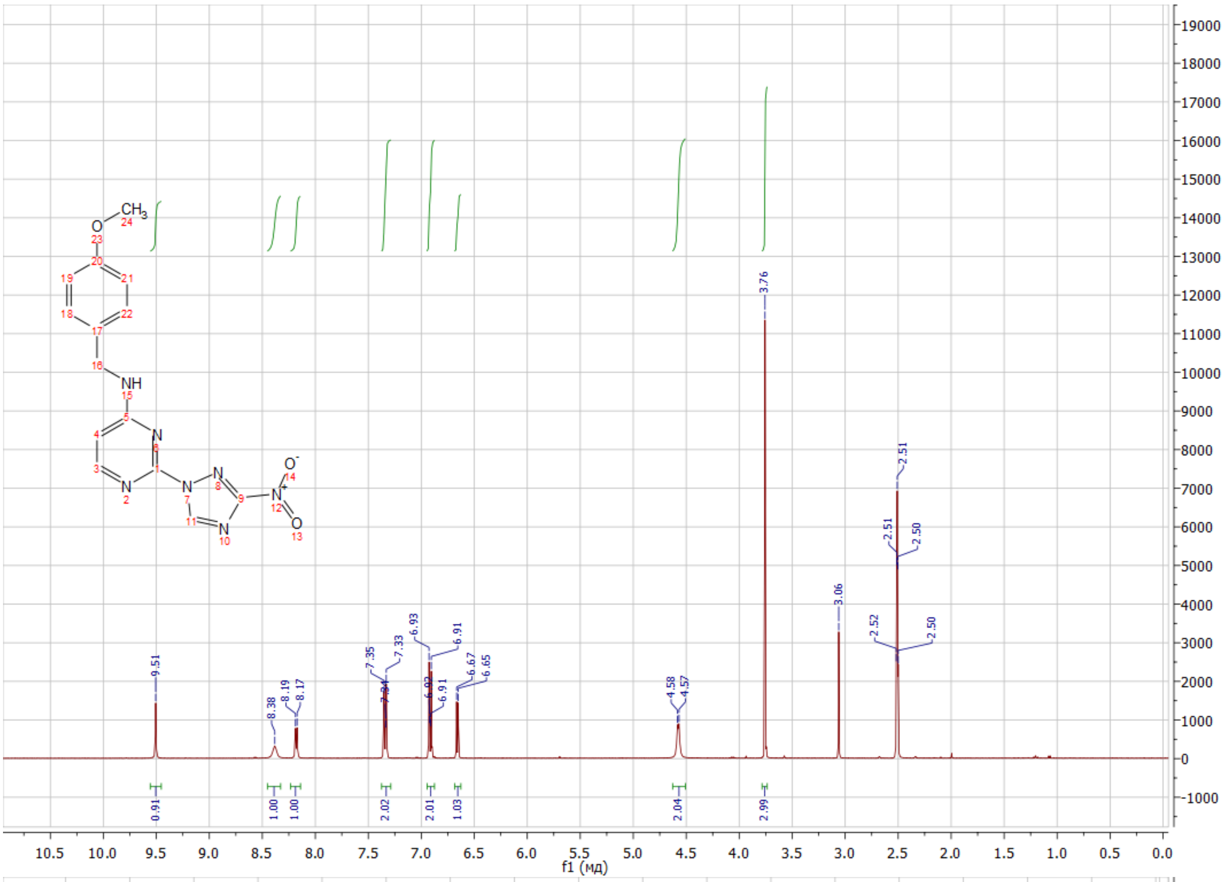

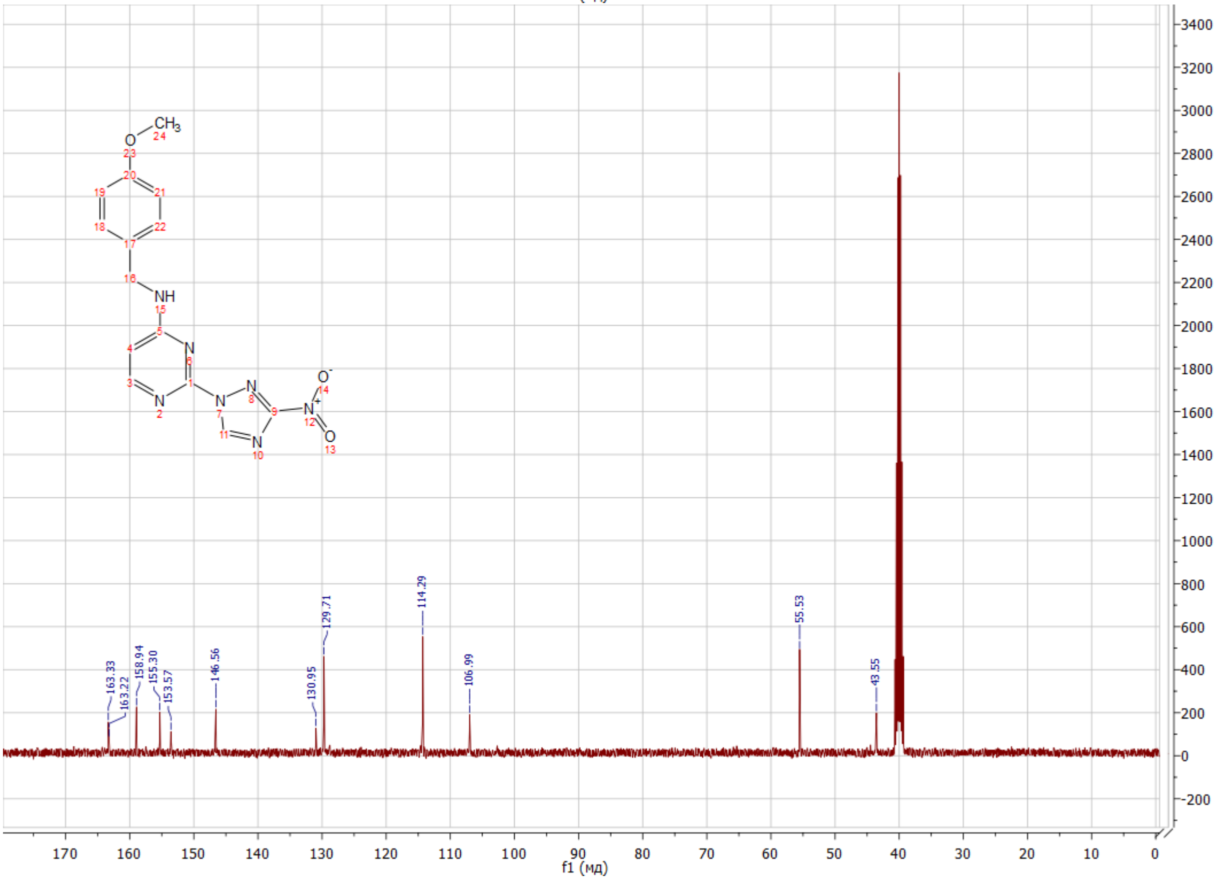

^{1}H and ^{13}C NMR of 2-chloro-4-(3-nitro-1*H*-1,2,4-triazol-1-yl)pyrimidine (5)

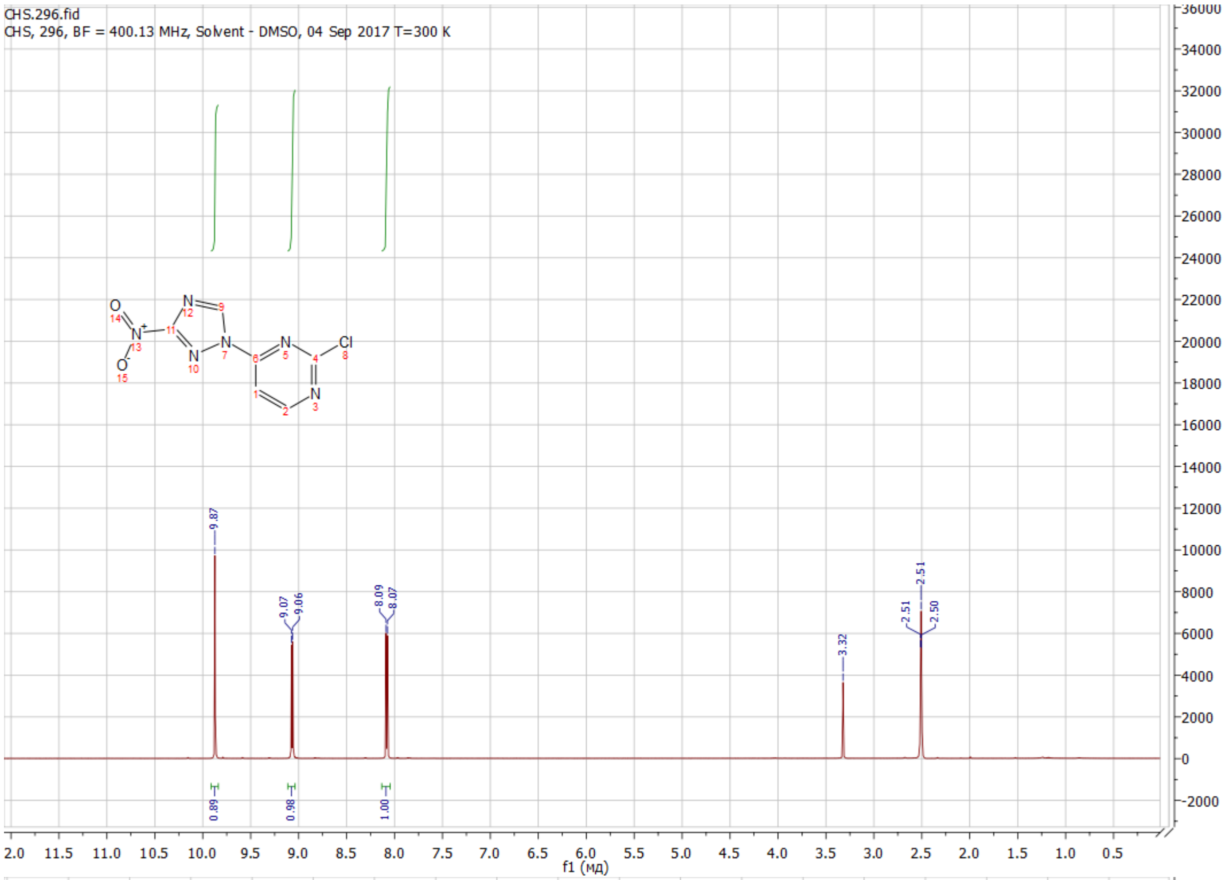

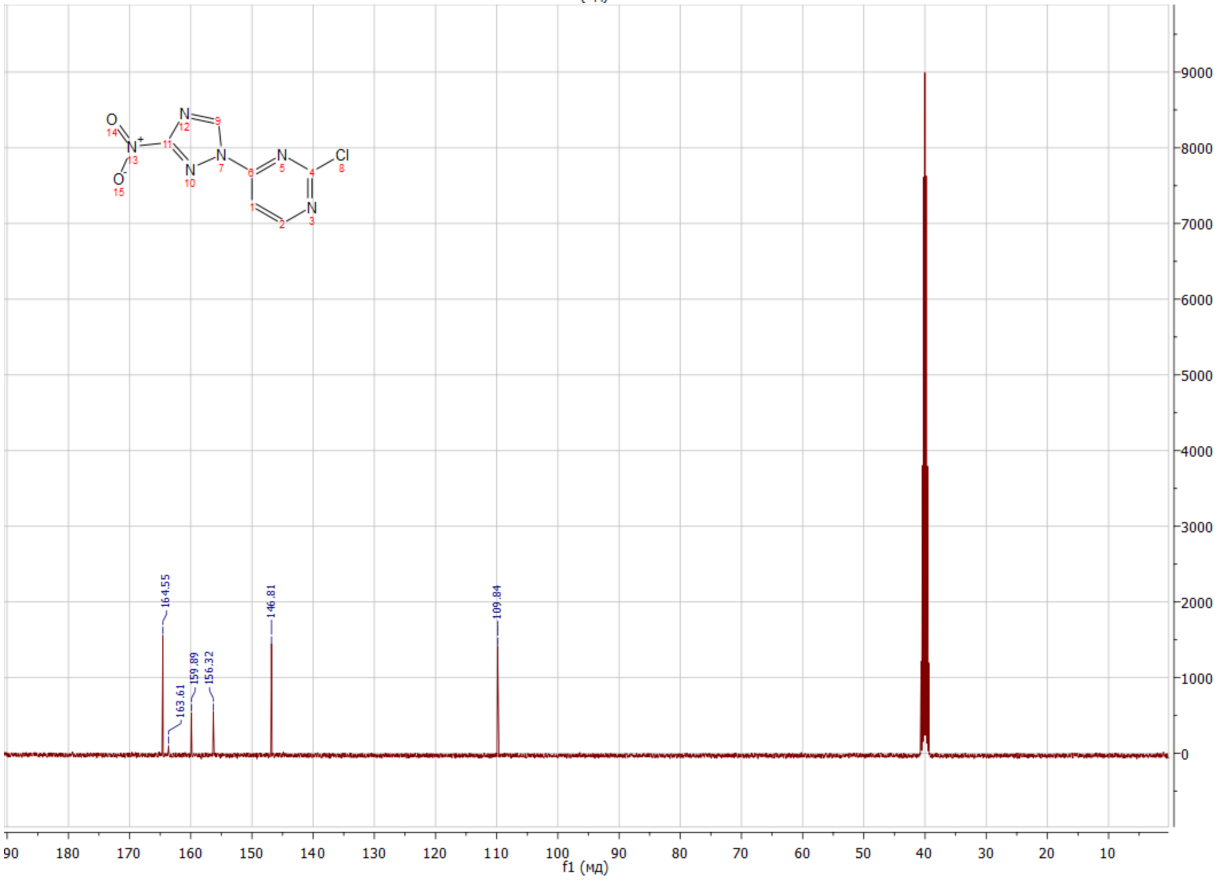

^{1}H and ^{13}C NMR of 2-((4-(3-nitro-1*H*-1,2,4-triazol-1-yl)pyrimidin-2-yl)amino)ethan-1-ol (2a)

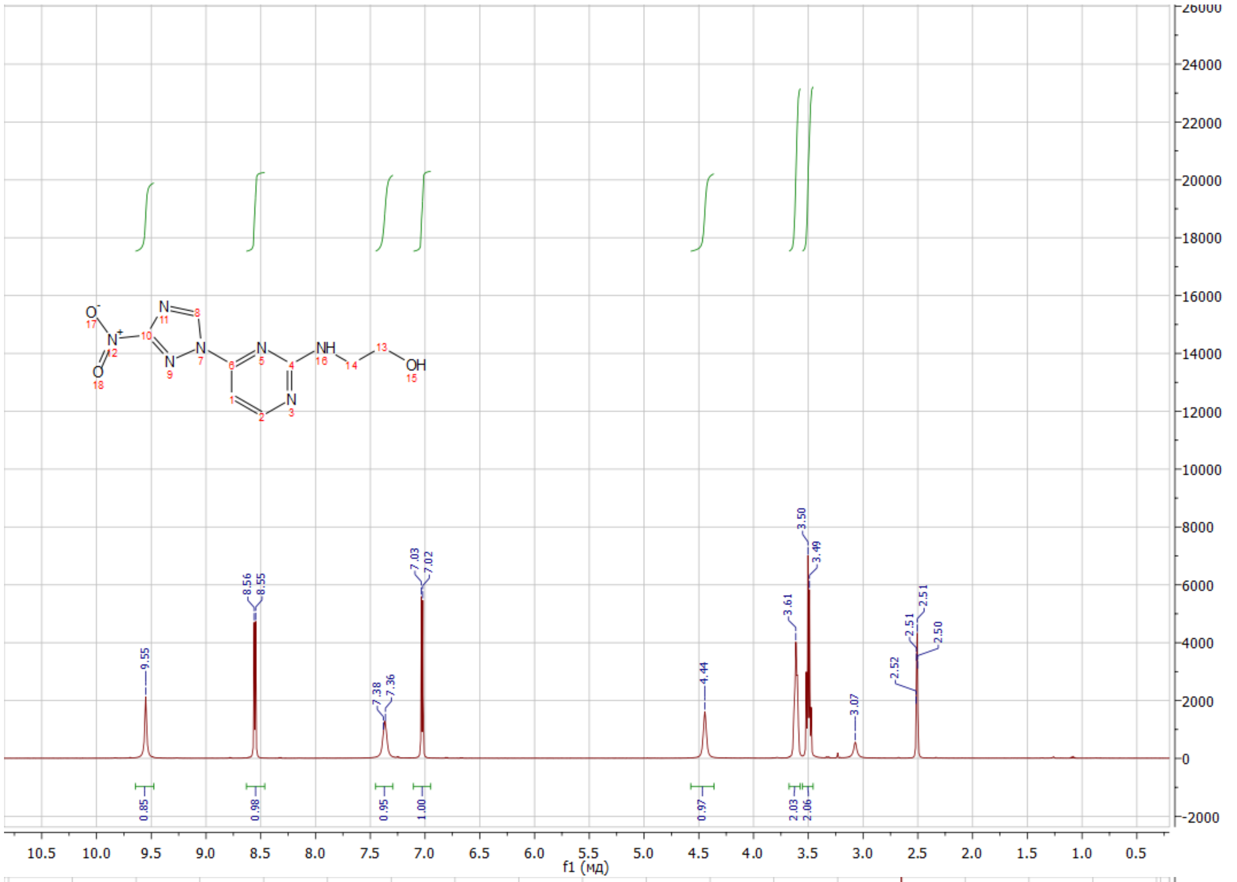

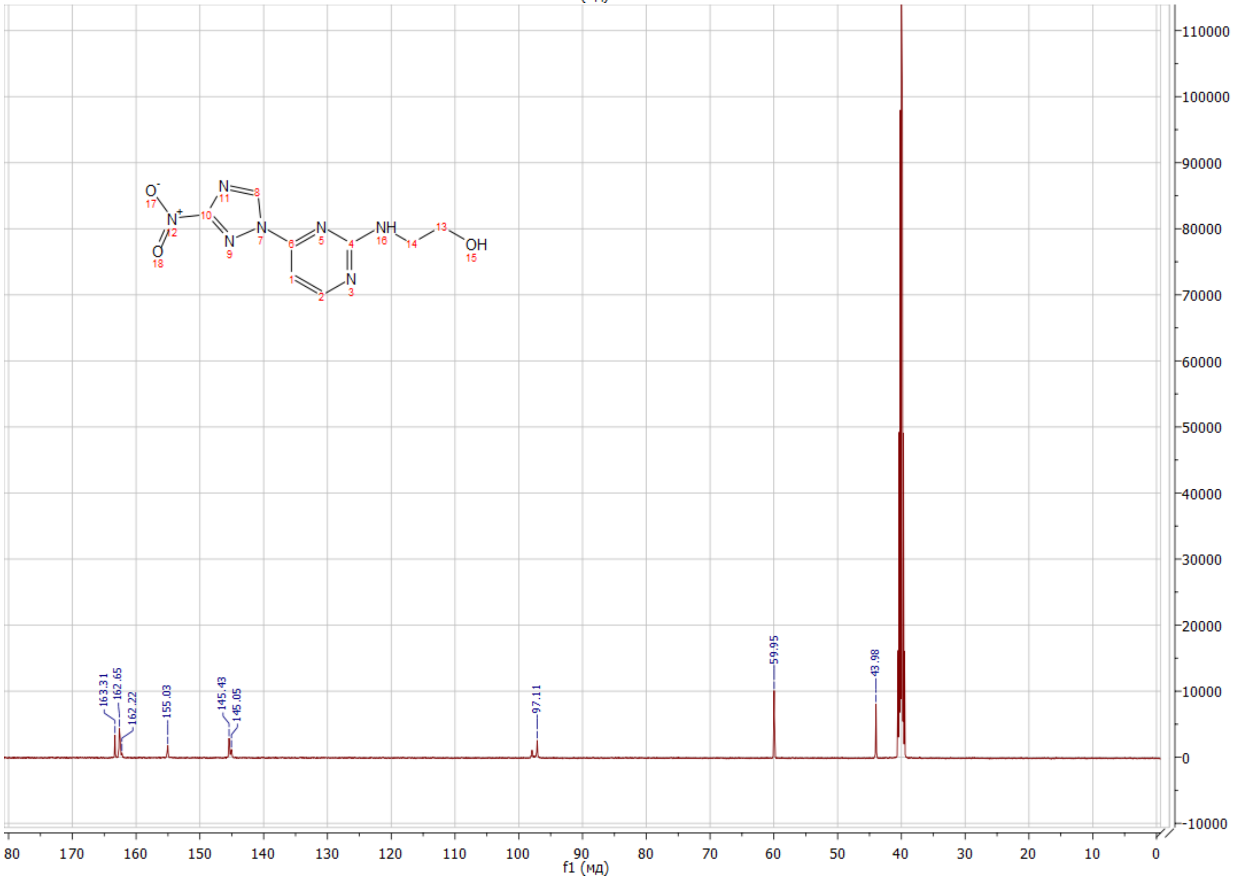

^{1}H and ^{13}C NMR of *N*,*N*-dimethyl-4-(3-nitro-1*H*-1,2,4-triazol-1-yl)pyrimidin-2-amine (2b)

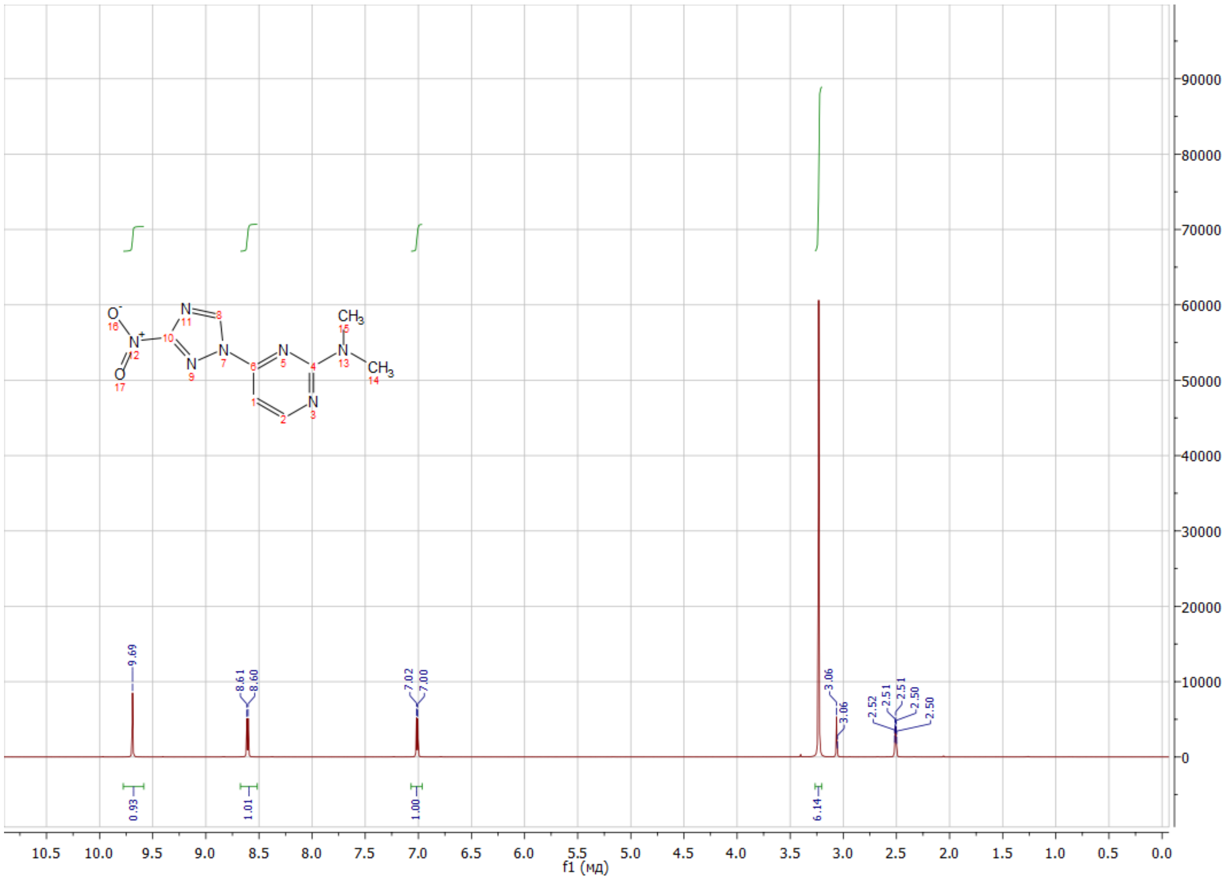

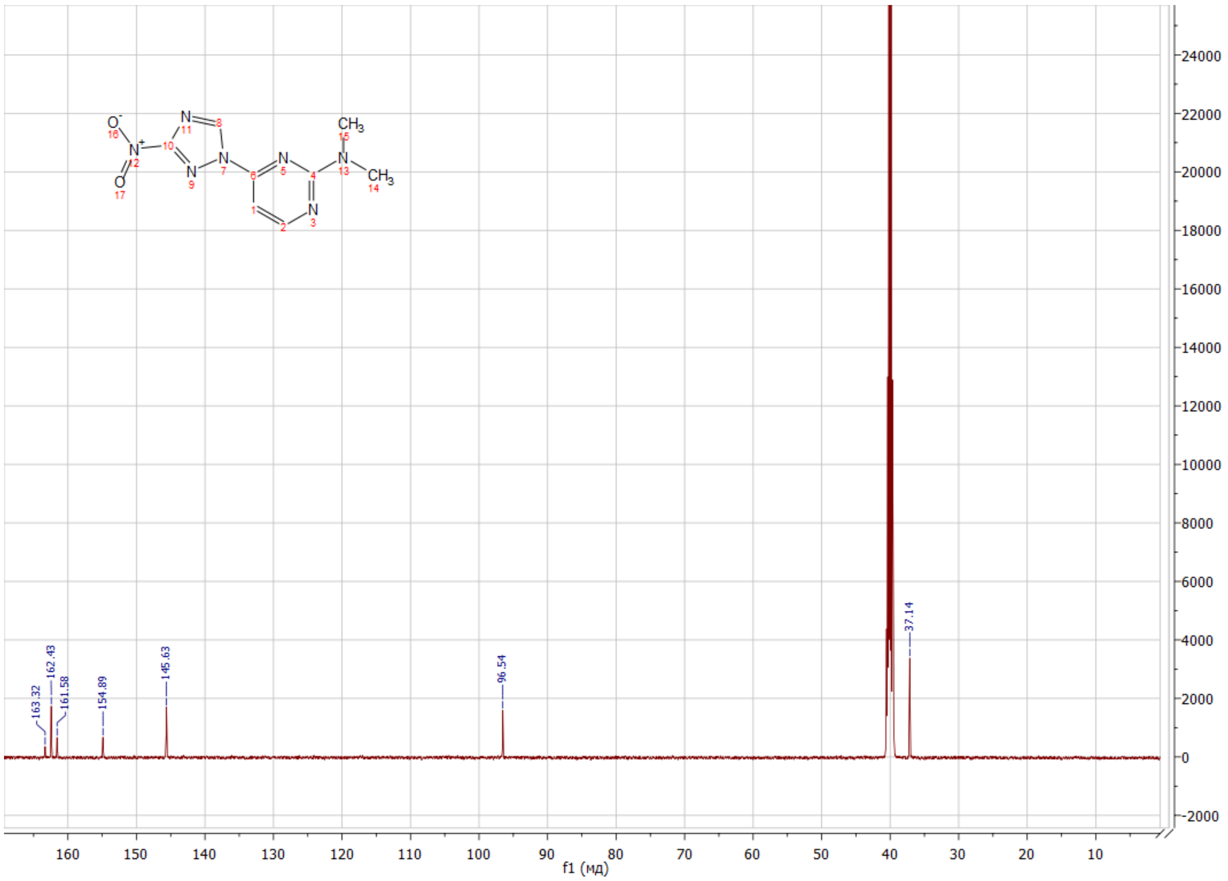

^{1}H and ^{13}C NMR of *N*-methyl-4-(3-nitro-1*H*-1,2,4-triazol-1-yl)pyrimidin-2-amine (2c)

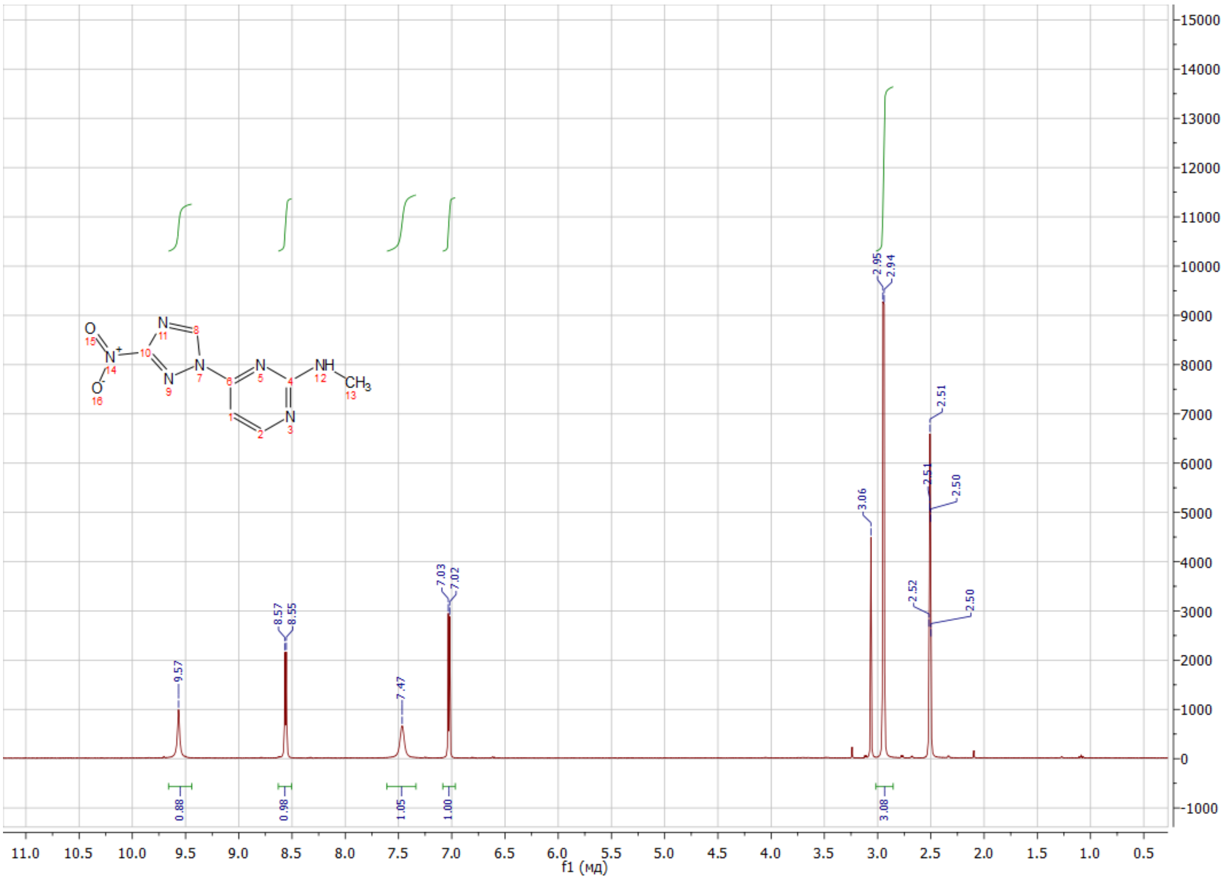

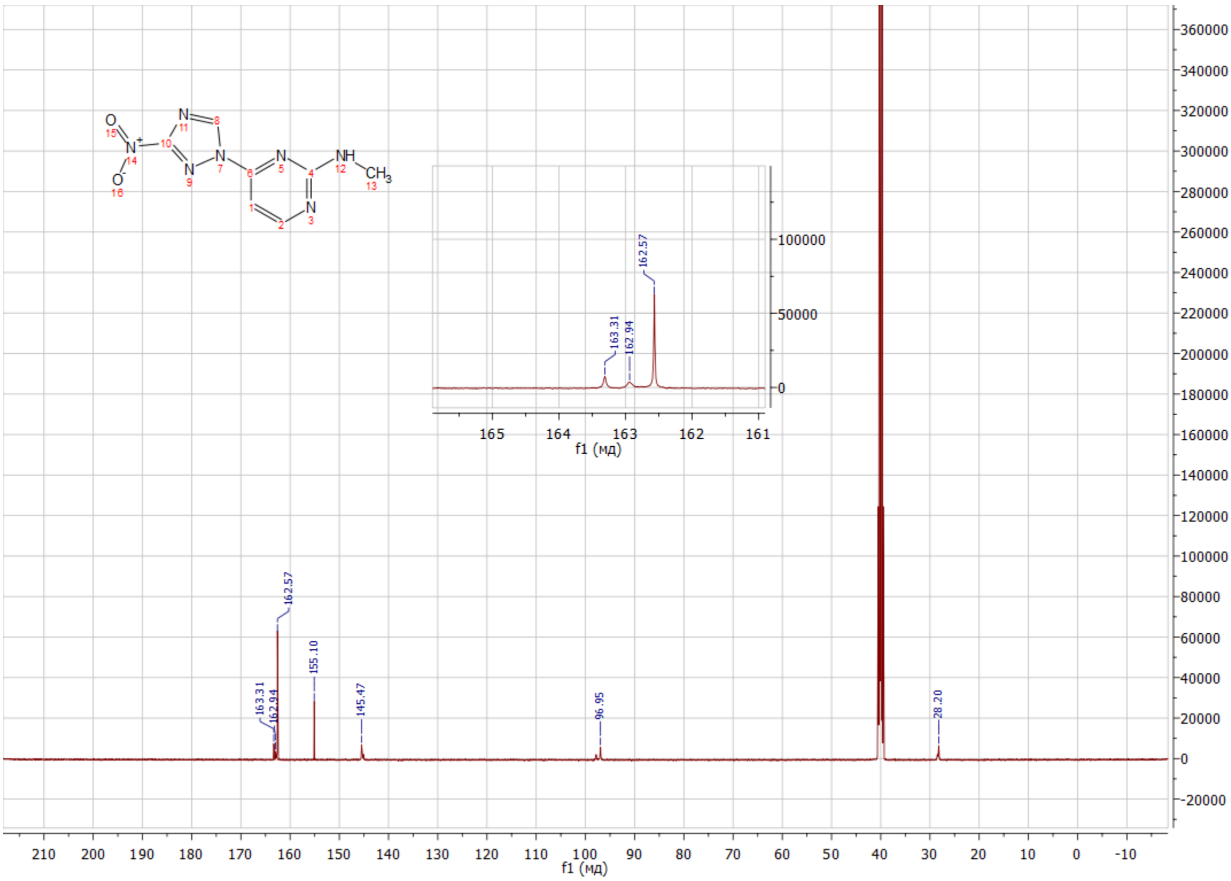

^{1}H and ^{13}C NMR of *N*-benzyl-*N*-methyl-4-(3-nitro-1*H*-1,2,4-triazol-1-yl)pyrimidin-2-amine (2d)

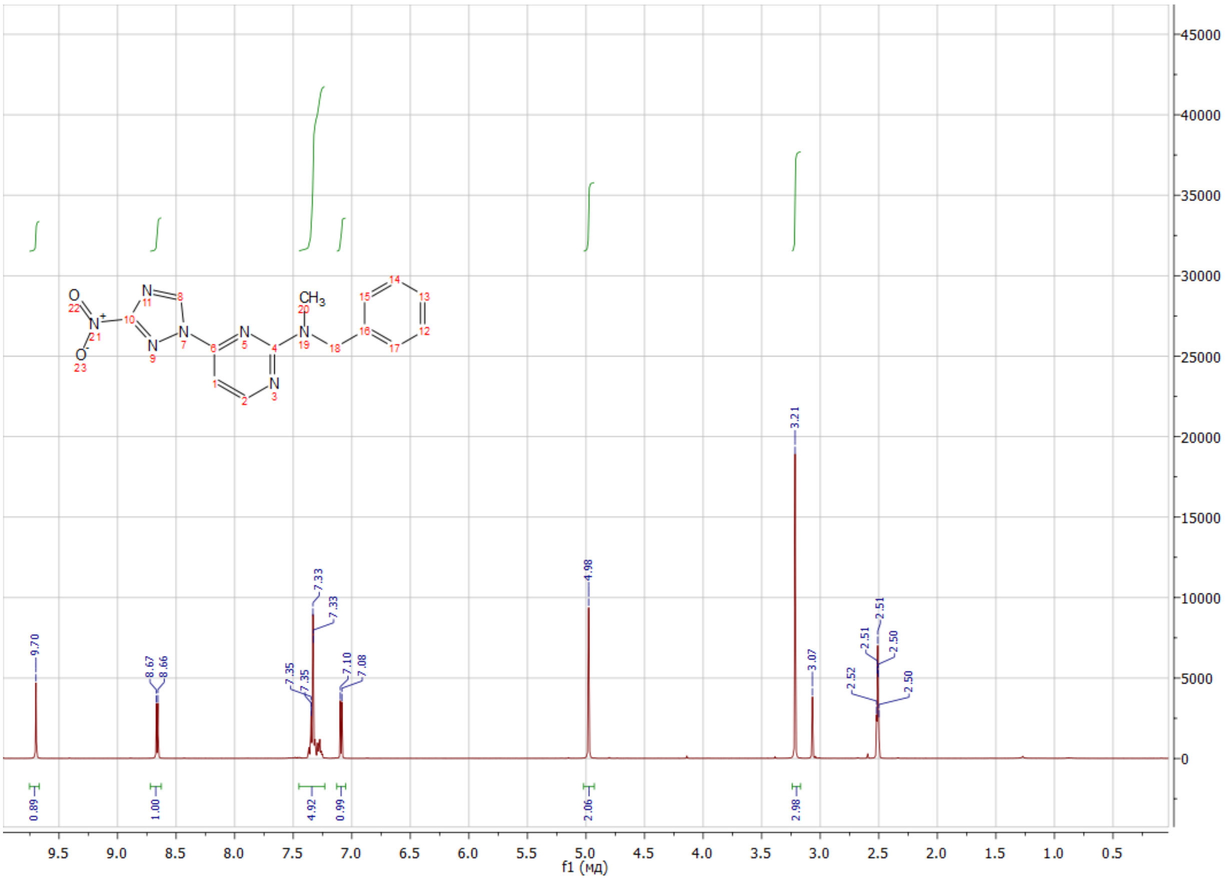

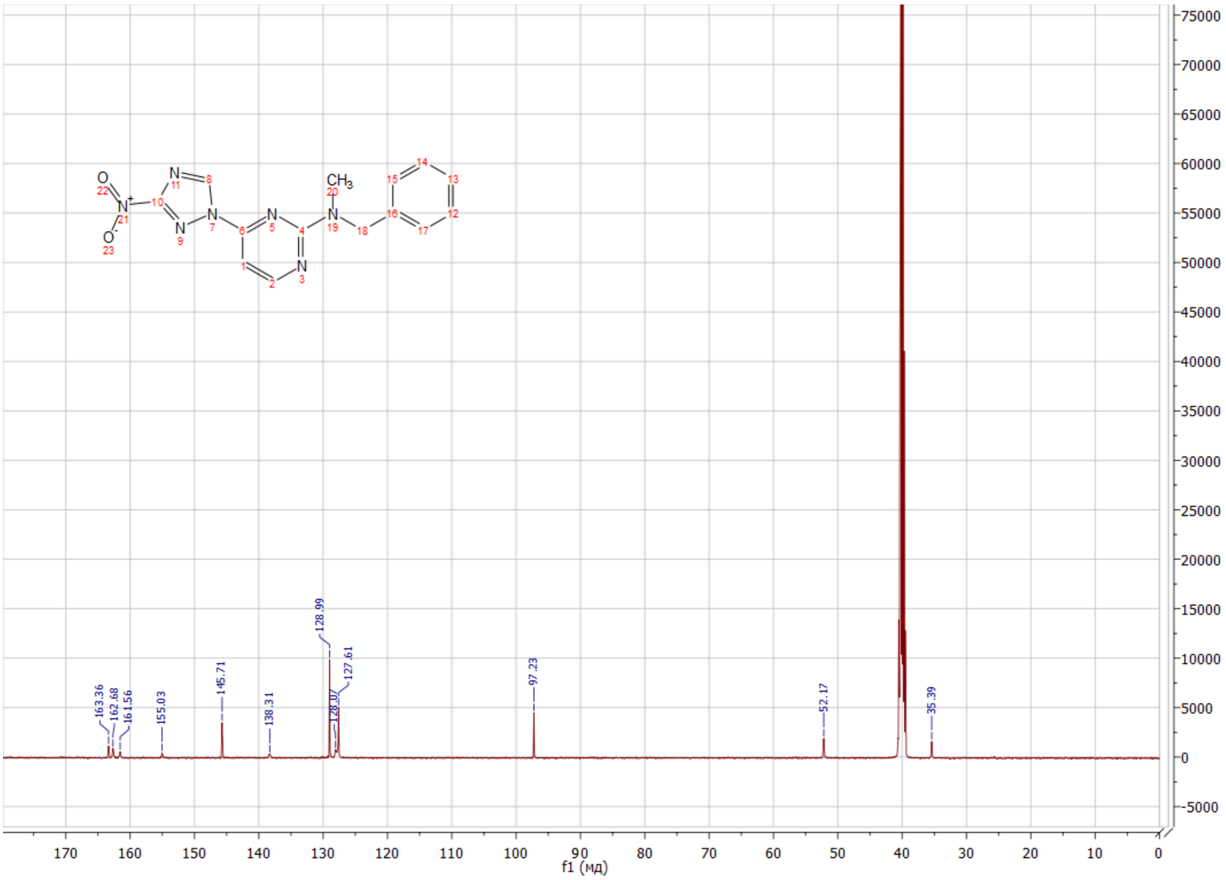

^{1}H and ^{13}C NMR of *N*-(4-methoxyphenyl)-4-(3-nitro-1*H*-1,2,4-triazol-1-yl)pyrimidin-2-amine (2e)

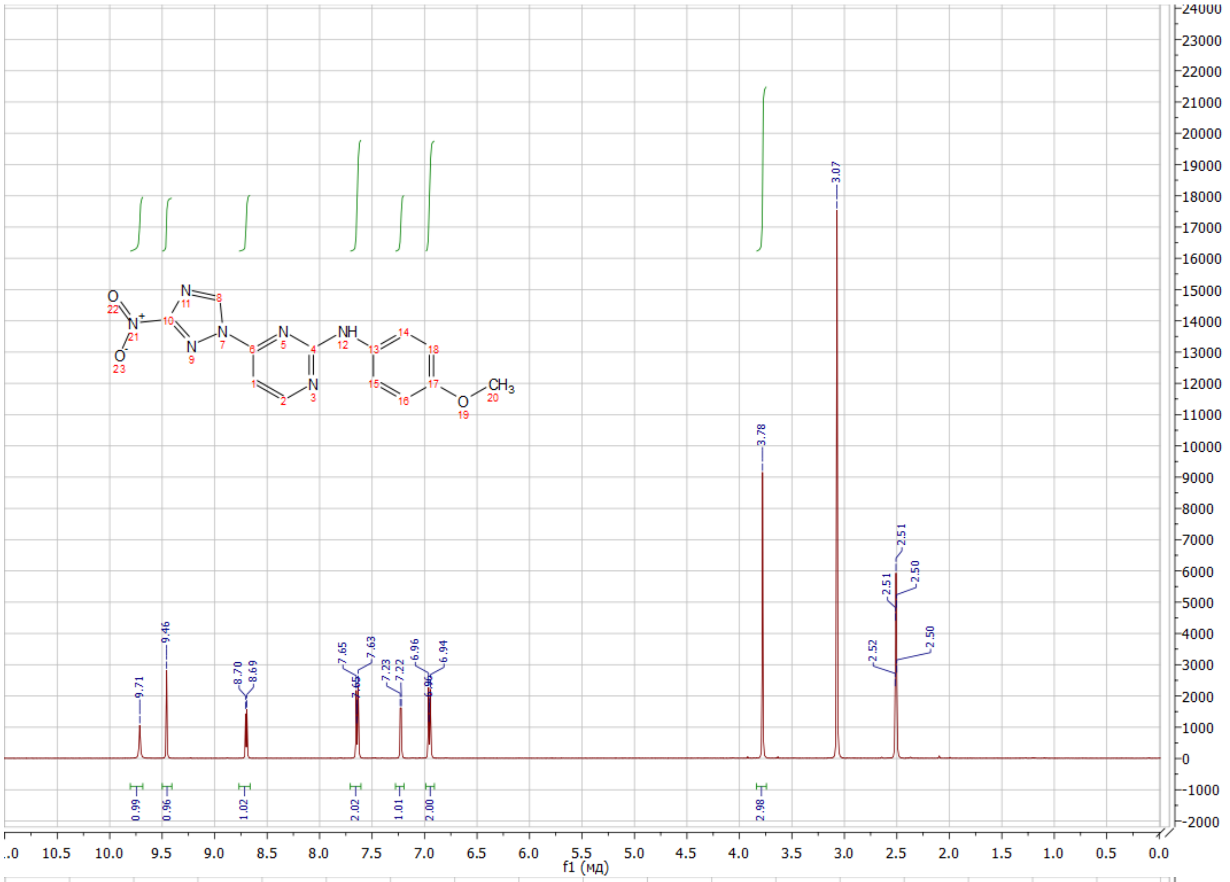

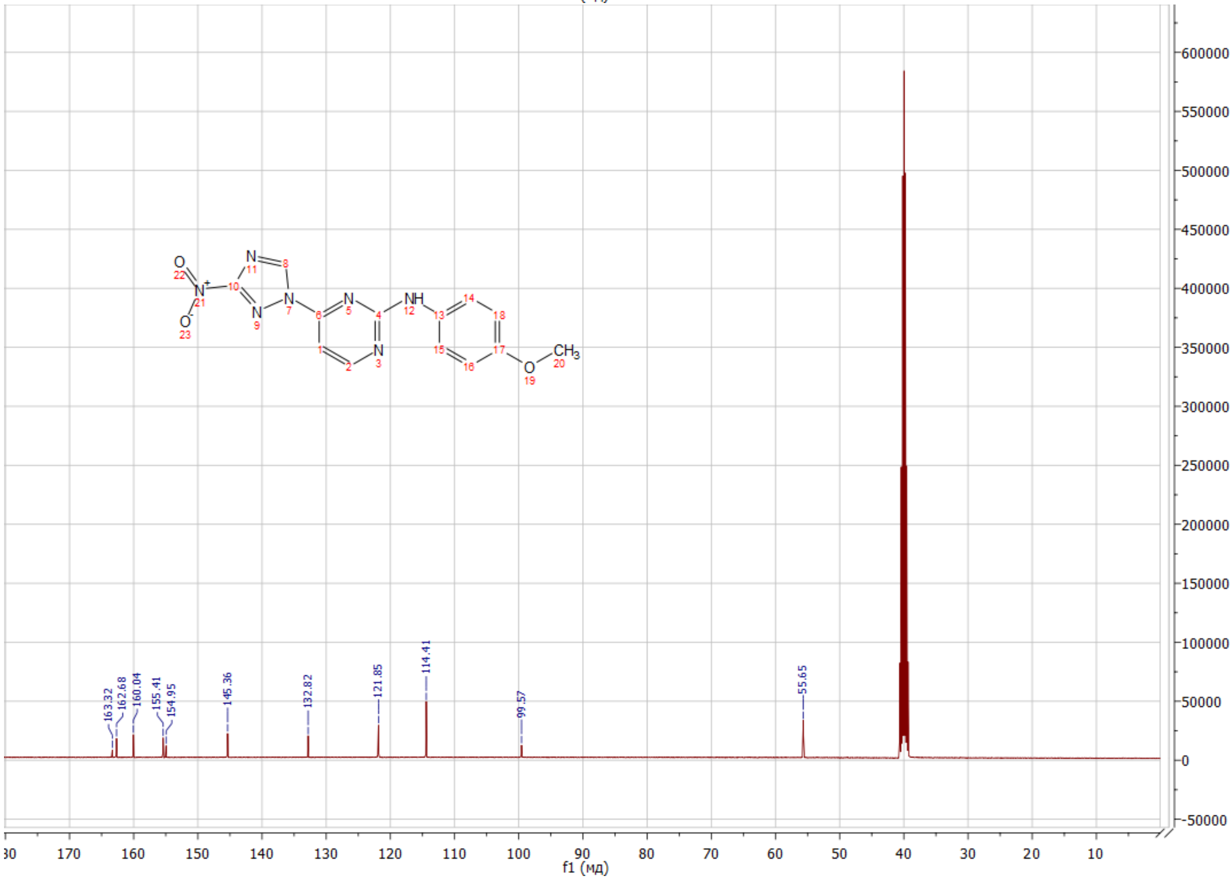

^{1}H and ^{13}C NMR of 3-((4-(3-nitro-1*H*-1,2,4-triazol-1-yl)pyrimidin-2-yl)amino)propan-1-ol (2f)

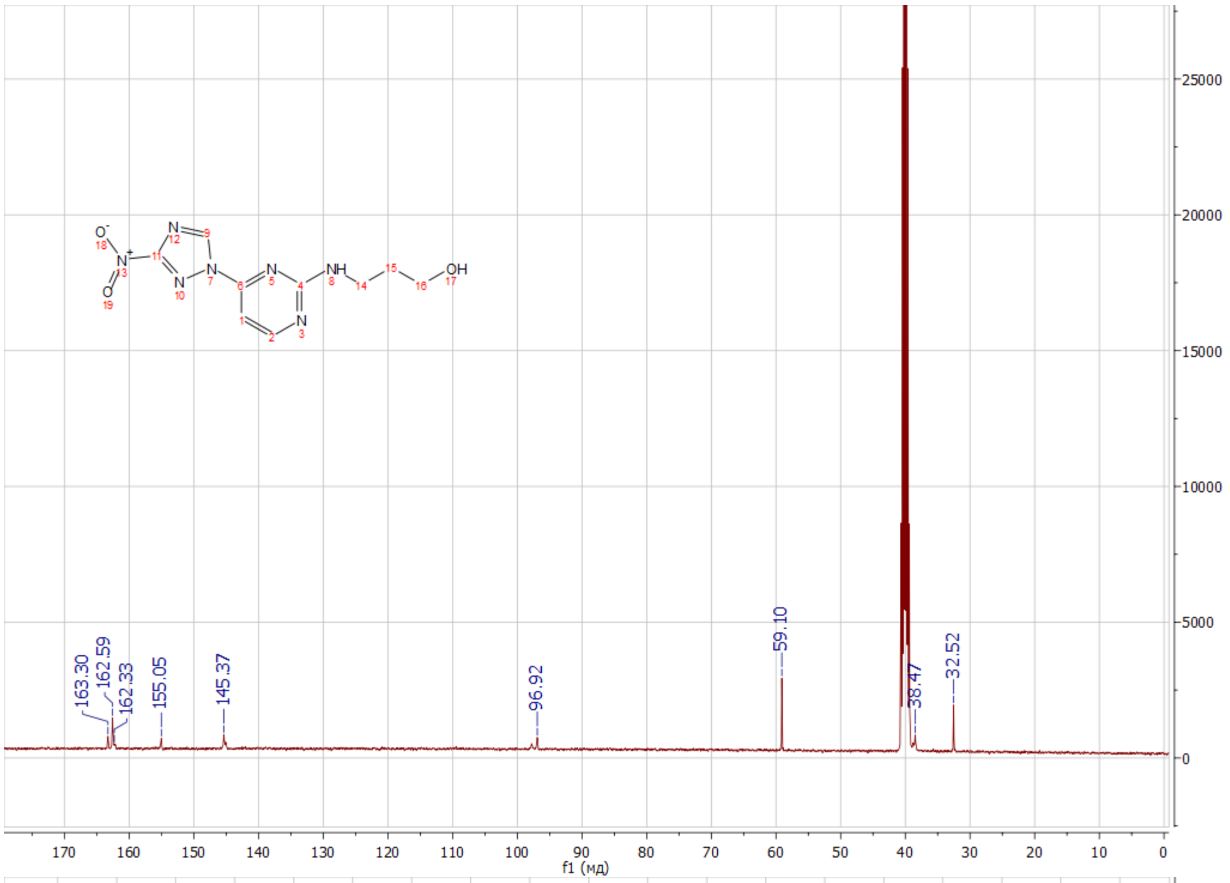

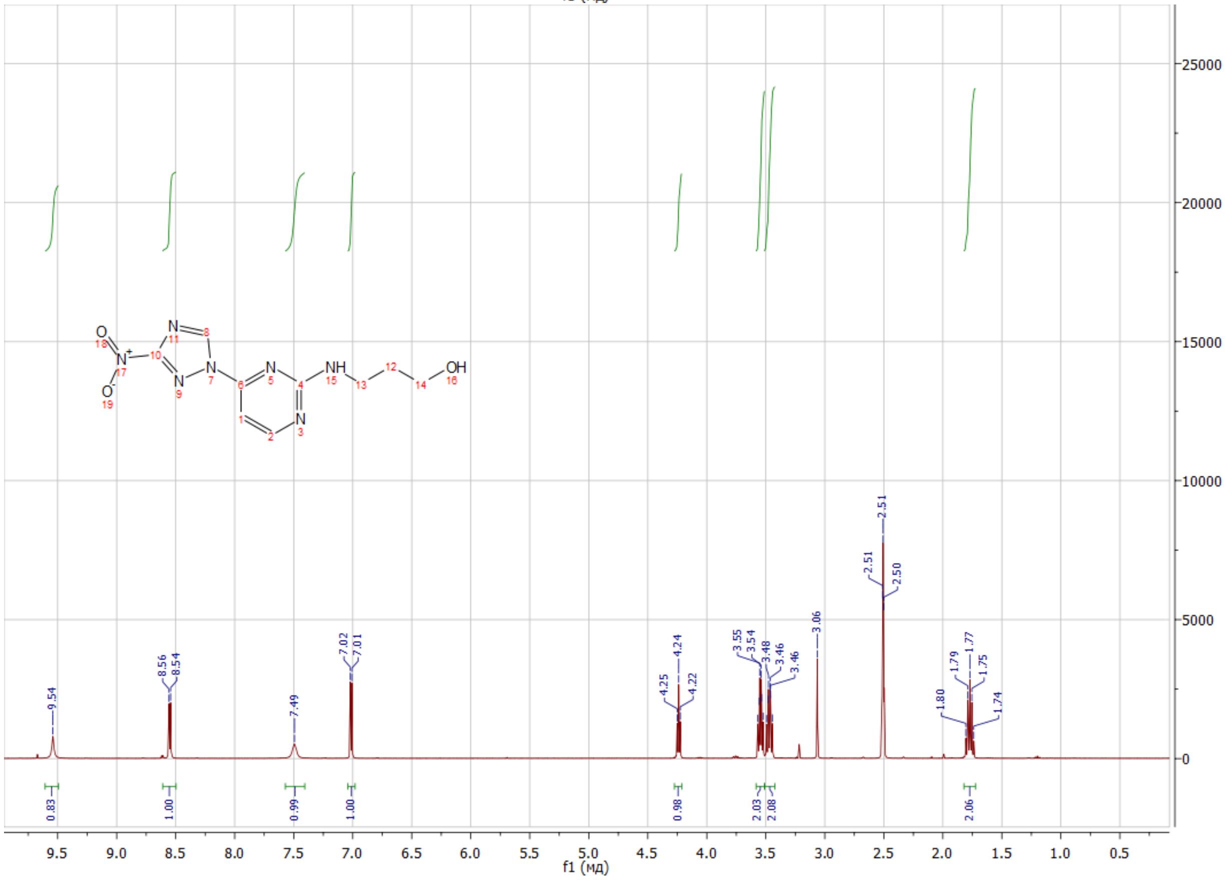

^{1}H and ^{13}C NMR of *N*-ethyl-4-(3-nitro-1*H*-1,2,4-triazol-1-yl)pyrimidin-2-amine (2g)

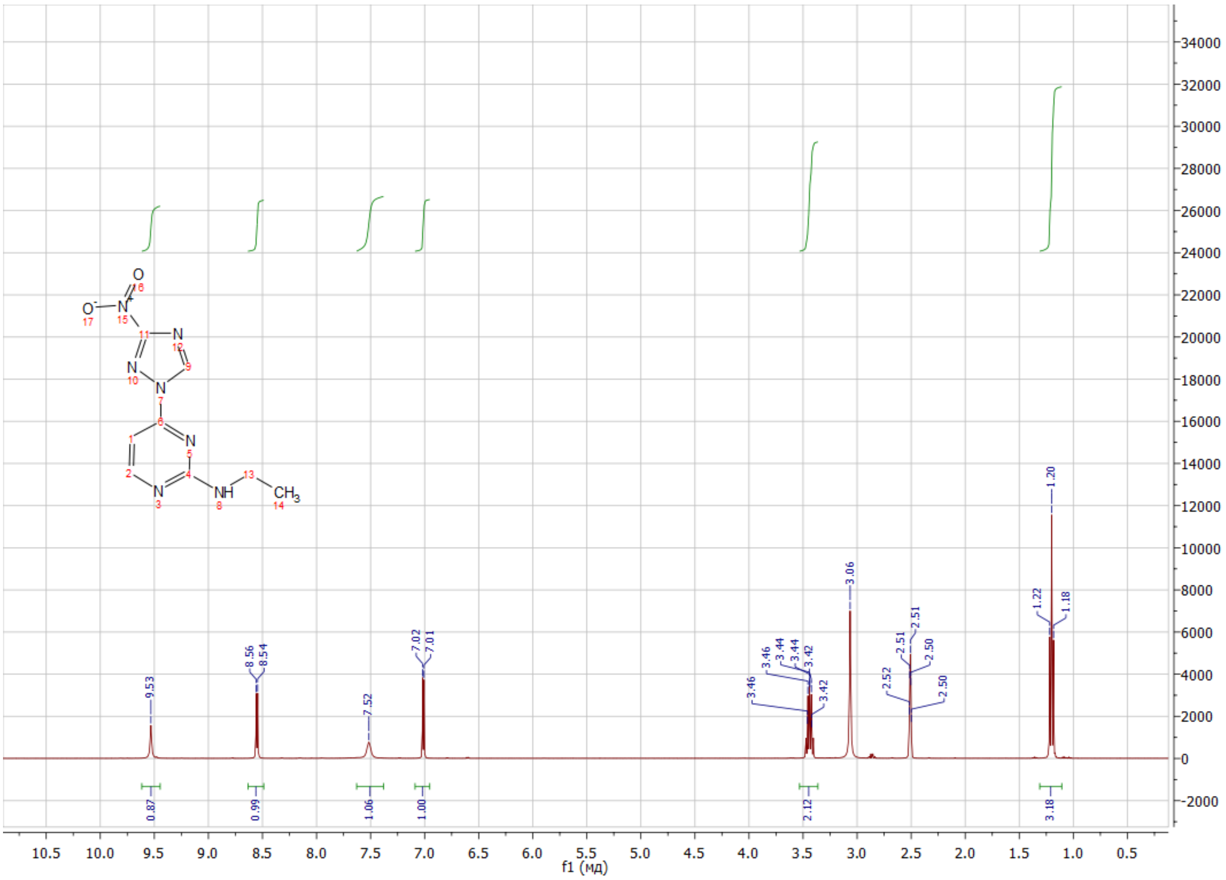

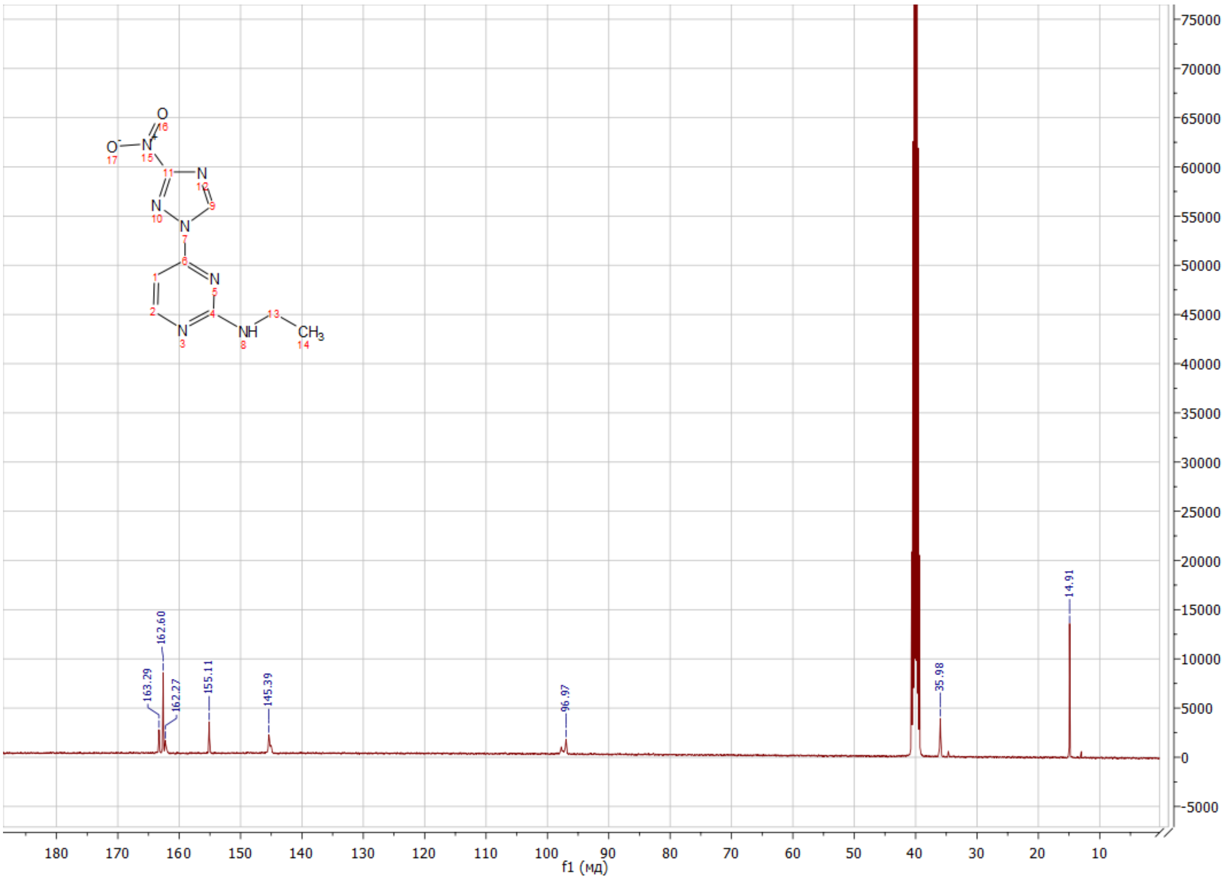

^{1}H and ^{13}C NMR of 4-(4-(3-nitro-1*H*-1,2,4-triazol-1-yl)pyrimidin-2-yl)morpholine (2h)

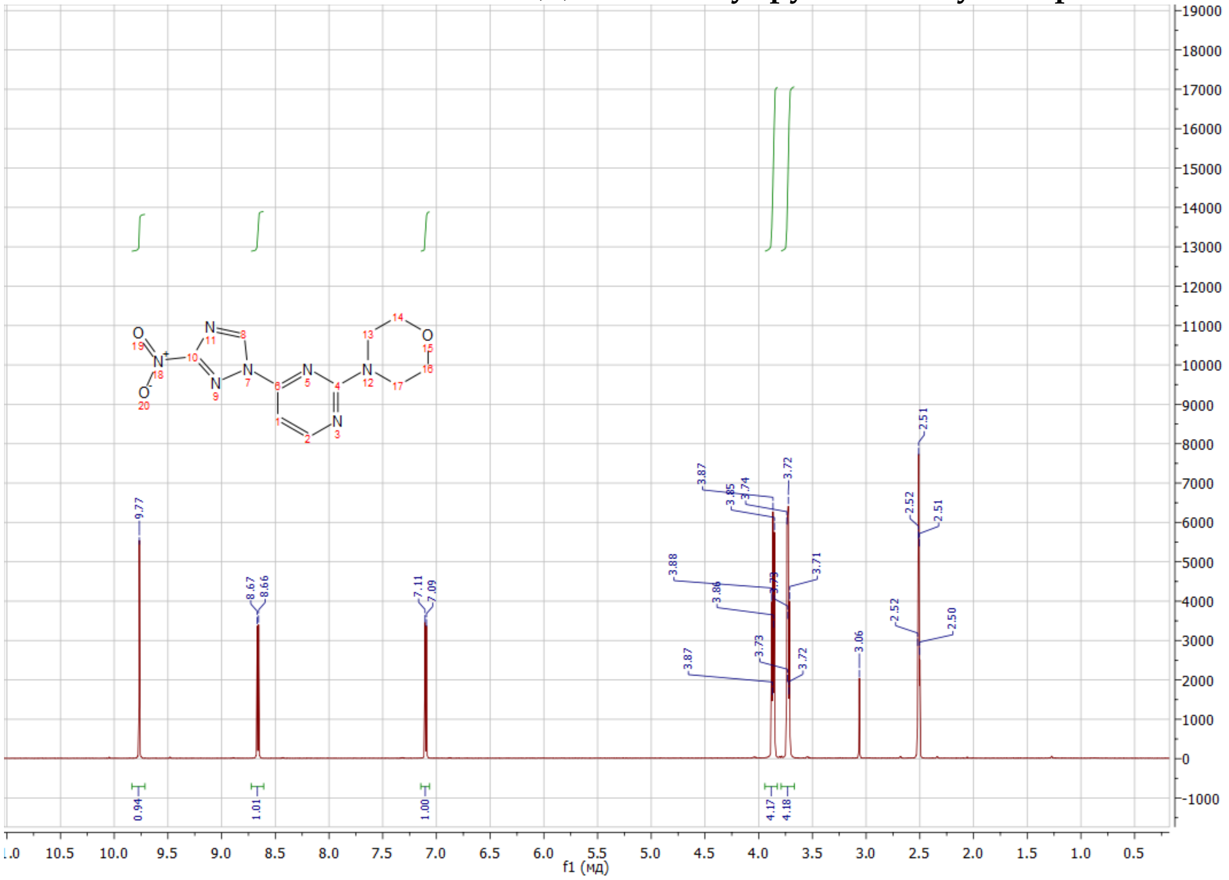

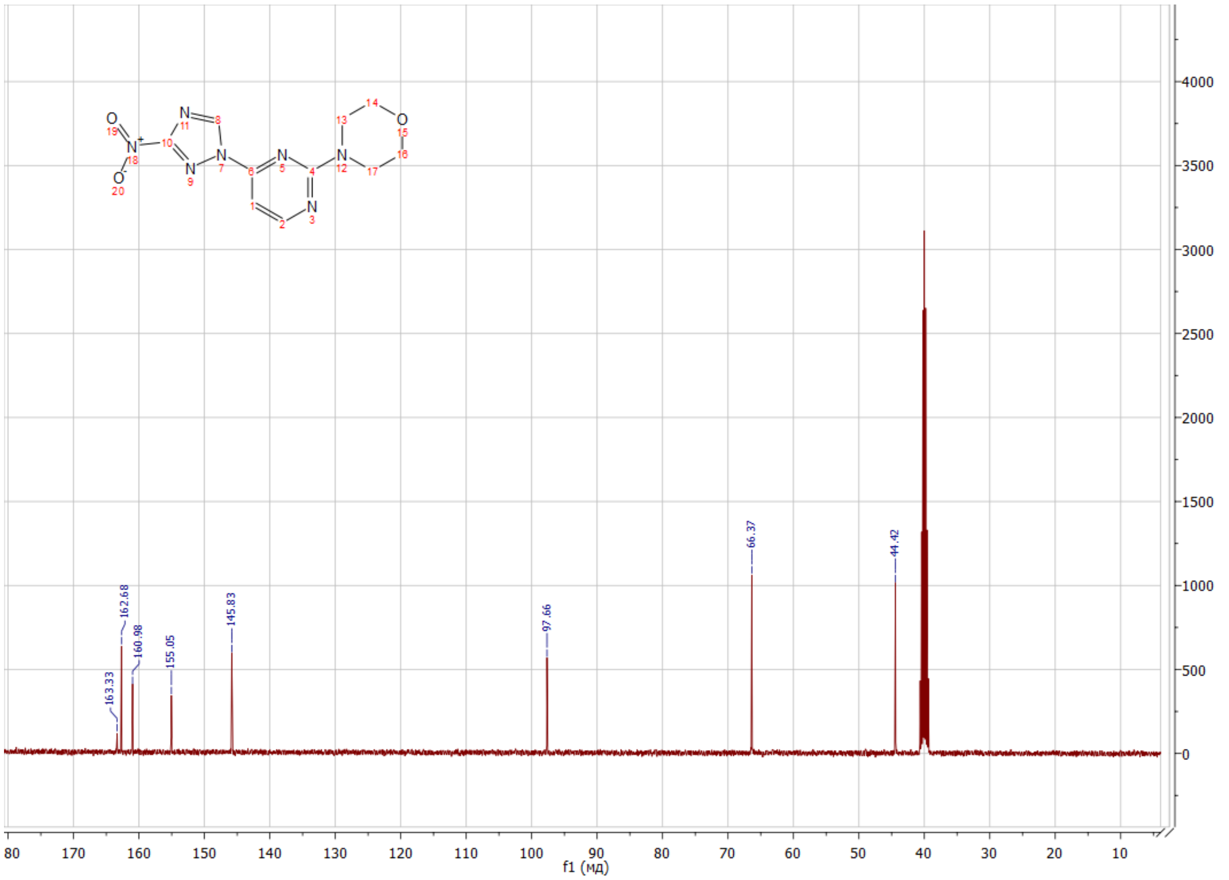

^{1}H and ^{13}C NMR of 2-(4-methylpiperazin-1-yl)-4-(3-nitro-1*H*-1,2,4-triazol-1-yl)pyrimidine (2i)

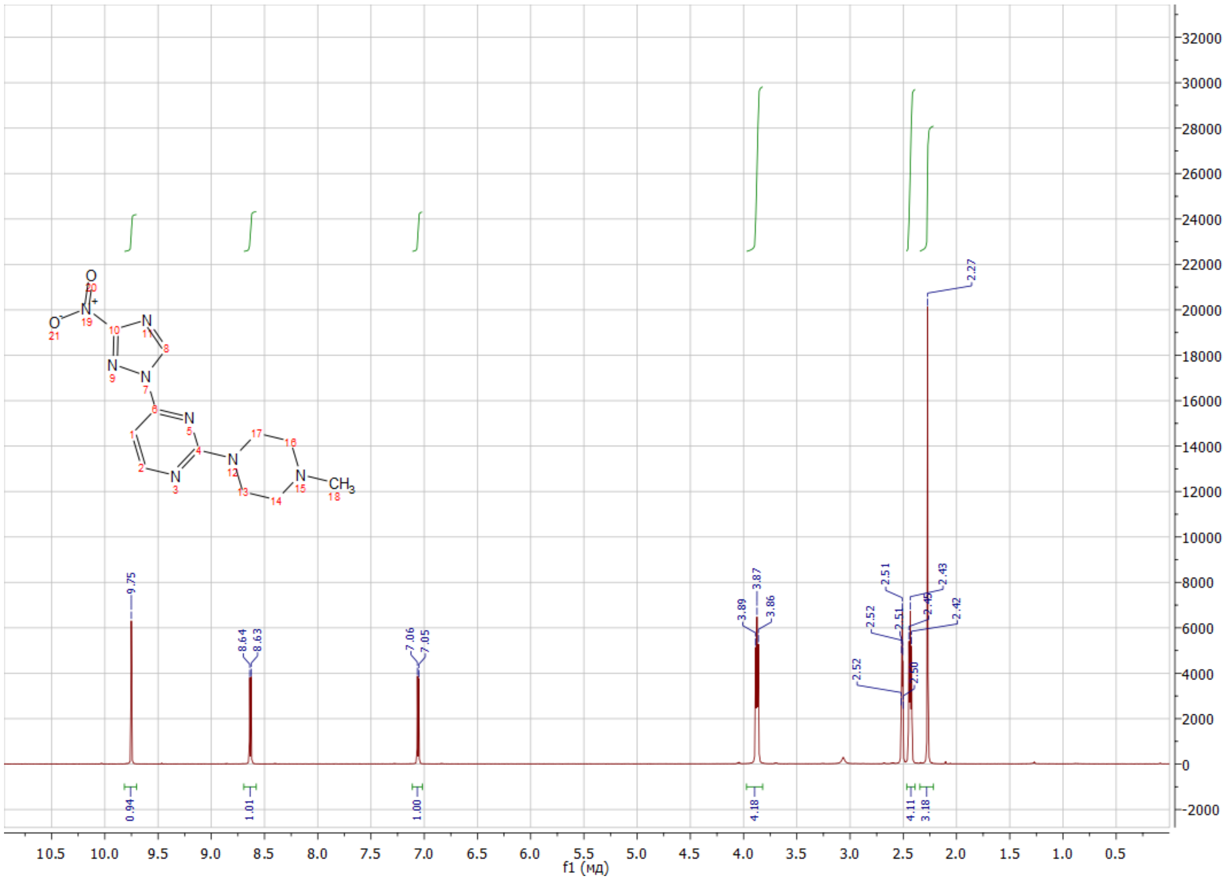

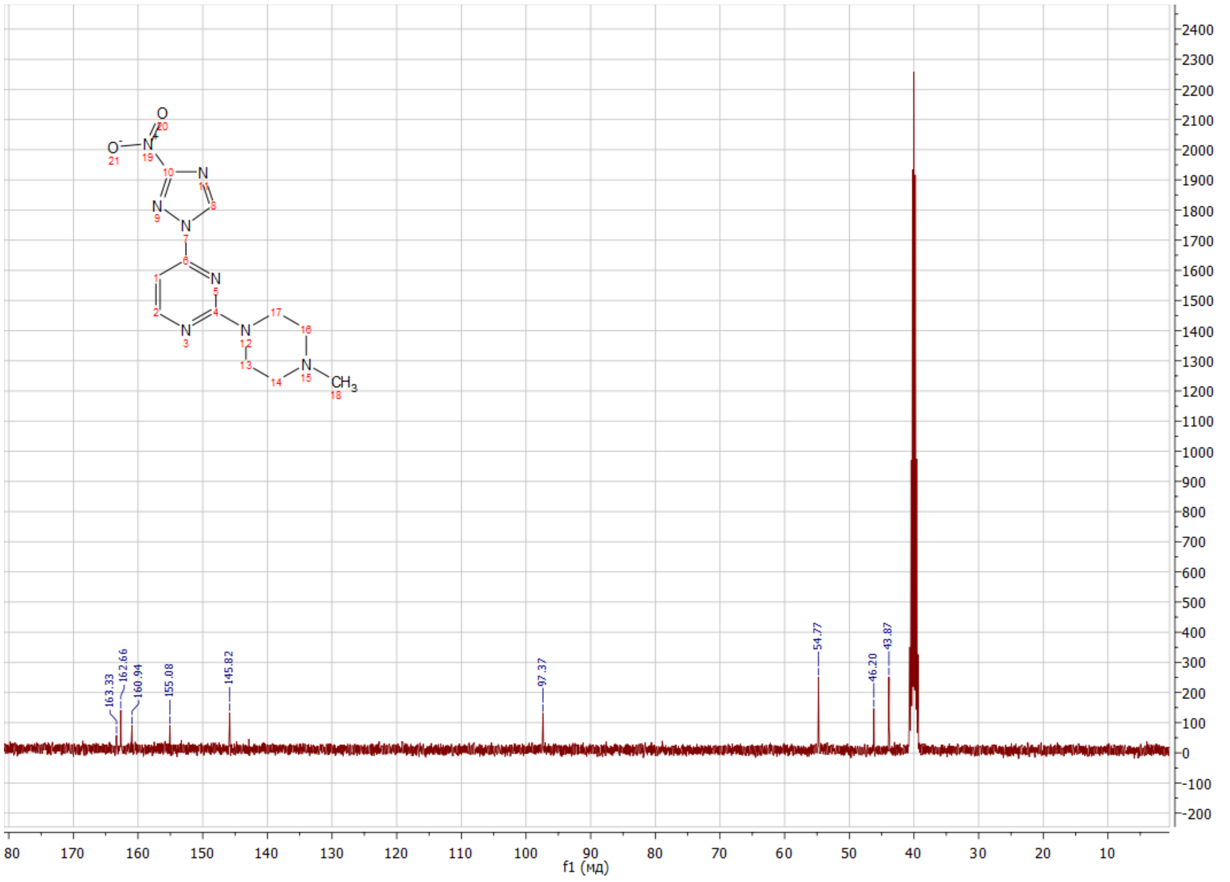

^{1}H and ^{13}C NMR of *N*-benzyl-4-(3-nitro-1*H*-1,2,4-triazol-1-yl)pyrimidin-2-amine (2j)

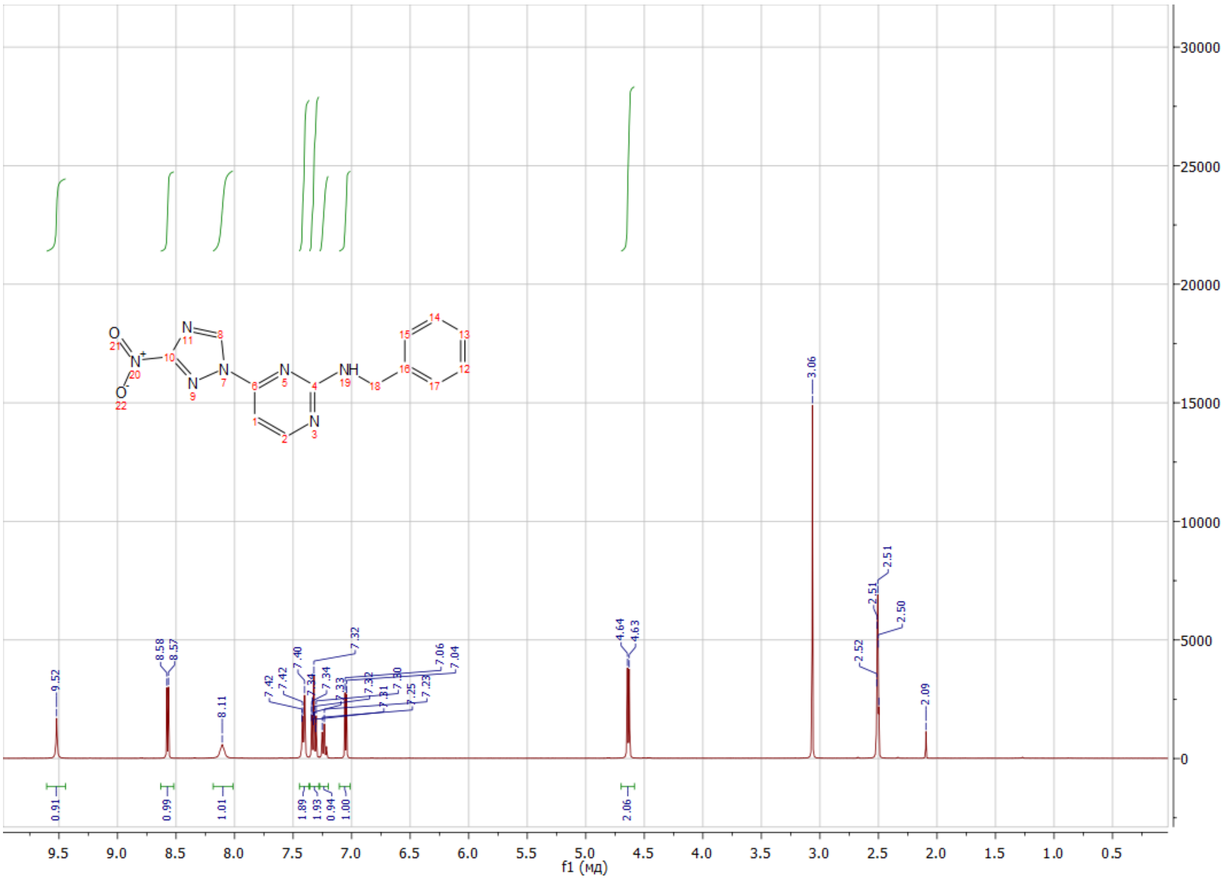

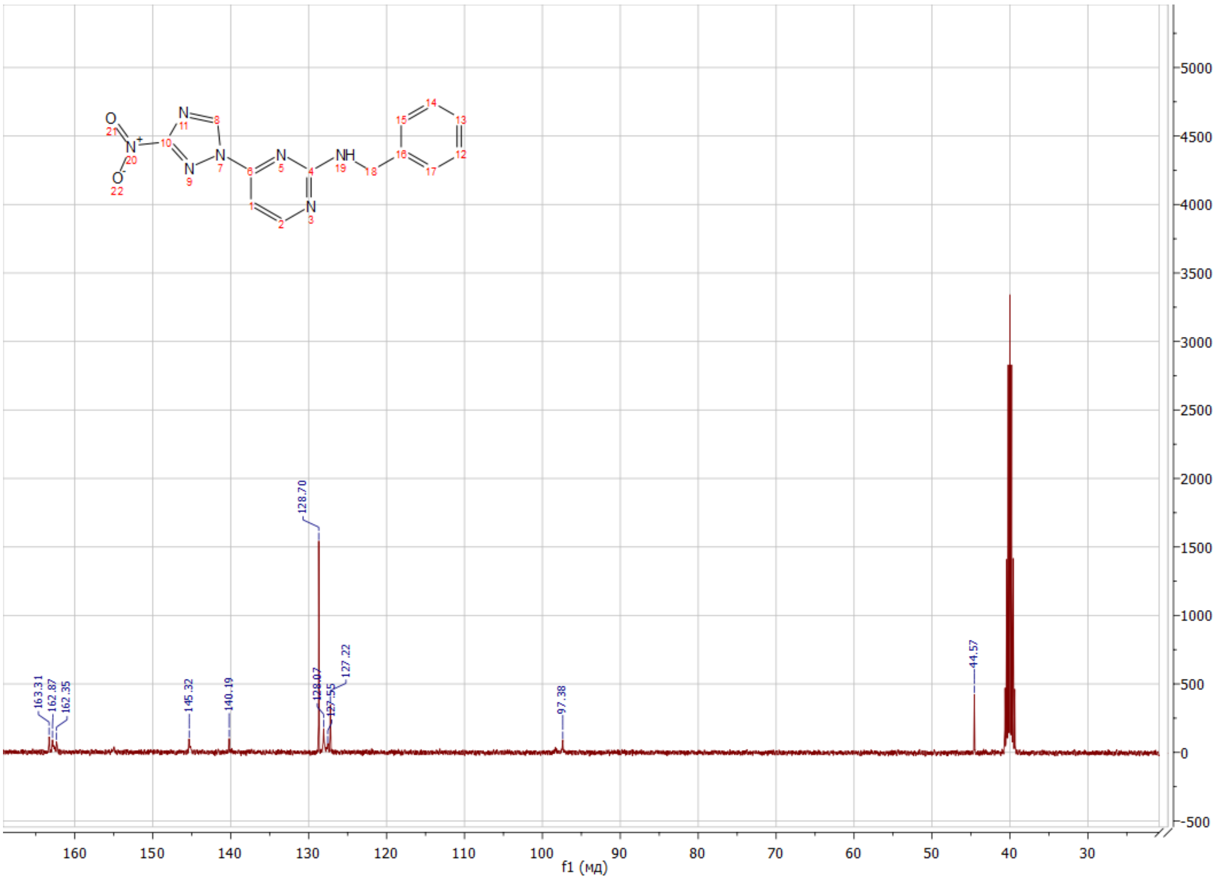

^{1}H and ^{13}C NMR of *N*-Isobutyl-4-(3-nitro-1*H*-1,2,4-triazol-1-yl)pyrimidin-2-amine (2k)

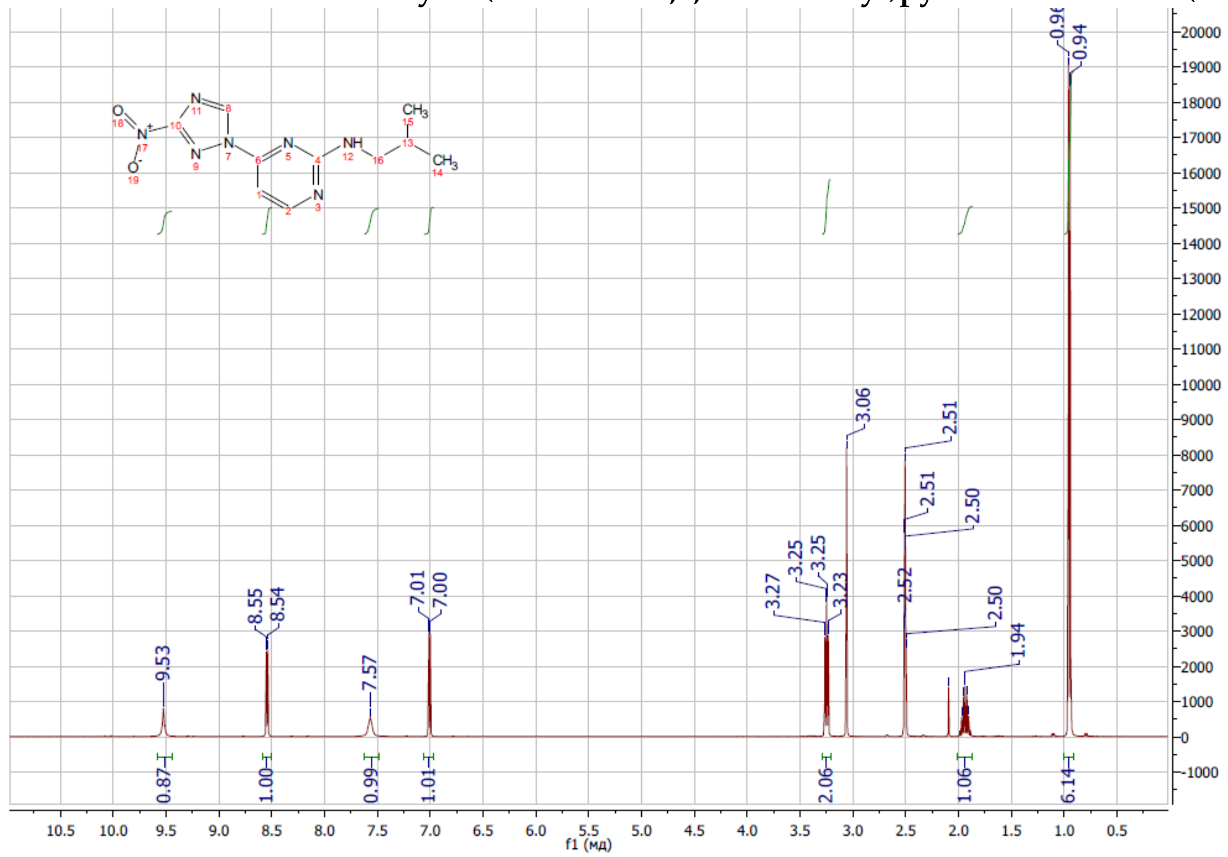

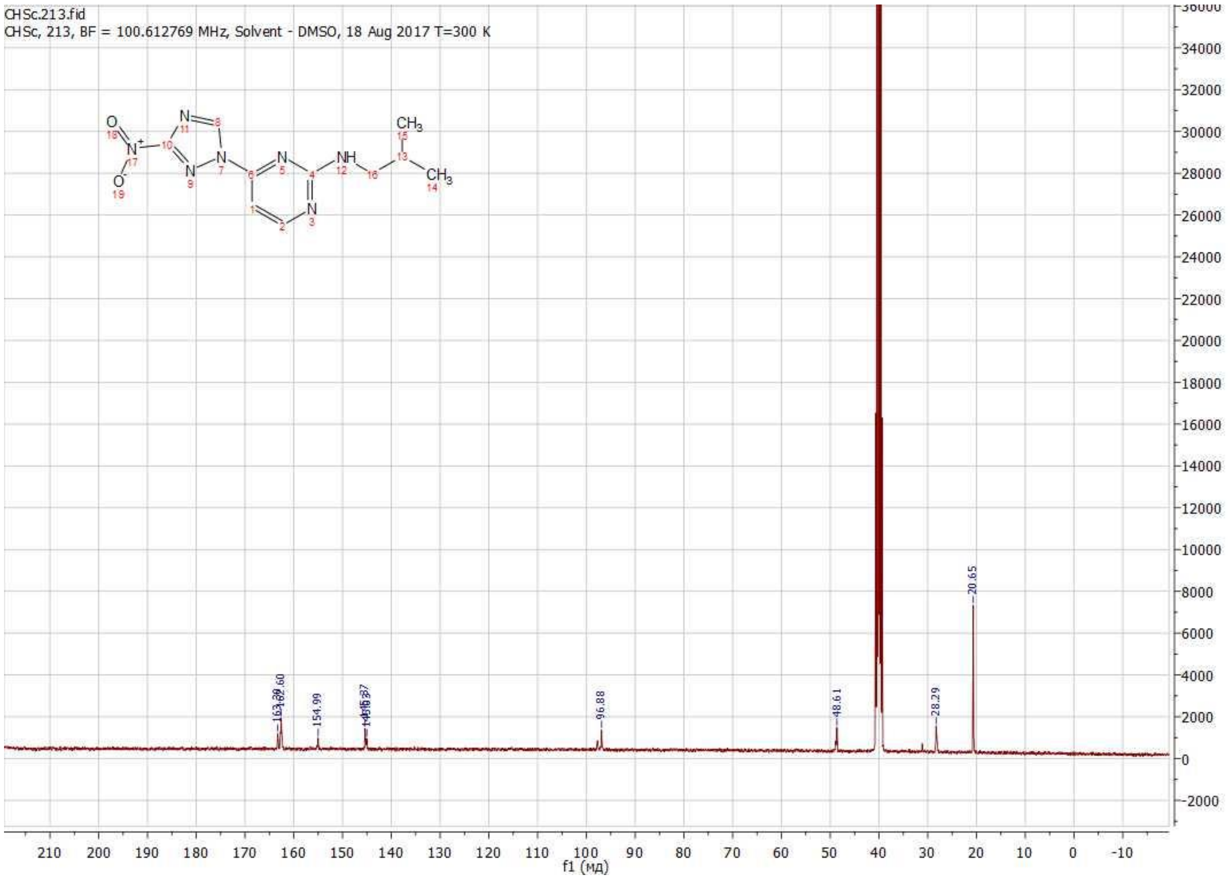

^{1}H and ^{13}C NMR of 2,2'-((4-(3-nitro-1*H*-1,2,4-triazol-1-yl)pyrimidin-2-yl)azanediyl)bis(ethan-1-ol) (2l)

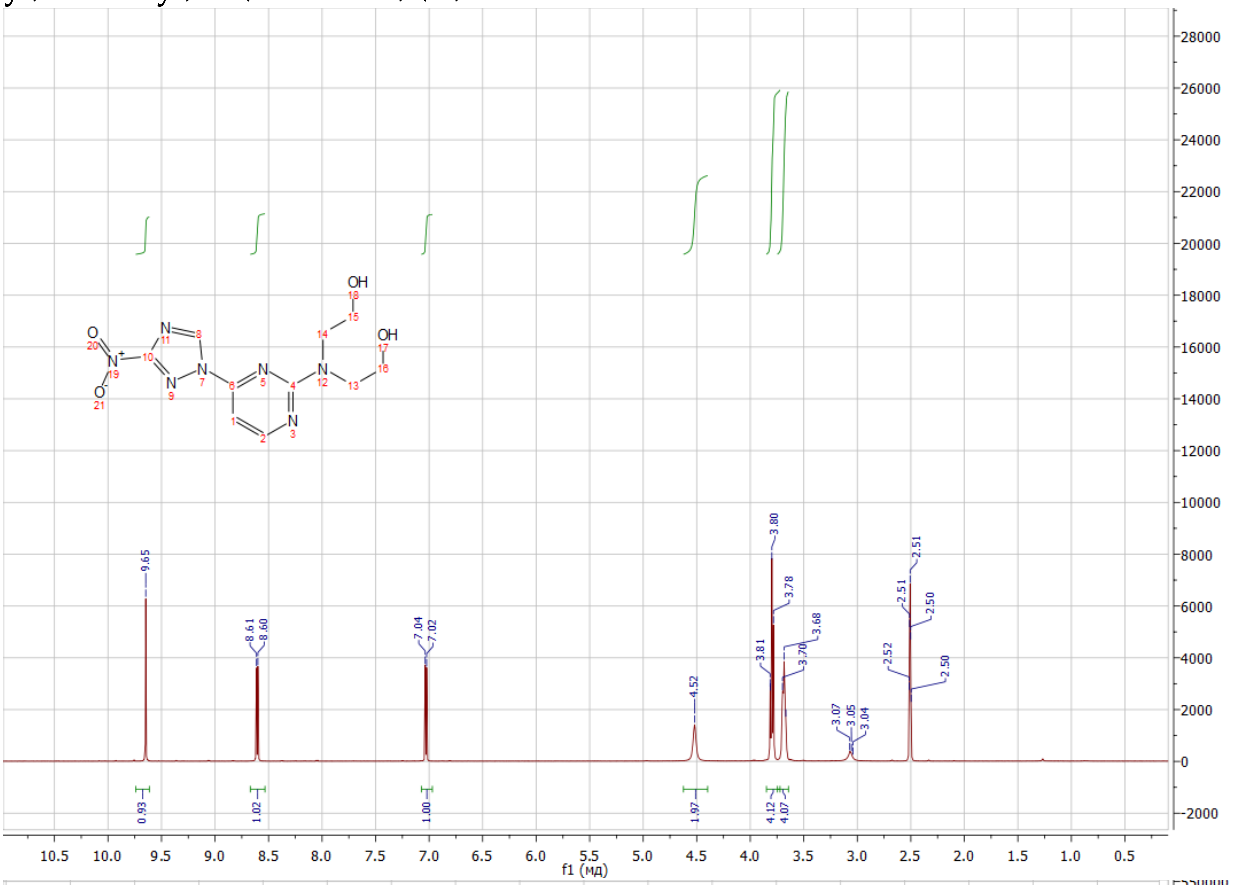

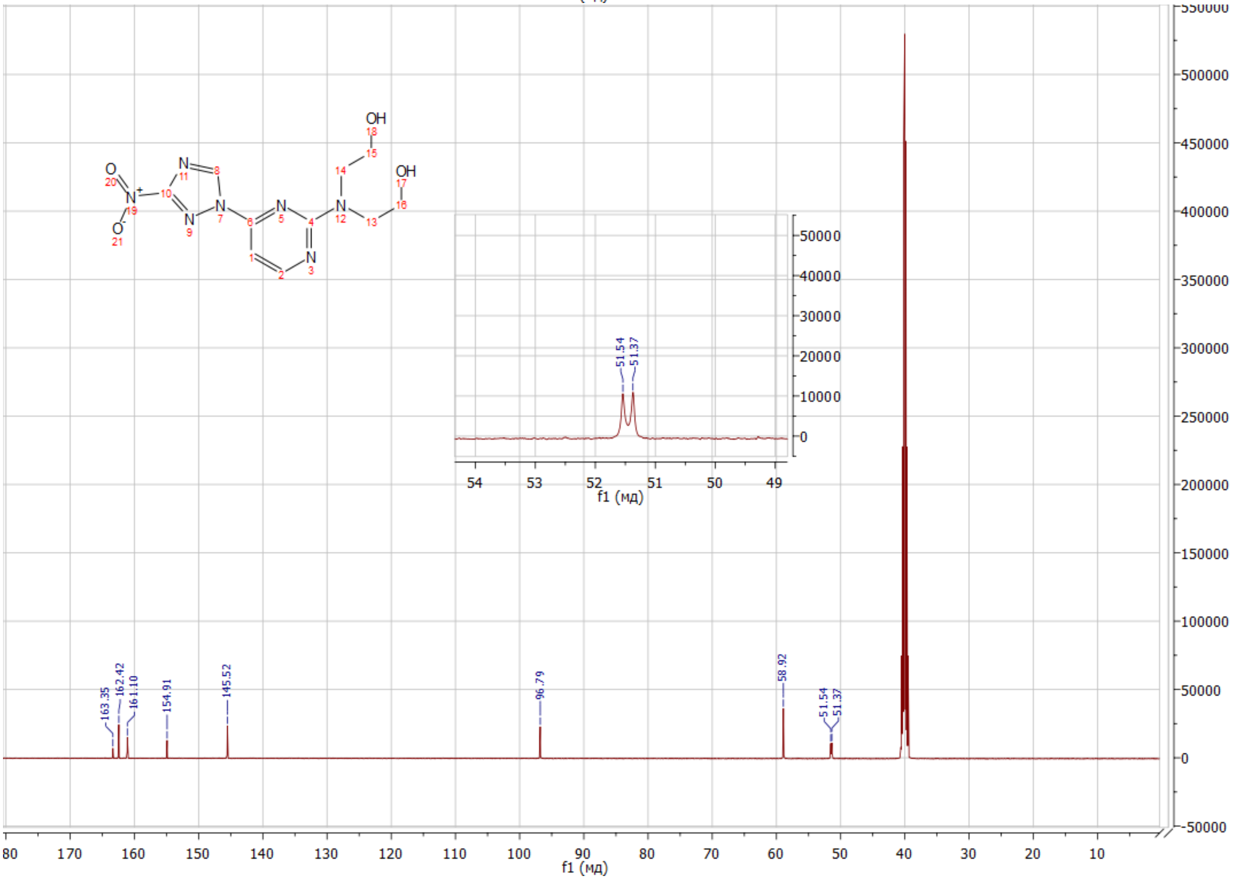

^{1}H and ^{13}C NMR of 4-(3-nitro-1*H*-1,2,4-triazol-1-yl)-*N*-(p-tolyl)pyrimidin-2-amine (2m)

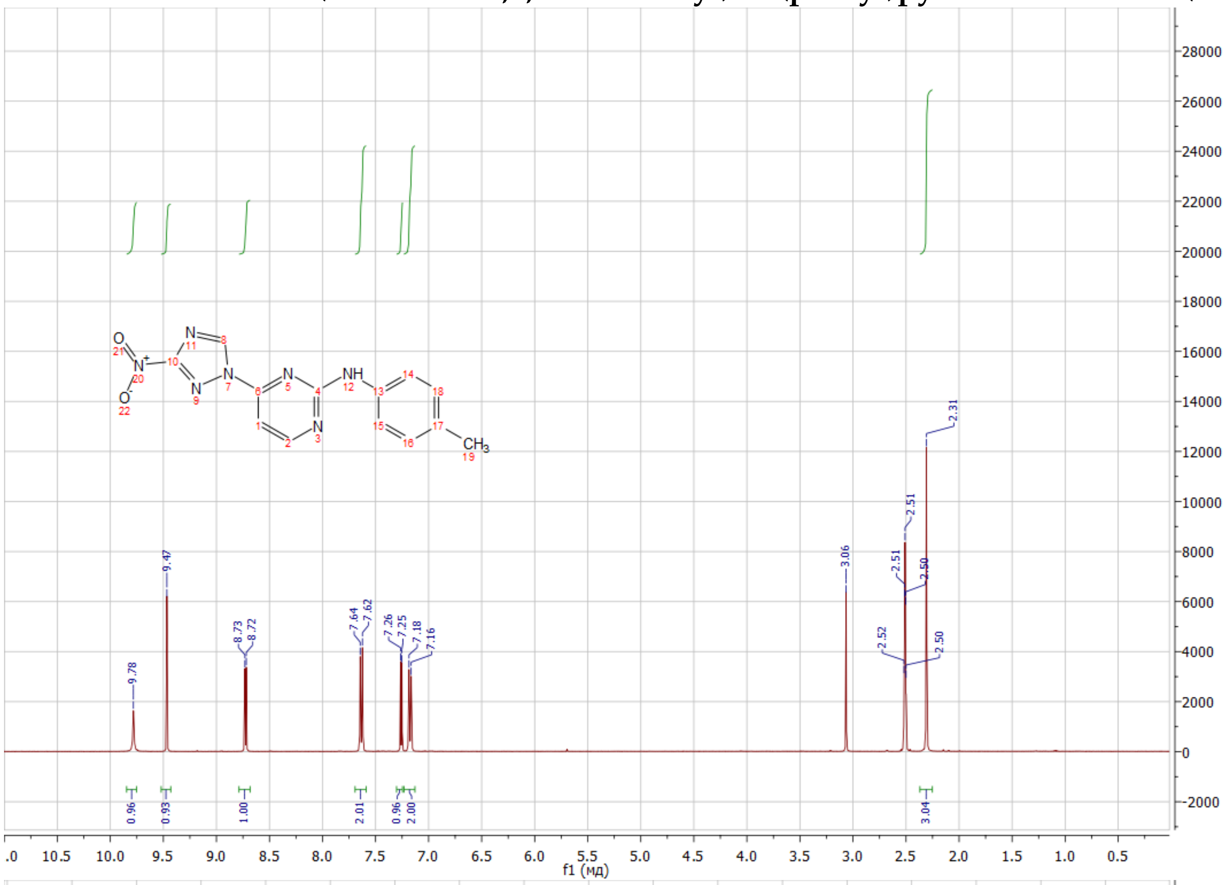

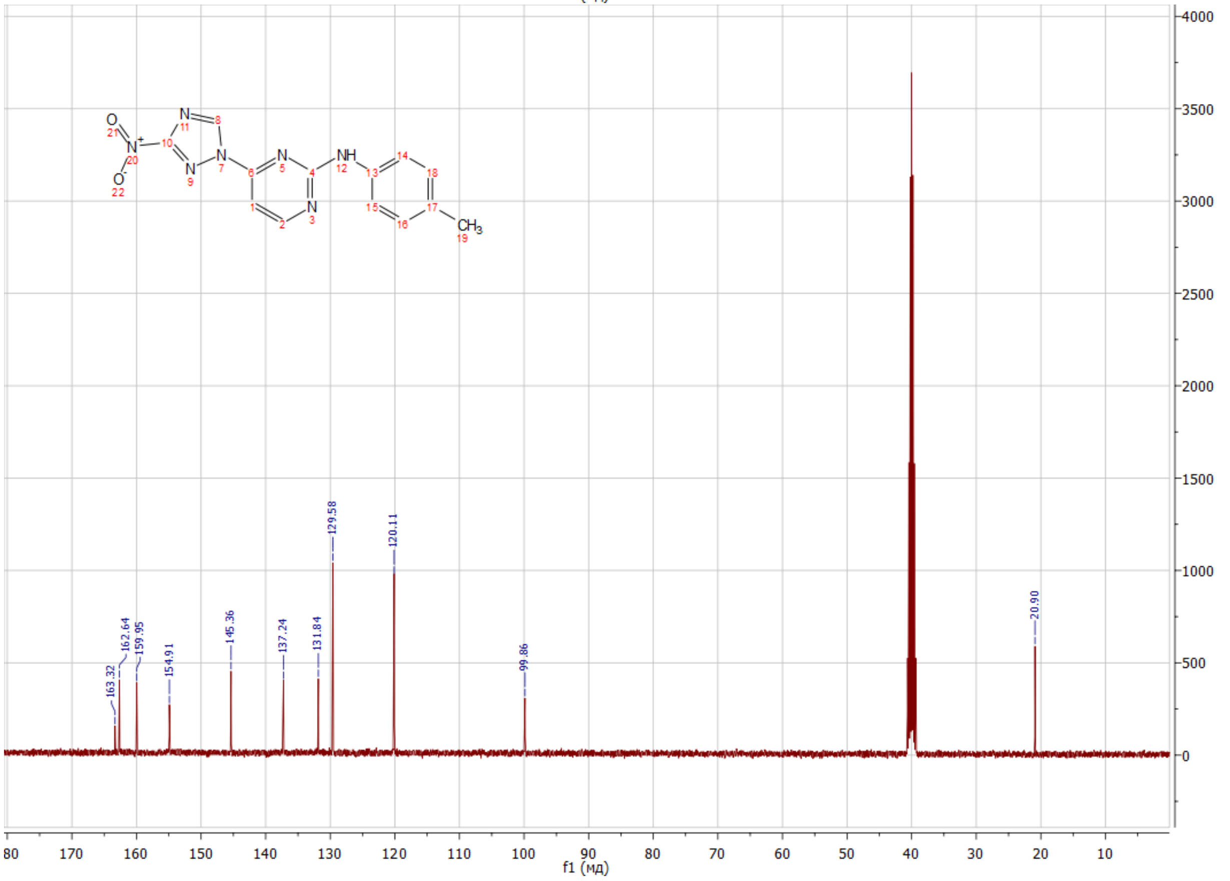

^{1}H and ^{13}C NMR of *N*-cyclopropyl-4-(3-nitro-1*H*-1,2,4-triazol-1-yl)pyrimidin-2-amine (2n)

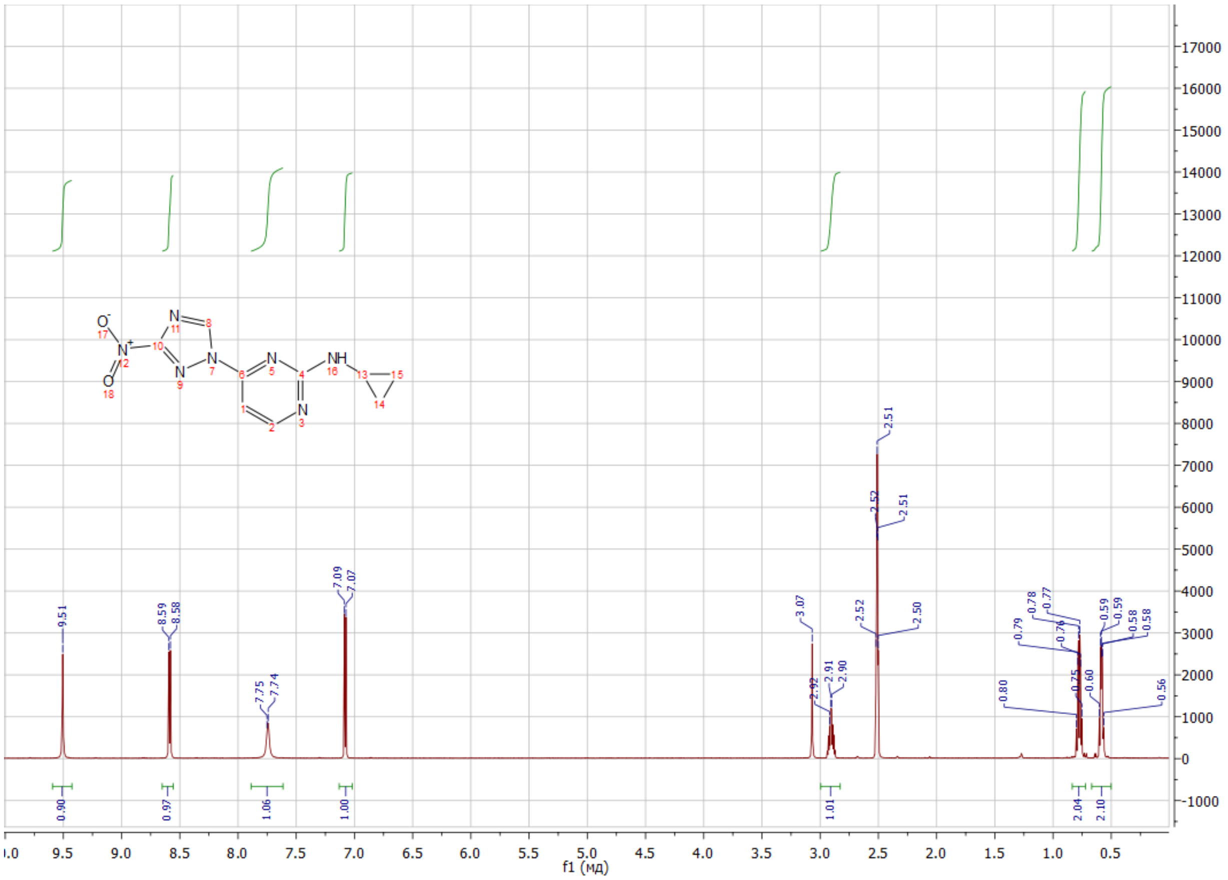

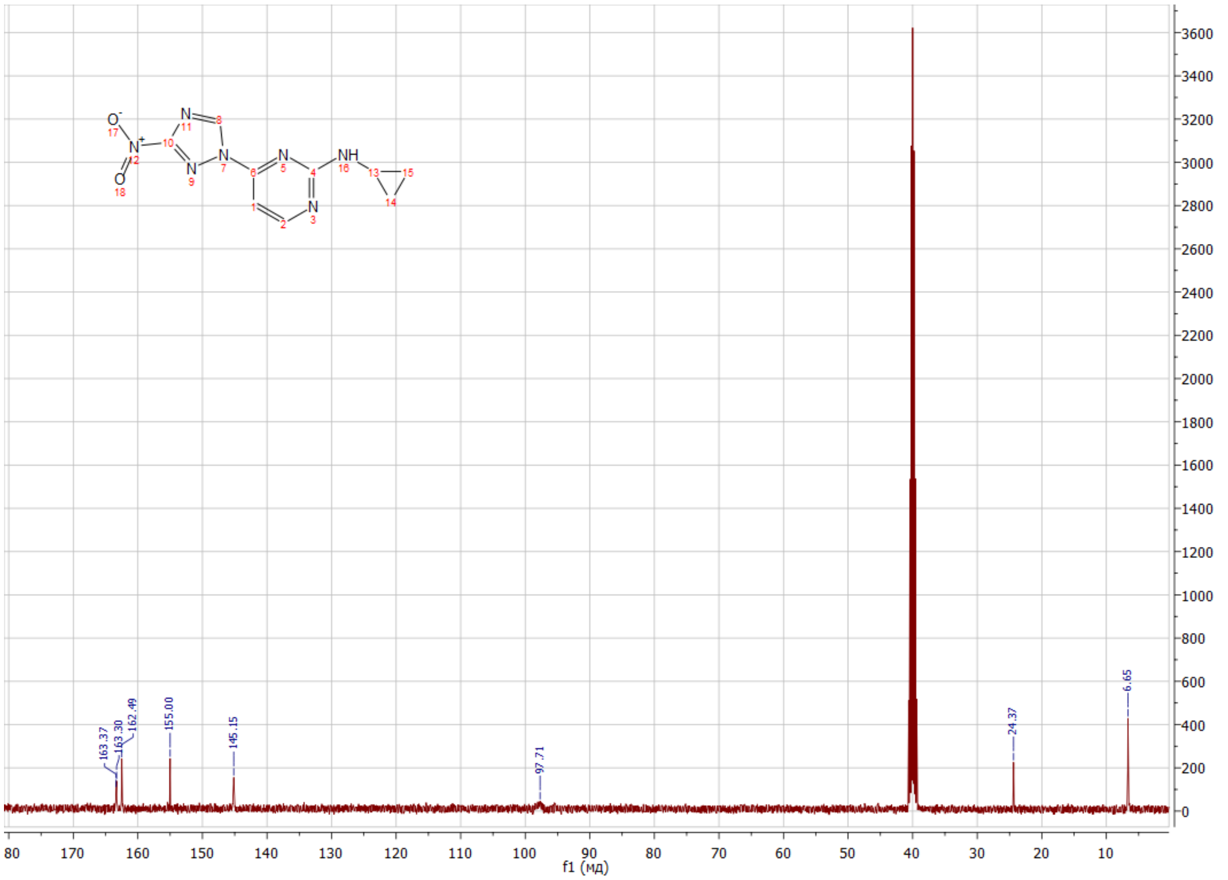

^{1}H and ^{13}C NMR of *N*-isopropyl-4-(3-nitro-1*H*-1,2,4-triazol-1-yl)pyrimidin-2-amine (2o)

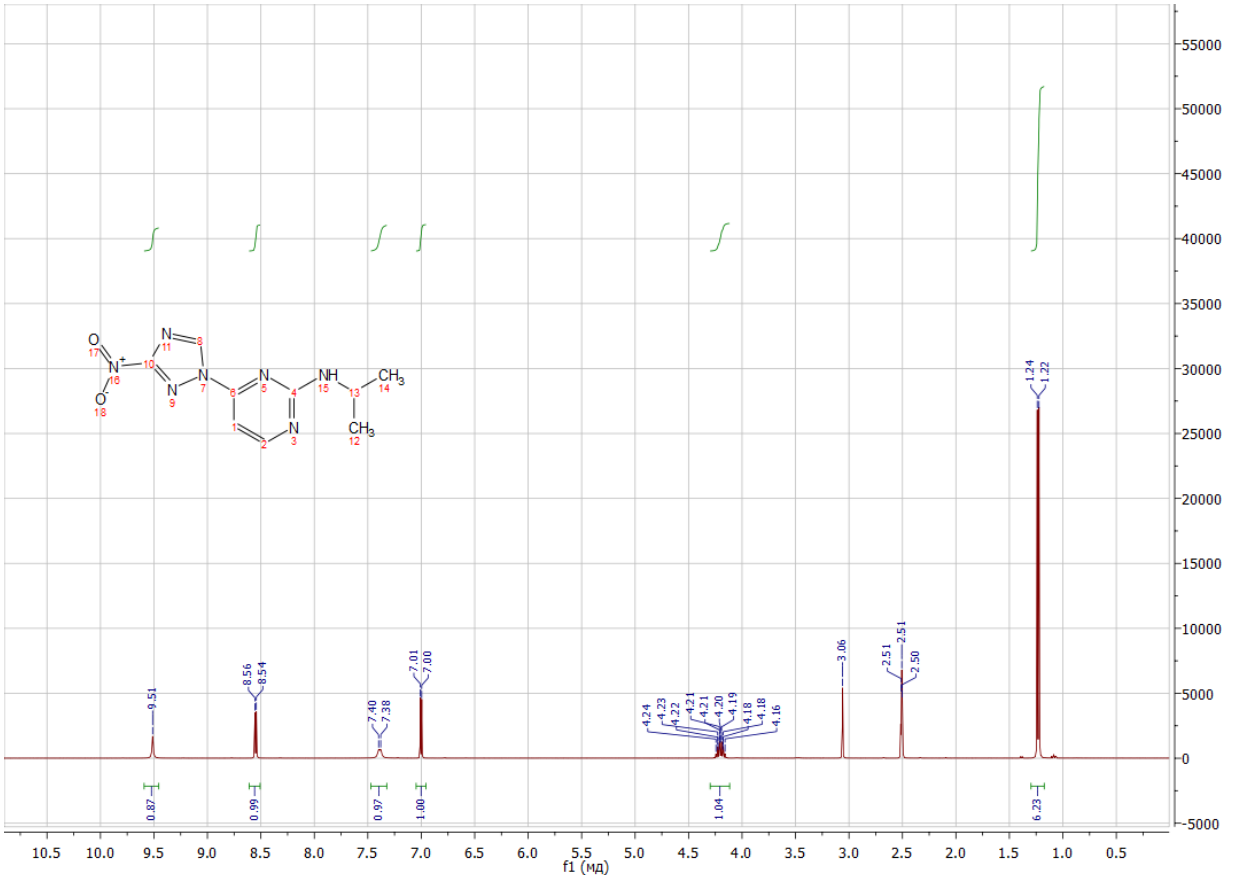

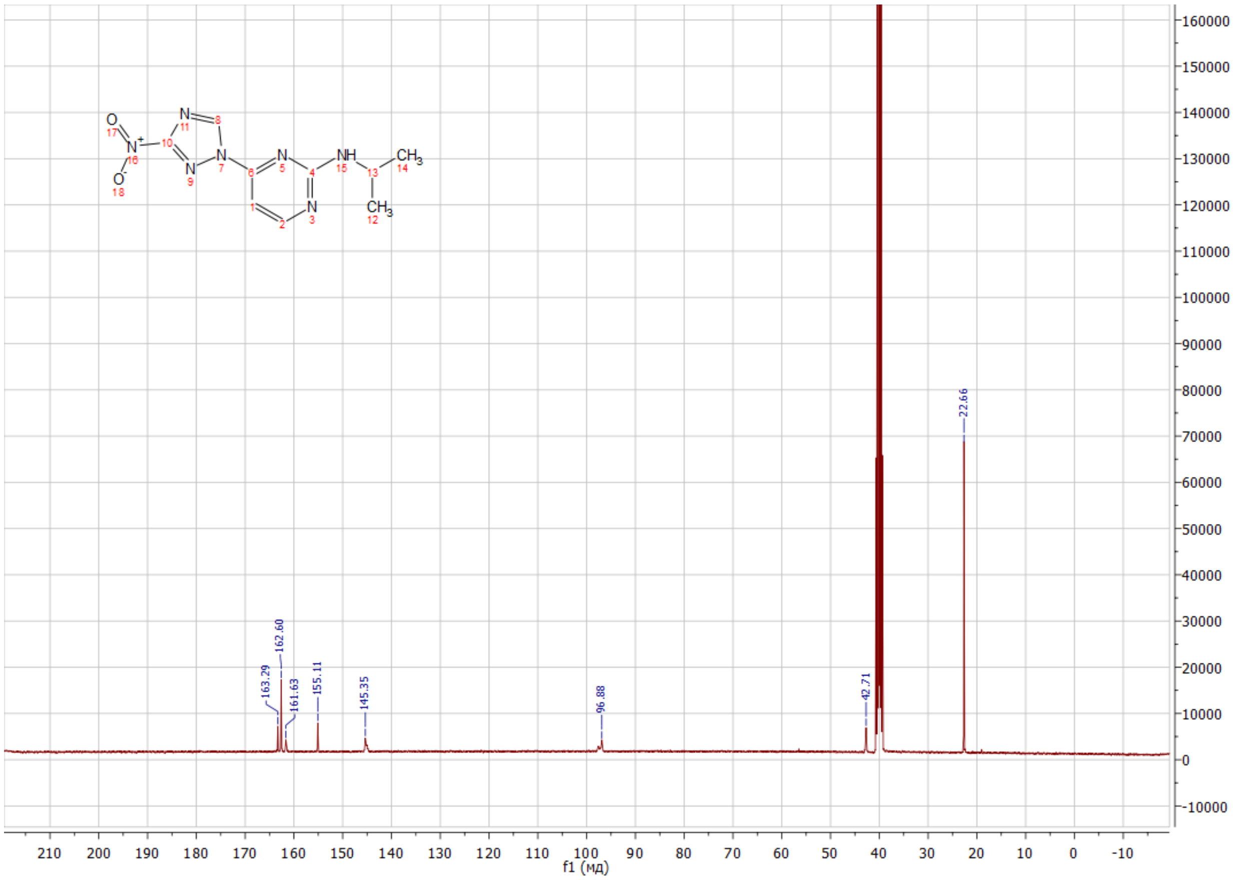

^{1}H and ^{13}C NMR of *N*-(3-(1*H*-imidazol-1-yl)propyl)-4-(3-nitro-1*H*-1,2,4-triazol-1-yl)pyrimidin-2-amine (2p)

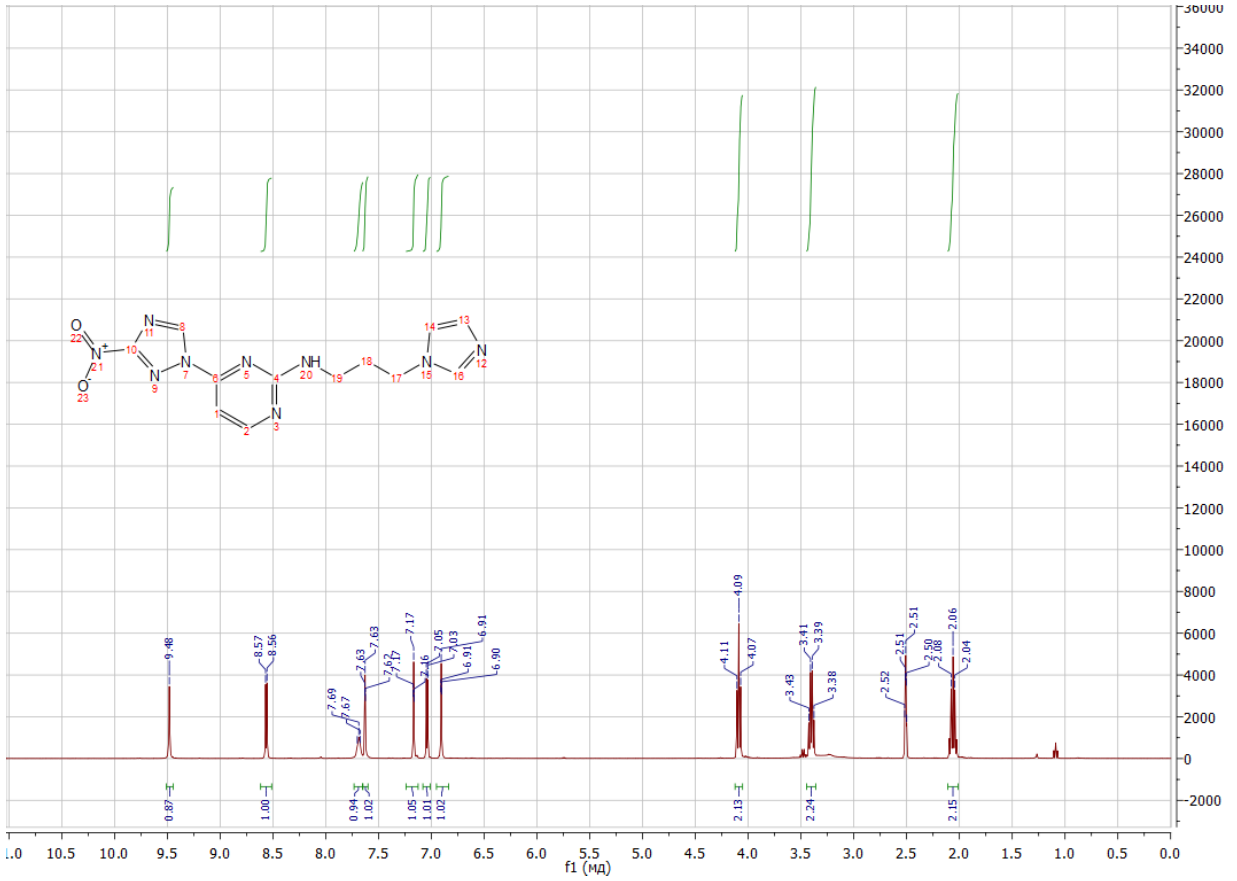

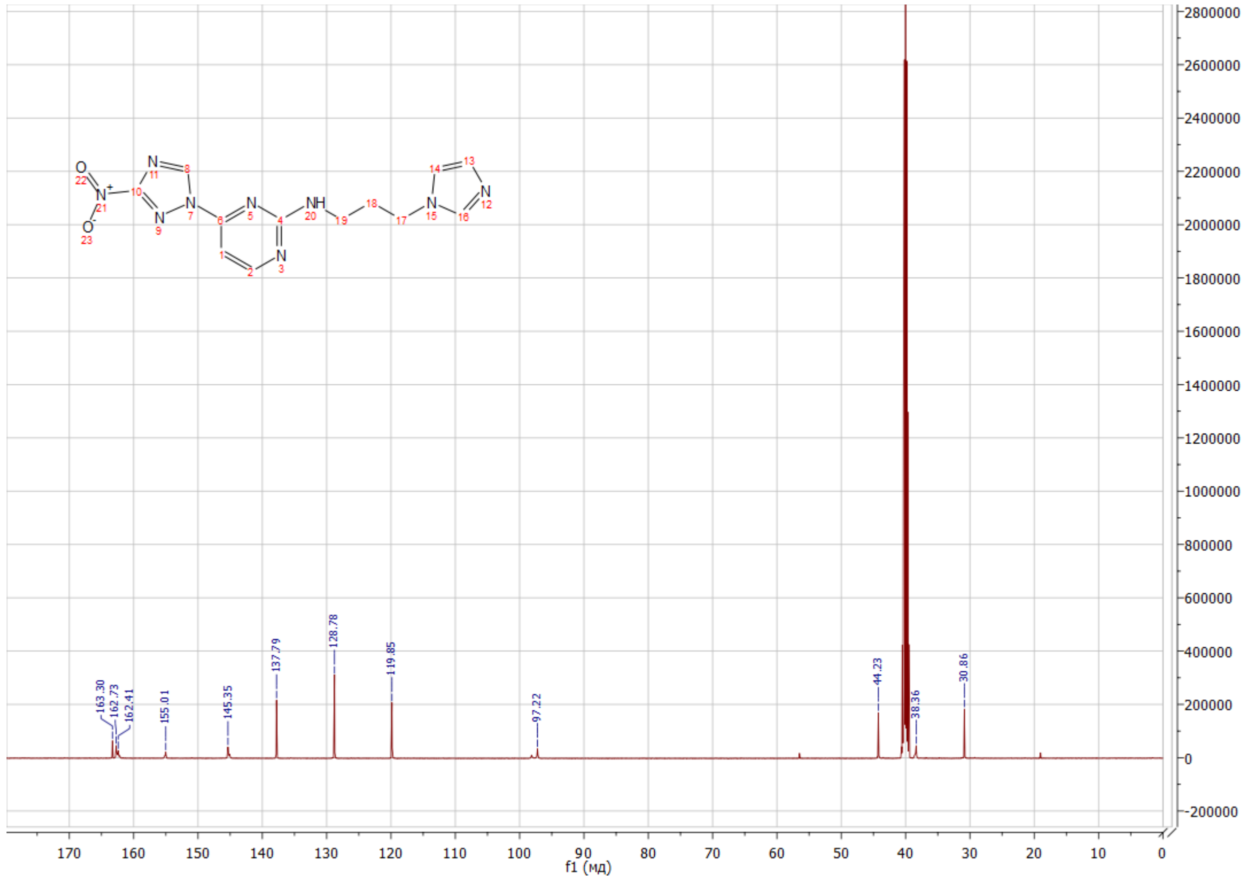

^{1}H and ^{13}C NMR of 4-(3-nitro-1*H*-1,2,4-triazol-1-yl)-2-(pyrrolidin-1-yl)pyrimidine (2q)

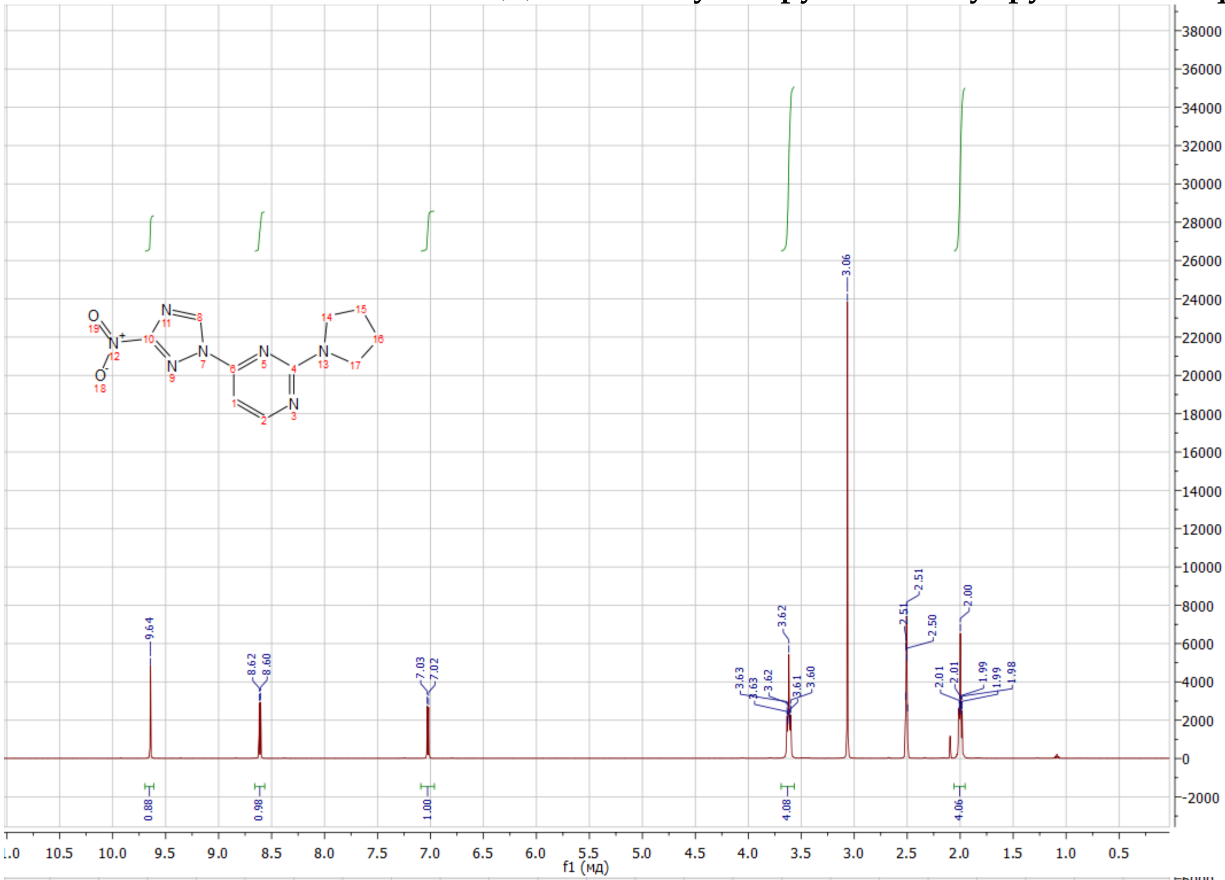

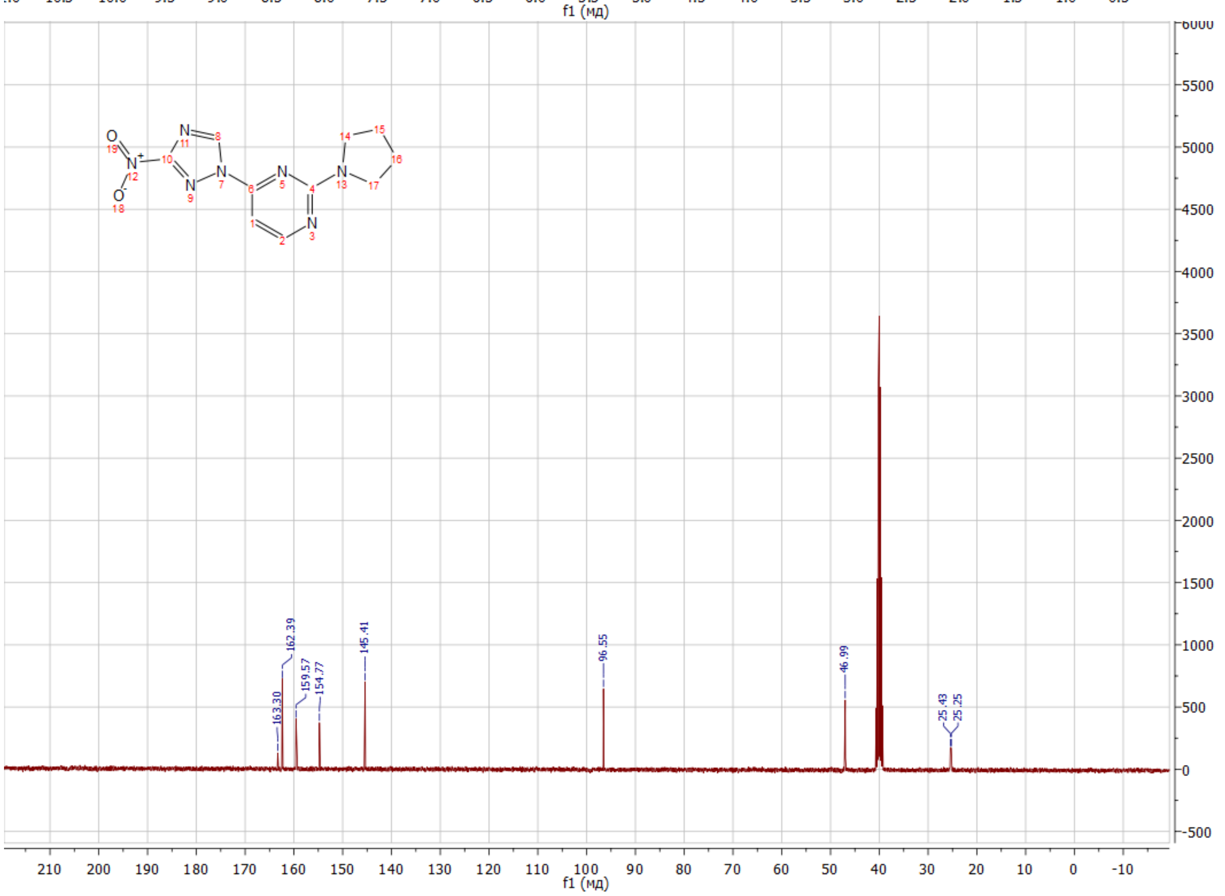

^{1}H and ^{13}C NMR of 4-(3-nitro-1*H*-1,2,4-triazol-1-yl)-*N*-propylpyrimidin-2-amine (2r)

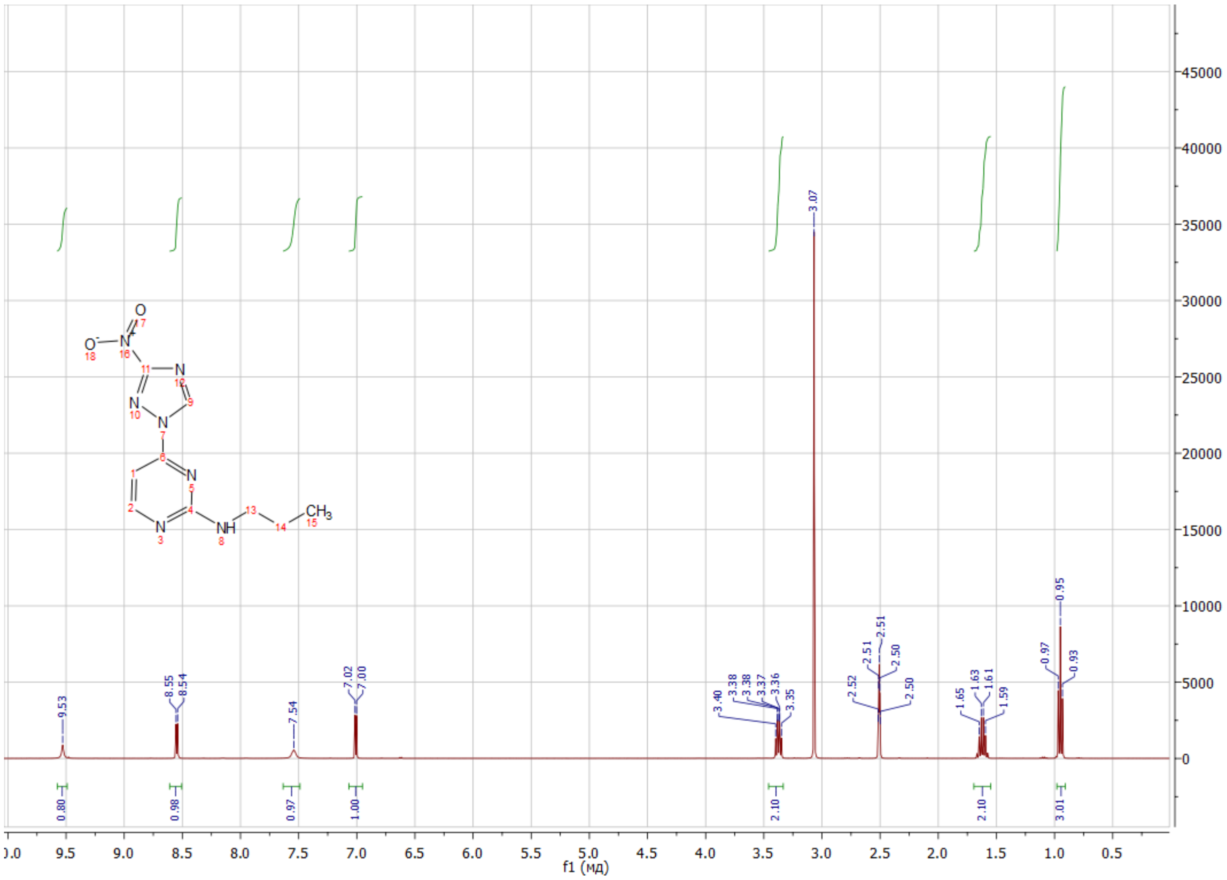

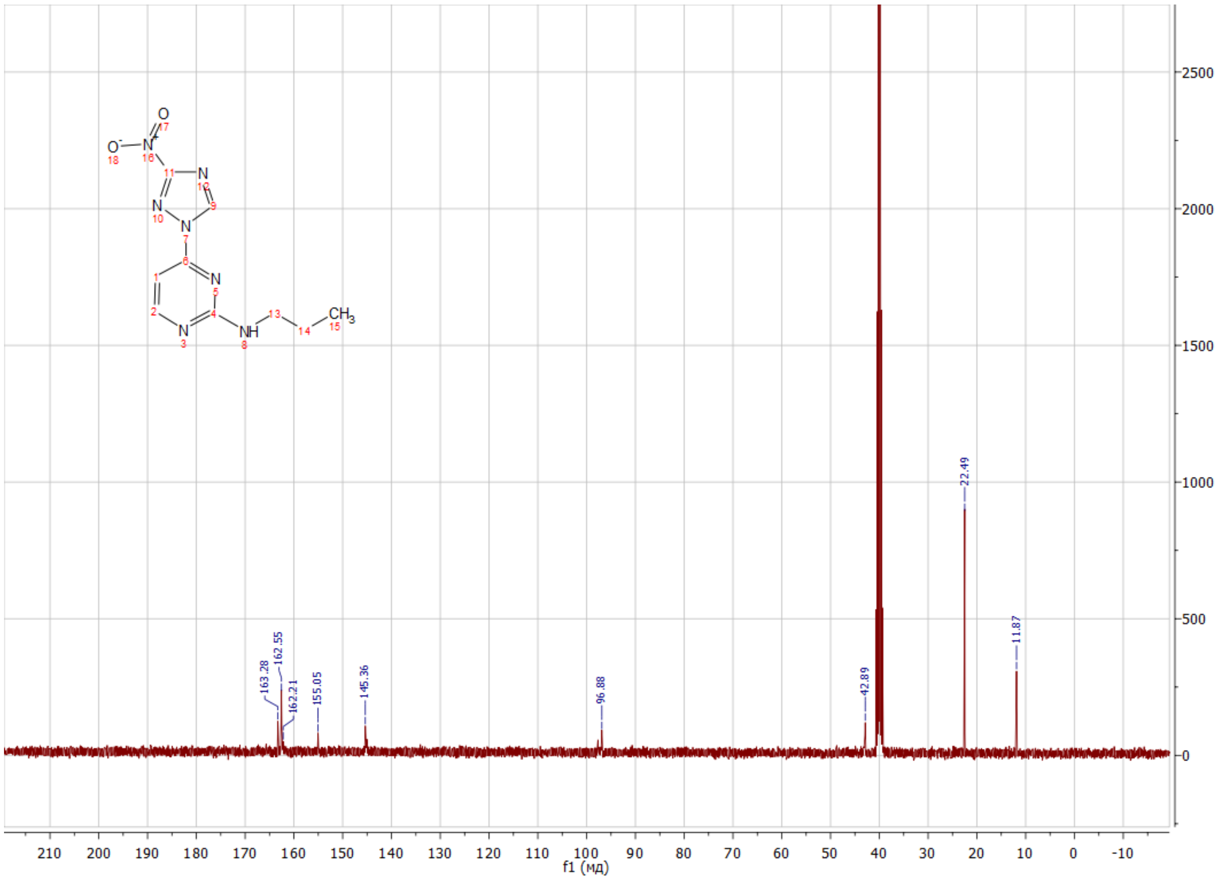

^{1}H and ^{13}C NMR of *N*-(4-methoxybenzyl)-4-(3-nitro-1*H*-1,2,4-triazol-1-yl)pyrimidin-2-amine (2s)

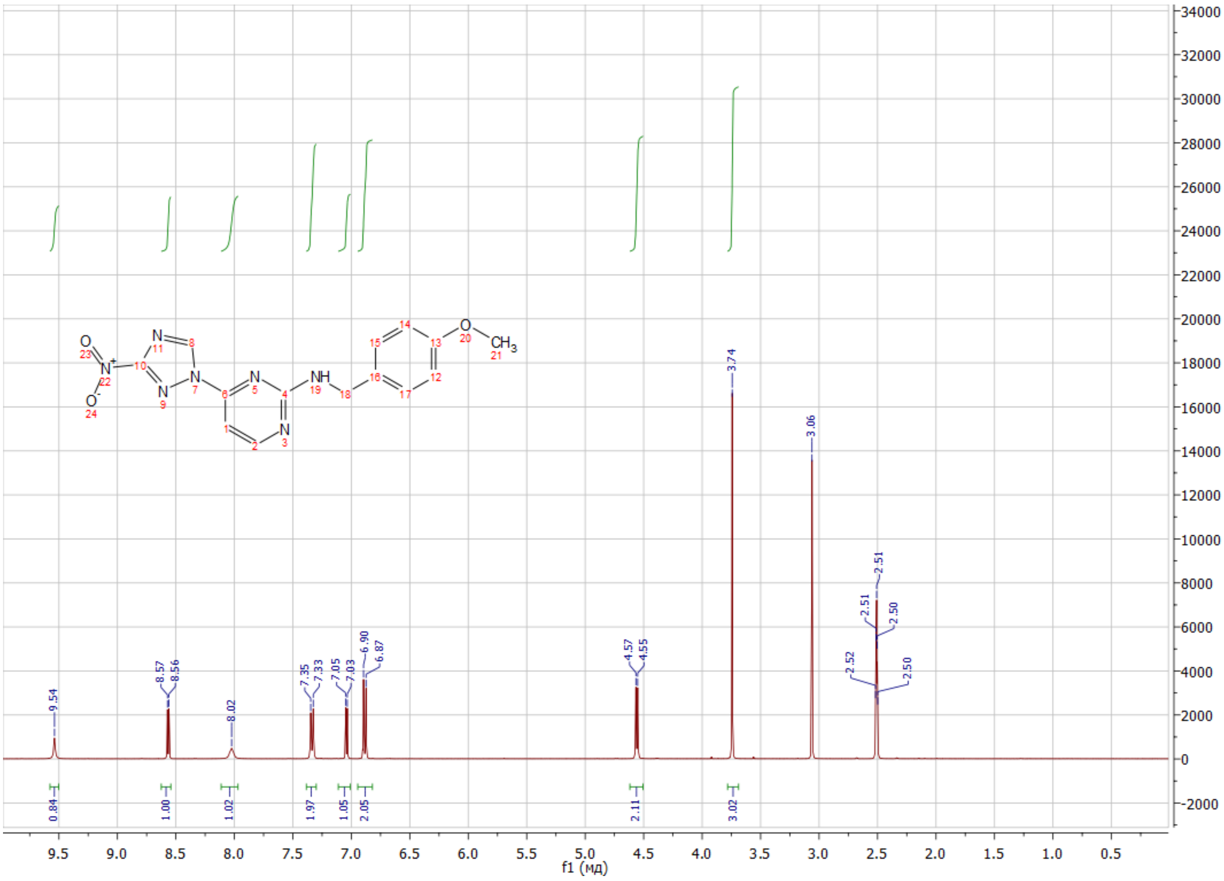

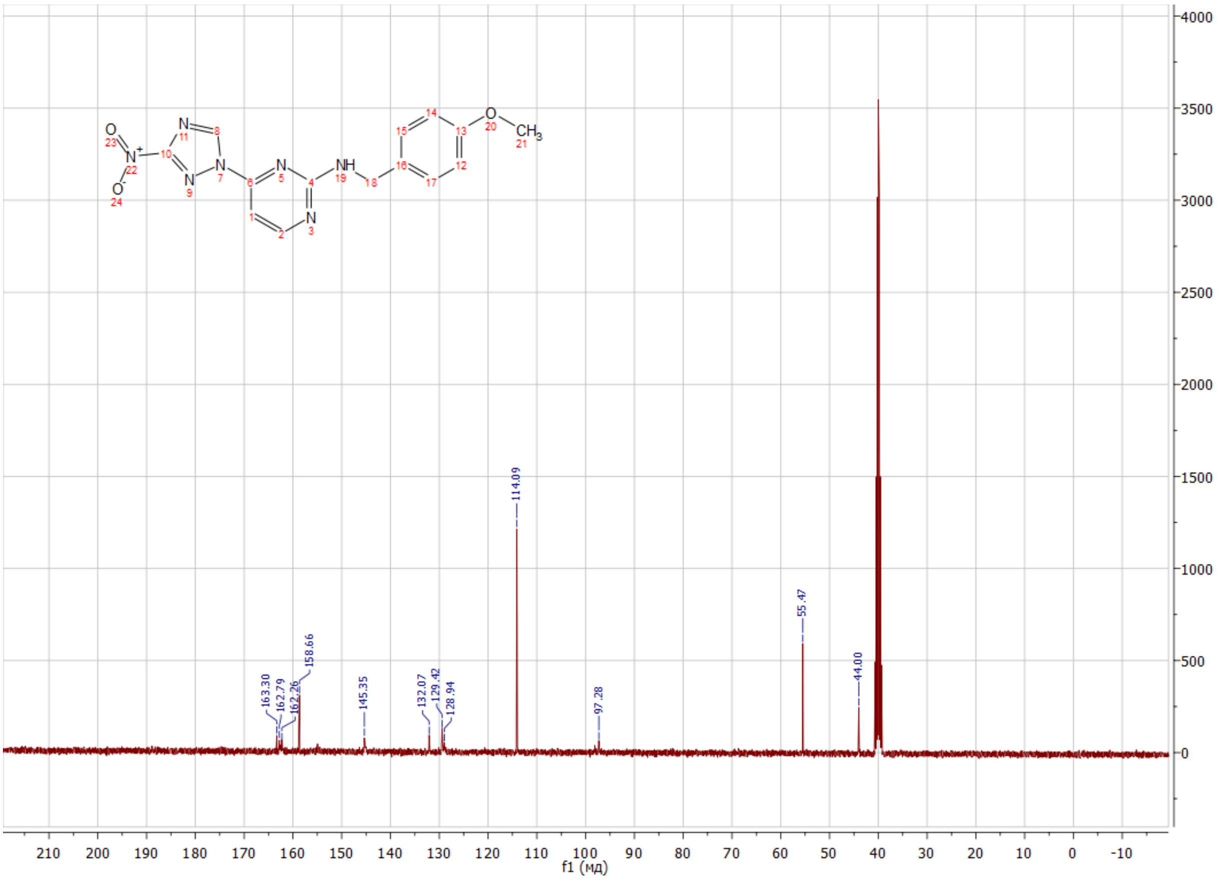

Supplement: Supplementary file 1 [file antibiotics-09-00666-s001.pdf]
